# A genome-wide cross-trait analysis identifying shared genetic basis and causal relationships between Hunner-type interstitial cystitis and autoimmune diseases in East Asian populations

**DOI:** 10.3389/fimmu.2024.1417899

**Published:** 2024-11-15

**Authors:** Xinyi Lyu, Liao Peng, Xueyuan Xu, Yang Fan, Yong Yang, Jiawei Chen, Mengzhu Liu, Yuanzhuo Chen, Chi Zhang, Shiqin Yang, Sihong Shen, Jie Zhang, Xiao Zeng, Hong Shen, Deyi Luo, Yifei Lin

**Affiliations:** ^1^ Department of Urology, Institute of Urology (Laboratory of Reconstructive Urology), West China Hospital, Sichuan University, Chengdu, Sichuan, China; ^2^ Pelvic Floor Diseases Center, West China Tianfu Hospital, Sichuan University, Chengdu, Sichuan, China; ^3^ Health Management Center, West China Hospital, Sichuan University, Chengdu, Sichuan, China; ^4^ General Practice Medical Center, West China Hospital, Sichuan University, Chengdu, Sichuan, China; ^5^ Medical Device Regulatory Research and Evaluation Center, West China Hospital, Sichuan University, Chengdu, Sichuan, China; ^6^ Department of Urology, Beijing Friendship Hospital, Capital Medical University, Beijing, China; ^7^ Institute of Urology, Beijing Municipal Health Commission, Beijing, China; ^8^ Department of Urology, Lab of Health Data Science, Innovation Institute for Integration of Medicine and Engineering, West China Hospital, Sichuan University, Chengdu, Sichuan, China; ^9^ Department of Epidemiology, Harvard T.H. Chan School of Public Health, Boston, MA, United States

**Keywords:** cross-trait analysis, genetic epidemiology, Mendelian randomization, Hunner-type interstitial cystitis, autoimmune disorder

## Abstract

**Purpose:**

Epidemiological studies have demonstrated the clinical link between Hunner interstitial cystitis (HIC) and autoimmune diseases (ADs), suggesting potential shared genetic bases for their comorbidity. We aimed to investigate the shared genetic architecture and causal relationships between HIC and ADs.

**Methods:**

We conducted a genome-wide cross-trait study with ~170000 individuals of East Asian ancestry to investigate the shared architecture between HIC and ADs. Bidirectional Mendelian randomization (MR) was used to assess potential causal relationships and a multi-trait analysis of GWAS (MTAG) was conducted to identify their associated pleiotropic loci. Fine-mapping analysis narrowed candidate gene susceptibility loci and colocalization analysis was performed to identify shared variants at specific locus. Lastly, transcriptome-wide association (TWAS) and functional analysis were utilized to explore potential shared gene-tissue associations.

**Results:**

Through bidirectional MR analysis, we observed a positive causal effect of AIH(OR_IVW_=1.09, P_IVW_=1.00×10^-3^) and RA (OR_IVW_=1.47, P_IVW_<1.00×10^-4^) on HIC and a negative causal effect of UC on HIC (OR_IVW_=0.89, P_IVW_< 1.00×10^-4^). Furthermore, we unveiled a robust positive causal effect of HIC on T1D(OR_ConMix_=1.05, P_ConMix_=1.77×10^-3^). Cross-trait meta-analysis identified a total of 64 independent SNPs associated with HIC and ADs. Functional analysis revealed that the identified variants regulated gene expression in major tissues belonging to the autoimmune system.

**Conclusions:**

Our findings might offer insights into the shared underlying etiology of HIC and ADs.

## 1 Introduction

Hunner interstitial cystitis (HIC) is a rare and challenging chronic inflammatory bladder disease of uncertain etiology. It is characterized by persistent bladder pain and lower urinary tract symptoms (LUTS), such as urinary frequency and urgency, along with the presence of Hunner ulcers on cystoscopy (1). HIC is a subtype of interstitial cystitis (IC)/bladder pain syndrome (BPS), which is a broadly defined chronic pelvic pain syndrome involving the urinary system. IC encompasses various potential etiologies and clinical phenotypes, with a global prevalence of approximately 10.6 cases per 100,000 individuals (2). The diagnosed prevalence in females is about five times higher than in males. Among IC cases, HIC accounts for 3.5% to 50% of all cases. The pathogenic mechanisms underlying HIC remain unclear, although previous studies have suggested that it involves complex interactions between multiple mechanisms, including neural, endocrine, and immune factors (1, 3). Immunoglobulin and complement deposition, aggregation of restricted light-chain plasma cells, and upregulation of pro-inflammatory genes/molecules involved in innate and adaptive immune responses have been detected in the bladder tissues of HIC patients (4–8).

Furthermore, IC may be a systemic disease and is often comorbid with various autoimmune diseases (ADs). Numerous studies have demonstrated an increased prevalence of multiple ADs in IC patients, including rheumatoid arthritis (RA), systemic lupus erythematosus (SLE), Sjögren’s syndrome (SS), inflammatory bowel disease, and autoimmune thyroid diseases (9–12).

However, due to the observational nature of traditional epidemiological studies, methodological limitations still exist in current research on the comorbidity between IC and ADs. The underlying pathological and physiological mechanisms, as well as genetic associations, particularly specific shared genetic factors and potential genetic causal effects between IC and ADs, require further investigation. Genome-wide association studies (GWAS), functional genomics research, and integrative analyses offer new avenues for studying the genetic architecture of complex diseases. These approaches can identify candidate pathogenic genes or tissue/cell types and provide insights into the development of disease-related biomarkers and targeted therapeutics.

In this study, we conducted a large-scale genome-wide cross-trait association study with ~170000 individuals of East Asian ancestry to investigate the shared architecture between HIC and ADs, including atopic dermatitis (AD), autoimmune hepatitis (AIH), allergic rhinitis (AR), asthma (AS), contact dermatitis (CD), Graves’ disease (GD), Hashimoto’s thyroiditis (HT), hypothyroidism (HY), hyperthyroidism (HYPE), myasthenia gravis (MG), pollinosis (PO), psoriasis vulgaris (PV), rheumatoid arthritis (RA), sarcoidosis (SA), systemic lupus erythematosus (SLE), Sjögren’s syndrome (SS), type 1 diabetes mellitus (T1D), ulcerative colitis (UC), uveitis (UV). We aimed to not only assess the genetic correlation and potential causal relationship between HIC and ADs but also to identify pleiotropic loci associated with joint phenotypes. We hope our findings can help better delineate the biological implications of shared genetic architecture between HIC and ADs.

## 2 Materials and methods

### 2.1 Study population, design, and data summary

**Figure 1:**
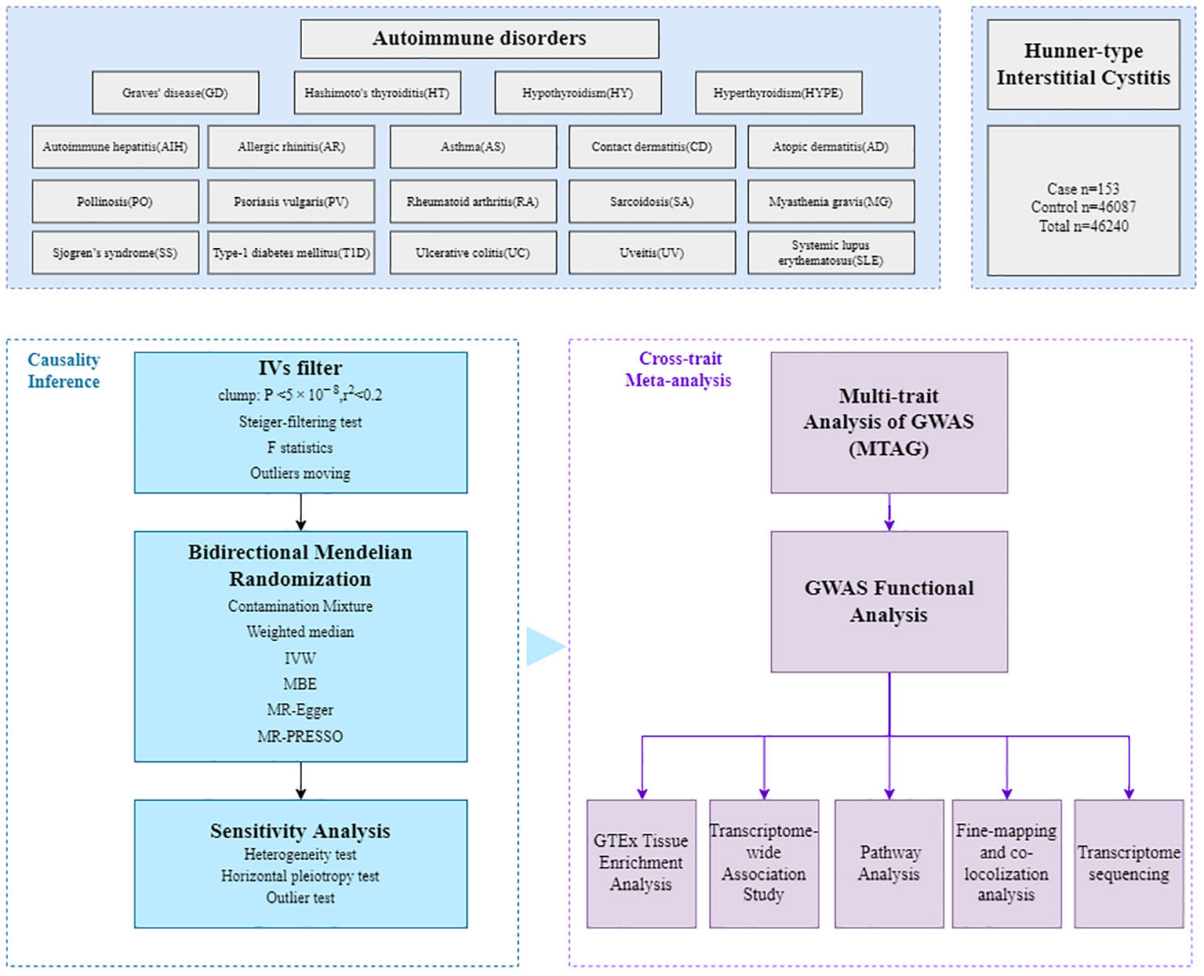
Overall study design. The GWAS summary statistics are retrieved from various published sources, then the bidirectional Mendelian randomization (MR) analyses were employed to assess potential causal relationships. The multi-trait analysis of GWAS (MTAG), combining single-trait GWAS data, was further conducted to investigate the underlying comorbidity mechanisms, followed by fine-mapping, colocalization analysis, transcriptome-wide association (TWAS), functional analysis and transcriptome sequencing.

The workflow of our analysis was shown in **Figure 1**. In brief, there were three main parts in our study: causal inference analysis, cross-trait meta-analysis and post-GWAS analysis between HIC and the 19 autoimmune disorders.

The GWAS summary statistic of HIC includes a total of 153 cases and 46,087 controls (13). The 153 cases were recruited at Tokyo University Hospital in Japan between 2018 and 2020. DNA samples of controls were obtained from the Biobank Japan Project (BBJ). The autoimmune diseases included AD(N_case_/N_control_=2472/142192, AIH(N_case_/N_control_=85/166529), AR(N_case_/N_control_=7897/153666), AS(N_case_/N_control_=13015/162933), CD(N_case_/N_control_=247/161777), GD(N_case_/N_control_=2809/172656), HT(N_case_/N_control_=537/172656), HY(N_case_/N_control_=1114/172656), HYPE(N_case_/N_control_=994/172656), MG(N_case_/N_control_=81/178630), PO(N_case_/N_control_=18593/153666), RA(N_case_/N_control_=5348/173268), SA(N_case_/N_control_=220/177667), SLE(N_case_/N_control_=317/175937), SS(N_case_/N_control_=303/175599), T1D(N_case_/N_control_=1219/132032), UC(N_case_/N_control_=314/178375), UV(N_case_/N_control_=125/174600), the GWAS summary statistics of were obtained from NBDC Human Database, with the Dataset ID hum0197.v3.gwas.v1 (14). We used ANNOVAR (15) to annotate variants of GWAS summary statistics based on ‘hg19 avsnp150’. Additional details for each dataset can be found in **Supplementary Table S1** and **Supplementary Notes**.

### 2.2 Causal inference analysis

To identify independent genetic instruments, we utilized the PLINK clumping function with the following parameters: clump-p1 = 5e-8, clump-p2 = 0.01, clump_kb=500Kb, and clump_r2 = 0.2. This allowed us to determine the top loci that were independent of each other. To ensure statistical power due to the limited number of instrumental variables (n<10), we employed more lenient criteria for instrument variable selection: clump-p1 = 5e-6, clump-p2 = 0.01, clump_kb=500Kb, and clump_r2 = 0.2. Furthermore, we applied Steiger filtering to the instrumental variables and excluded instruments with F-statistics<10.

We utilized several MR methods to examine the causal relationships between each ADs and HIC. Our primary MR analysis was the contamination mixture (ConMix) approach (16), which explicitly modeled multiple potential causal estimates and inferred multiple causal mechanisms associated with the same risk factor that affects the outcome to different degrees. Additionally, we also applied several sensitivity analyses to validate our results. The MR-PRESSO (17) was employed to remove outliers and ensure efficient use of valid IVs. MR-Egger regression (18) provided estimates after the correction of pleiotropy. The weighted-median (WM) estimator approach, as a median of the weighted estimates, provides a consistent effect even if half of the IVs are pleiotropic (19). The median-based method(MBE) proceeds by constructing a kernel-weighted density of the variant-specific estimates, and taking the maximum point of this density as the point estimate. A confidence interval is obtained by bootstrapping (20). Finally, we employed the inverse-variance weighted (IVW) method (21), which is a robust approach. We corrected multiple testing for MR P-values by the Bonferroni method, and a P-value of 0.00263 (0.05/19) was considered as the significant level. A P-value less than 0.05 is considered as the threshold indicating statistical significance. ADs that showed possible causal relationships with HIC were included in the following analyses.

We also performed several sensitivity analyses to assess the robustness of our results to potential violations of several MR assumptions. a) Heterogeneity was estimated by the Cochran Q test of IVW and MR-Egger; b) The horizontal pleiotropy was estimated using MR-Egger’s intercept; c) The influential outlier IVs due to pleiotropy was identified using MR-PRESSO’s outlier test. After removing the outlier instrumental variables (IVs) identified by MR-PRESSO, we conducted the MR analysis again.

The same approach was taken for the reverse MR which was used to eliminate spurious results due to reverse causation. Generally, all the analyses were conducted using R software 4.2.0. The MR-PRESSO method was performed using the “MRPRESSO” package. The IVW, MR–Egger, WM, ConMix and MBE methods were performed using the “MendelianRandomization” package. The forest plot of single SNP, funnel plot and scatter plot were performed using the “TwoSampleMR” package.

### 2.3 Fine-mapping credible set analysis

We performed statistical fine-mapping using FINEMAP (22). We computed LD in each locus using R package ‘LDlinkR’ (23)based on genome build GRCh37 and the East Asian population of 1000 Genome project population. We defined a fine-mapping region as the 3Mb (± 1.5Mb) window around each lead variant. This window size is based on recommendations for fine-mapping and colocalization analyses. We allowed up to 10 causal variants per window and extracted the posterior inclusion probabilities (PIP) of each variant using each method independently. The variants with PIP>0.90, along with having LD *r* ^2^>0.2 with the lead variant, are considered the final candidate causal variants. We applied 3DSNP, a comprehensive database for human noncoding variants annotation, to annotate these causal variants (24).

### 2.4 Colocalization analysis

We extracted summary statistics for variants within 500 kb(± 250kb) of the index SNP at each of the shared loci between HIC and ADs and performed colocalization analysis between HIC and each ADs trait using R ‘coloc’ package (25) to calculate the probability that the two traits shared a common genetic causal variant. We caculated the posterior probability that the 2 traits were associated with different causal variants(H3) or that the 2 traits were associated and shared 1 common causal variant(H4). The posterior probability for H3(PPH3) or H4(PPH4) that was greater than 0.5 was considered colocalized (26).

### 2.5 Cross-trait meta-analysis

We then implemented a cross-trait meta-analysis of GWAS summary data using Multi-Trait Analysis of GWAS (MTAG) (27), a method for joint analysis of summary statistics from GWASs of different traits, to identify pleiotropic loci with strong signals associated with ADs and HIC. By analyzing multiple traits together, this approach increases the statistical power of detecting genetic associations for each trait. The MTAG estimator is a variant of the IVW meta-analysis that utilizes summary statistics from single-trait GWASs and generates trait-specific associations statistics. The resulting P-values can be considered as P-values from a single-trait GWAS. We applied PLINK clumping function parameters: -clump-p 5×10^-8^, -clump-r2 0.2 -clump-kb 500, to determine top loci. The variant with the lowest p-value was defined as the sentinel variant. A P-value of 5×10^-8^ was considered as a genome-wide significance for cross-trait meta-analysis and the significant SNP should also meet a requirement that p value of 5×10^-3^ from both single traits.We then performed functional annotation by Functional Annotation of Variants-Online Resource (FAVOR) (28), an open-access variant functional annotation portal for cross-trait meta-analysis.

### 2.6 eQTL mapping, tissue enrichment analysis, and pathway analysis

To map the shared SNPs between HIC and ADs traits to specific genes which they show a significant eQTL association with, we conducted the eQTL mapping analysis using the Functional Mapping and Annotation(FUMA) website (29), incorporating the SNP2GENE function with the cis-eQTLs reported by DICE (30), which were identified in 13 immune cell types isolated from 106 leukapheresis samples provided by 91 healthy subjects, and the eQTLs reported by van der Wijst et al (31), which were identified from 25,000 peripheral blood mononuclear cells (PBMCs) from 45 donors.

To delve into the biological implications of the shared genes among the ten trait pairs, we performed GTEx tissue enrichment analysis within the clumping region for each trait identified by MTAG, using the FUMA website based on 54 tissue types sourced from GTEx(version 8) (29). Additionally, we utilized the FUMA website to assess the enrichment of independent loci for each trait pairing and to explore shared genes between ADs and HIC, examining their association with Gene Ontology (GO) and Kyoto Encyclopedia of Genes and Genomes (KEGG) terms to elucidate relevant biological pathways. The method for multiple testing correction was BH with an adjusted *p-value* cutoff (0.05).

### 2.7 Transcriptome-wide association

To explore the potential shared gene-tissue associations between ADs and HIC, we performed a TWAS using FUSION (R package), based on 49 GTEx (version 8) multi-tissue expression weights. FUSION adopts a Bayesian sparse linear mixed model (BSLMM) (32) that combines Bayesian variable selection (BVSR) (33) and linear mixed model (LMN) (34) with the normal mixture prior assumption to train weights between observed gene expressions and cis-acting genetic variants with reference dataset. This method tests the association between predicted gene expression and phenotypes of interest. Besides, we applied Benjamini-Hochberg correction for each trait’s all gene-tissue pairs on TWAS P-values, accounting for multiple tests (false discovery rate < 0.05).

### 2.8 Extraction of TPM expression matrix of genes of interest from the GEO database

The microarray datasets GSE11783 (6), GSE55235 (35), GSE206364 (36), and GSE181674 (37) were extracted from the GEO database (https://www.ncbi.nlm.nih.gov/geo/). Specifically, the GSE11783 dataset for HIC is based on the GPL570 platform, the GSE55235 dataset for RA is based on the GPL96 platform, the GSE206364 dataset for AIH is based on the GPL20301 platform, and the GSE181674 dataset for T1D is based on the GPL21290 platform. We selected the TPM expression matrix of the genes of interest and performed a Wilcoxon rank-sum test to compare gene expression levels between the control and disease groups. The inclusion details of the samples in each dataset are provided in **Supplementary Table S2**.

## 3 Results

### 3.1 Causal inference between HIC and ADs

**Figure 2:**
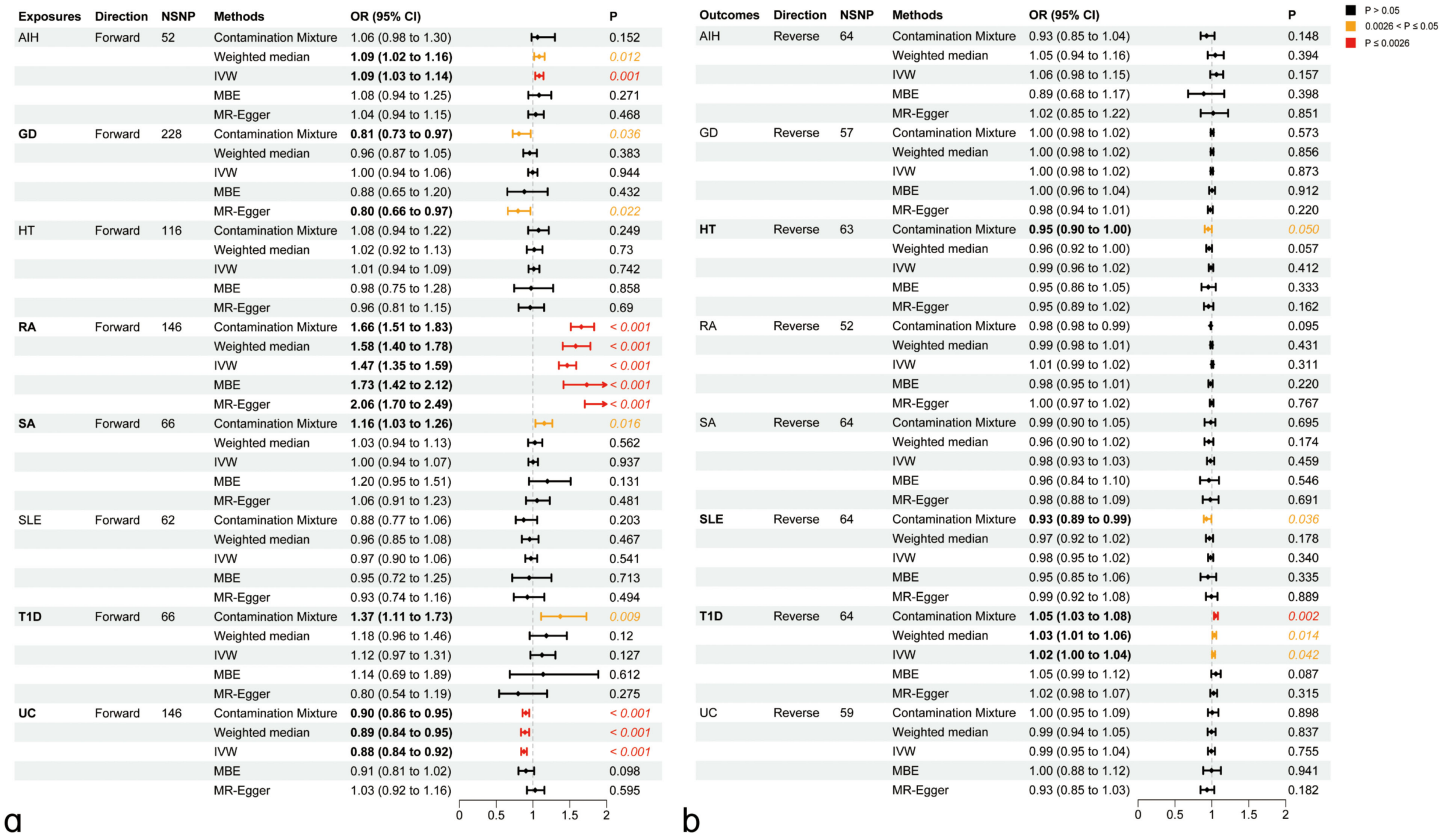
Causal inference between ADs on HIC using bidirectional Mendelian Randomization (MR) analysis. **(A)** Causal effect of ADs on HIC. **(B)** Causal effect of HIC on ADs. AIH, Autoimmune hepatitis; GD, Graves’ disease; HT, Hashimoto’s thyroiditis; RA, Rheumatoid arthritis; SA, Sarcoidosis; SLE, Systemic lupus erythematosus; T1D, Type 1 diabetes mellitus; UC, Ulcerative colitis; IVW, Inverse-­variance weighted method; MBE, mode-­based estimate.

We explored the genetic relationship between HIC and ADs using Bidirectional MR analyses to gain additional insights into the genetic connections between these diseases. Bidirectional MR analyses were first addressed to investigate the causal relationship between HIC and ADs. The details were presented in **Supplementary Tables S3-4**; **Figure 1**. Based on forward MR analysis, we found that ADs might have a potential causal effect on HIC. Specifically, genetically predicted AIH (OR_IVW_=1.09, P_IVW_=1.00×10^-3^) and RA (OR_IVW_=1.47, P_IVW_<1.00×10^-4^) exhibited significant positive causal effect on HIC. This result was further validated in sensitivity analyses using other MR methods. The forward MR analysis using the ConMix approach revealed potential positive causal effects of SA (OR_ConMix_=1.16, P_ConMix_=1.63×10^-2^) and T1D (OR_ConMix_=1.37, P_ConMix_=9.22×10^-3^) on HIC despite not meeting the threshold of FDR correction. However, the reverse MR analysis revealed significant positive causal effects of HIC on T1D (OR_ConMix_=1.05, P_ConMix_=1.77×10^-3^), suggesting that HIC is likely a risk factor of T1D (**Figure 2**).

Genetically predicted UC (OR_IVW_=0.89, P_IVW_< 1.00×10^-4^) has a significant negative causal effect on the risk of HIC, which was agreed by other MR methods (P_WM_<1.20×10^-4^, P_ConMix_=3.07×10^-3^) and sensitivity analysis using MR-PRESSO (OR_PRESSO_=0.89, P_PRESSO_=2.23×10^-5^). Additionally, a forward MR analysis using the ConMix and Egger approach revealed a potential negative causal effect of GD on HIC (OR_ConMix_=0.81, P_ConMix_=3.56×10^-2^; OR_Egger_=0.80, P_ConMix_=2.22×10^-2^).

Reverse MR analysis revealed a positive causal effect of HIC on RA. However, after correction using the outlier test in MR-PRESSO, the aforementioned causal effects are no longer significant. Additionally, the reverse analysis revealed potential negative causal effects of HIC on HT (OR_ConMix_=0.95, P_ConMix_=4.98×10^-2^) and SLE (OR_ConMix_=0.92, P_ConMix_=3.58×10^-2^) after removing the outliers identified by the PRESSO analysis (**Figure 2**). The detailed results of the sensitivity analysis for MR can be found in **Supplementary Table S5** and **Supplementary Figures S1-116**.

### 3.2 Cross-trait meta-analysis between HIC and ADs

**Figure 3:**
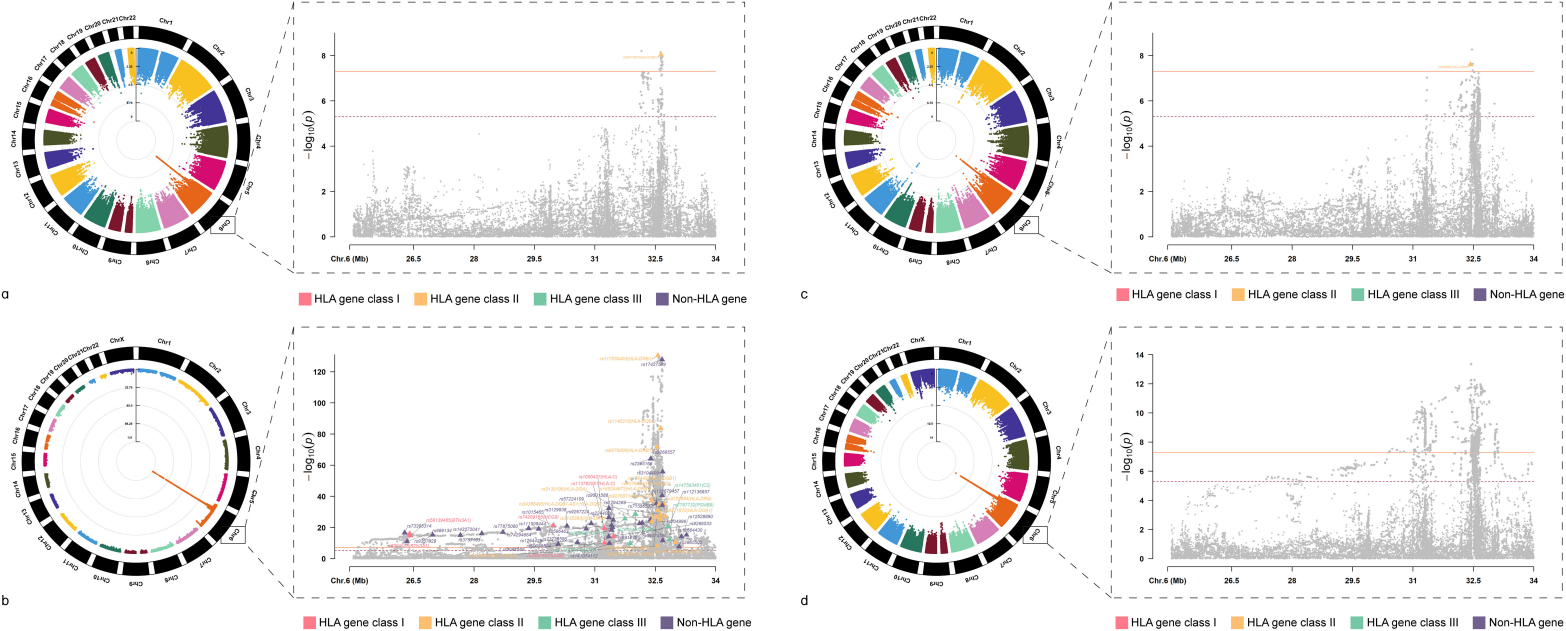
Circular Manhattan Plot of Multi-Trait Analysis for HIC and ADs GWAS meta-analysis. **(A)** Manhattan plot for cross-trait meta-analysis between AIH and HIC; **(B)** Manhattan plot for cross-trait meta-analysis between RA and HIC; **(C)** Manhattan plot for cross-trait meta-analysis between T1D and HIC; **(D)** Manhattan plot for cross-trait meta-analysis between UC and HIC; AIH, Autoimmune hepatitis; RA, Rheumatoid arthritis; T1D, Type 1 diabetes mellitus; UC, Ulcerative colitis; HIC, Hunner-type interstitial cystitis.

After investigating the causal relationships between HIC and ADs, we conducted cross-trait meta-analyses to identify individual SNPs underlying the joint phenotypes based on MTAG(all these SNPs fulfilled P_single_<5×10^-3^, P_MTAG_<5×10^-8^) to combine the association evidence for HIC with ADs (**Table 1** and **Figure 3**).

**Table 1:** Cross-trait meta-analysis between HIC and Ads.

Trait pair	SNP	CHR	Position	ADs		HIC		MTAG		Gencode Comprehensive Category	Nearest Gene	Coloc^a^		Nearby gene transcription level	
				BETA	P1	BETA	P2	BETA	P3			PPH3>0.5	PPH4>0.5	HIC	ADs
HIC&AIH	rs564176274	6	32642025	0.92	3.23E-05	0.72	1.62E-05	0.02	9.40E-09	intergenic	HLA-DQB1	yes	no	stable	up
HIC&RA	rs117530403	6	32566482	0.79	2.53E-150	0.5	3.29E-03	0.1	2.91E-131	intergenic	HLA-DRB1	yes	no	unknown	unknown
	rs17427599	6	32667364	0.6	1.24E-138	0.61	9.90E-06	0.08	1.58E-128	intergenic	AL662789.1	yes	no	unknown	unknown
	rs1140310	6	32632783	0.45	7.21E-83	0.73	6.50E-08	0.06	2.30E-84	exonic	HLA-DQB1	yes	no	stable	up
	rs9270000	6	32552740	0.44	5.03E-80	0.38	4.71E-03	0.05	2.04E-72	intronic	HLA-DRB1	yes	no	unknown	unknown
	rs2395166	6	32388275	-0.41	2.13E-70	-0.39	2.64E-03	-0.06	6.37E-65	intergenic	TSBP1-AS1	yes	no	unknown	unknown
	rs9268557	6	32389305	0.36	1.48E-68	0.41	5.85E-04	0.05	8.50E-65	intergenic	TSBP1-AS1	yes	no	unknown	unknown
	rs3104409	6	32683121	0.35	3.49E-58	0.46	2.52E-04	0.05	1.48E-56	intergenic	AL662789.1	yes	no	unknown	unknown
	rs138679457	6	32667423	1.01	1.05E-42	1.27	2.42E-03	0.12	2.68E-41	intergenic	AL662789.1	yes	no	unknown	unknown
	rs3129884	6	32410210	-0.33	8.45E-37	-0.56	1.78E-04	-0.05	6.03E-38	intronic	HLA-DRA	yes	no	up	up
	rs71536532	6	32523756	0.31	4.51E-37	0.41	3.99E-03	0.04	5.20E-36	intergenic	RNU1-61P	yes	no	unknown	unknown
	rs9271872	6	32595418	-0.34	5.16E-36	-0.45	1.70E-03	-0.05	1.01E-35	upstream	HLA-DQA1	yes	no	up	up
	rs9276571	6	32725620	0.28	1.42E-35	0.42	1.15E-03	0.04	1.30E-35	exonic	HLA-DQB2	yes	no	stable	up
	rs1794269	6	32673894	0.26	1.54E-35	0.37	2.29E-03	0.04	4.32E-35	intergenic	AL662789.1	yes	no	unknown	unknown
	rs2244020	6	31347451	-0.24	4.92E-31	-0.46	9.66E-05	-0.04	4.22E-33	intergenic	AL671883.3	yes	no	unknown	unknown
	rs145244672	6	32556461	-0.35	2.35E-31	-0.56	1.20E-03	-0.05	7.59E-32	intronic	HLA-DRB1	yes	no	unknown	unknown
	rs34386495	6	32626730	-0.44	4.99E-28	-0.76	6.54E-04	-0.06	3.02E-29	upstream;downstream	HLA-DQB1-AS1;HLA-DQB1	yes	no	unknown	up
	rs2856451	6	32011358	0.23	2.66E-28	0.39	1.10E-03	0.03	3.56E-29	intronic	TNXB	no	no	stable	down
	rs9276653	6	32746414	0.3	2.05E-26	0.57	4.49E-04	0.05	5.25E-28	intergenic	HLA-DQB2	yes	no	stable	up
	rs3135393	6	32408842	-0.31	6.79E-25	-0.7	7.31E-05	-0.05	1.36E-27	intronic	HLA-DRA	yes	no	up	up
	rs114015773	6	32537468	-0.43	7.21E-26	-0.79	5.84E-04	-0.06	2.33E-27	intergenic	HLA-DRB1	yes	no	unknown	unknown
	rs9501588	6	31347020	-0.29	2.71E-25	-0.59	2.97E-04	-0.04	3.25E-27	intergenic	AL671883.3	yes	no	unknown	unknown
	rs909267	6	31746548	-0.39	1.26E-24	-0.77	4.66E-04	-0.06	2.36E-26	intronic	VARS	no	no	unknown	unknown
	rs58770498	6	32631029	-0.43	1.58E-24	-0.82	5.41E-04	-0.06	3.52E-26	intronic	HLA-DQB1	yes	no	stable	up
	rs1794493	6	32639578	-0.21	2.61E-24	-0.41	7.19E-04	-0.03	8.07E-26	intergenic	HLA-DQB1	yes	no	stable	up
	rs6912701	6	32383573	-0.31	3.37E-25	-0.51	2.88E-03	-0.05	8.69E-26	intergenic	TSBP1-AS1	yes	no	unknown	unknown
	rs9267224	6	31454406	-0.24	5.71E-25	-0.4	2.97E-03	-0.03	1.46E-25	ncRNA_intronic	AL645933.3	no	no	unknown	unknown
	rs62405787	6	32414473	-0.32	3.21E-21	-0.83	3.06E-05	-0.05	1.48E-24	intergenic	HLA-DRA	yes	no	up	up
	rs6907458	6	32171288	-0.42	4.17E-22	-0.79	1.06E-03	-0.06	1.37E-23	intronic	NOTCH4	no	no	stable	stable
	rs204999	6	32109979	-0.41	2.27E-22	-0.7	2.63E-03	-0.06	2.64E-23	intergenic	PRRT1	no	no	unknown	unknown
	rs57224109	6	30909461	-0.39	1.79E-21	-0.78	6.48E-04	-0.06	2.79E-23	intronic	MUCL3,SFTA2	yes	no	stable	unknown
	rs2596463	6	31413726	-0.38	5.69E-21	-0.73	1.57E-03	-0.06	2.48E-22	ncRNA_exonic	LINC01149	yes	no	stable	unknown
	rs74209185	6	29967538	-0.36	8.22E-20	-0.82	2.89E-04	-0.05	3.76E-22	intergenic	HCG9	no	no	stable	stable
	rs3129838	6	30306553	0.36	3.33E-20	0.71	1.32E-03	0.05	9.98E-22	intronic	TRIM39,TRIM39-RPP21	no	no	unknown	unknown
	rs4148878	6	32822186	-0.39	4.03E-20	-0.74	2.00E-03	-0.06	2.01E-21	intronic	PSMB9	yes	no	up	up
	rs73728586	6	31435428	-0.34	5.70E-19	-0.73	7.48E-04	-0.05	6.87E-21	ncRNA_intronic	HCP5	yes	no	unknown	up
	rs113792081	6	31235775	-0.28	7.13E-18	-0.67	3.53E-04	-0.04	3.11E-20	downstream	HLA-C	yes	no	up	up
	rs74294664	6	29355277	-0.34	9.59E-18	-0.81	3.74E-04	-0.05	4.38E-20	intronic	OR5V1	no	no	stable	unknown
	rs141074112	6	30782977	0.51	9.31E-19	1	3.11E-03	0.07	6.23E-20	ncRNA_intronic	LINC00243	yes	no	unknown	unknown
	rs111508444	6	29603512	-0.35	1.18E-17	-0.79	6.10E-04	-0.05	9.20E-20	intergenic	GABBR1	no	no	stable	stable
	rs60056504	6	31415258	-0.34	5.30E-18	-0.64	3.87E-03	-0.05	4.02E-19	ncRNA_intronic	HCP5	yes	no	unknown	up
	rs77875080	6	28716551	-0.32	1.51E-15	-0.79	6.41E-04	-0.05	9.38E-18	intergenic	AL662890.1	no	no	unknown	unknown
	rs73395314	6	26276214	-0.29	7.92E-15	-0.79	3.58E-04	-0.05	2.49E-17	intergenic	HIST1H4H	no	no	unknown	unknown
	rs3757183	6	28191859	-0.32	3.75E-15	-0.72	2.00E-03	-0.05	7.75E-17	upstream	ZSCAN9	no	no	stable	stable
	rs6901118	6	26399586	-0.33	3.10E-15	-0.71	2.64E-03	-0.05	8.88E-17	intergenic	BTN3A1	no	no	up	up
	rs12664430	6	33272677	0.28	9.51E-12	1.16	4.13E-06	0.05	5.85E-16	intronic	TAPBP	yes	no	up	up
	rs9357029	6	26970402	0.32	2.17E-14	0.72	2.87E-03	0.05	5.99E-16	ncRNA_intronic	LINC00240	no	no	stable	unknown
	rs143272041	6	27661068	-0.31	4.04E-14	-0.71	2.40E-03	-0.05	8.78E-16	upstream	AL009179.1	no	no	unknown	unknown
	rs56139485	6	26417851	-0.31	4.98E-14	-0.67	3.70E-03	-0.05	1.74E-15	intergenic	BTN3A1	no	no	up	up
	rs9267352	6	31463128	-0.16	5.86E-13	-0.41	1.15E-03	-0.03	5.04E-15	intronic	MICB	no	no	up	up
	rs2855502	6	33149308	-0.23	2.43E-10	-1.1	9.01E-07	-0.04	5.93E-15	intronic	COL11A2	yes	no	stable	stable
	rs9266533	6	31341777	-0.26	3.56E-12	-0.8	2.27E-04	-0.04	6.09E-15	ncRNA_intronic	AL671883.3	yes	no	unknown	unknown
	rs7767732	6	32853277	-0.18	1.52E-11	-0.42	4.97E-03	-0.03	5.11E-13	intergenic	PSMB9	yes	no	up	up
	rs112136957	6	32682165	0.24	8.64E-10	0.82	3.82E-04	0.04	2.28E-12	intergenic	AL662789.1	yes	no	unknown	unknown
	rs989134	6	26336224	0.21	1.14E-09	0.63	1.44E-03	0.03	9.62E-12	intergenic	AL021917.1	no	no	unknown	unknown
	rs3135196	6	32997577	-0.25	2.41E-07	-1.48	2.67E-06	-0.05	2.44E-11	intergenic	HLA-DOA	yes	no	up	stable
	rs1264429	6	30565101	-0.19	3.19E-09	-0.57	2.24E-03	-0.03	3.93E-11	downstream	ABCF1	no	no	stable	down
	rs1050437	6	31239585	-0.13	2.82E-09	-0.35	4.19E-03	-0.02	6.28E-11	exonic	HLA-C	yes	no	up	up
	rs3094588	6	31362341	-0.15	2.33E-08	-0.5	9.52E-04	-0.02	1.35E-10	ncRNA_exonic	AL645933.2	yes	no	unknown	unknown
	rs147593461	6	31877763	0.42	1.22E-08	1.37	2.81E-03	0.07	1.81E-10	intronic	C2	no	no	up	up
	rs1015465	6	30086340	-0.14	3.45E-08	-0.45	2.45E-03	-0.02	4.47E-10	intergenic	TRIM31-AS1	no	no	unknown	unknown
	rs34017414	6	32554085	0.15	2.94E-06	0.7	1.81E-04	0.02	6.02E-09	intronic	HLA-DRB1	yes	no	unknown	unknown
	rs12528890	6	33089603	0.12	2.20E-04	1.05	1.91E-07	0.03	2.01E-08	intergenic	HCG24	yes	no	unknown	unknown
HIC&T1D	rs9268831	6	32427748	0.19	4.26E-06	0.37	1.81E-03	0.02	2.52E-08	intergenic	HLA-DRA	yes	no	up	up

P1 is the autoimmune disorders’ single-trait P value, P2 is the HIC single-trait P value, P3 is the P value of MTAG analysis. *Due to the large number of shared independent SNPs between the HIC-RA trait pair; ^a^Colocalization analysis was conducted based on three windows: ± 250kb, a posterior probability for H3 (PPH3) or H4 (PPH4) greater than 0.5 was considered indicative of colocalization.

We identified a total of 64 independent SNPs. For HIC and AIH, in total, we identified 1 independent SNPs. The most significant SNP (rs564176274, P_MTAG_= 9.40× 10^− 9^, P_HIC_= 1.62 × 10^− 5^, P_AIH_= 3.23 × 10^− 5^) was located at the intergenic region, which was near gene *HLA-DQB1.*For HIC and RA, we identified 62 shared independent SNPs. The most significant SNP (rs117530403, P_MTAG_= 2.91×10^-131^, P_HIC_= 3.29×10^-3^, P_RA_= 2.53×10^-150^) was located near gene *HLA-DRB1*.Notably, there were three significant SNPs located in the exonic regions of *HLA-DQB1, HLA-DQB2 and HLA-C*. For HIC and T1D, we identified 1 shared independent SNPs. The most significant SNP (rs9268831, P_MTAG_= 2.52×10^-8^, P_HIC_= 1.81×10^-3^, P_T1D_= 4.26×10^-6^) was located near gene *HLA-DRA*. For HIC and UC, We did not identify any SNP that meets the significance threshold.

### 3.3 Fine-mapping to identify potential causal variants and colocalization analysis

In fine-mapping potential causal variants underlying HIC and ADs shared signals detected in the GWAS meta-analysis, we nominate 5 putative causal variants at 1 locus, 286 causal variants at 62 loci, 5 causal variants at 1 locus shared between AIH, RA, T1D and HIC, respectively (**Supplementary Tables S6-8**). Further colocalization analysis was conducted to ascertain whether the genetic variants driving the association in 2 traits are the same or different.We found 41 loci colocalized at different causal variants within 500 kb(± 250kb) of the lead SNP. The only shared loci between HIC and AIH and T1D demonstrated colocalization at distinct causal variants (**Supplementary Table S9**).

### 3.4 Tissue-specific enrichment analysis, pathway analysis, and eQTL mapping

The GTEx enrichment analysis independent tissue expression was significantly enriched (FDR < 0.05) for expression of cross-trait associated genes for HIC-RA, which included whole blood, spleen, brain, and small intestine. However, no significant tissue enrichment was identified for HIC-AIH, HIC-UC, and HIC-T1D meta-analysis (**Supplementary Figures S117-120**).

In terms of GO, we observed that pathways were primarily associated with antigen processing and presentation, immune processes mediated by lymphocytes, leukocytes, B cells, and T cells, production of immunoglobulins, and regulation of cell cytotoxicity, among others. Additionally, in KEGG pathways, we identified enrichment of shared loci between HIC and autoimmune diseases in immune response processes such as antigen processing and presentation, allograft rejection, intestinal immune network for IgA production, natural killer cell-mediated cytotoxicity, as well as autoimmune diseases including graft-versus-host disease, type 1 diabetes mellitus, autoimmune thyroid disease, viral myocarditis, asthma, systemic lupus erythematosus, and leishmania infection(**Supplementary Tables S10-13**, **Supplementary Figures S121-128**).

To explore whether the significant SNPs shared between HIC and ADs had a downstream functional impact, we extracted the eQTL data corresponding to the significant SNPs found in each HIC and ADs trait pair from the immune cell eQTL data and the PBMCs eQTL data. Among the 62 shared SNPs between HIC and RA (**Supplementary Table S14**), we identified 10 SNPs associated with the expression of nearby genes. Notably, increased expression was observed for the symbol genes *BTN2A2, BTN3A1, FLOT1, DDR1, HLA-DQA2, HLA-DQB2, HLA-DRB5, HLA-DQA1, and HLA-DPA1.* For HIC and T1D (**Supplementary Table S15**), we identified 1 SNP (rs9262670) associated with the increased expression of *HLA-DQA2* and *HLA-DQB2* in monocytes, CD4 T cells, CD8 T cells and B cells.

### 3.5 Shared genes between HIC and ADs from TWAS

We then examined shared TWAS genes between HIC and ADs in specific tissues. After BH correction, we identified 5 TWAS-significant genes between HIC with RA, 4 genes for UC and 2 for T1D (**Supplementary Tables S16-18**). Notably, the cross-trait meta-analysis of HIC-RA has identified *HLA-DOA* as genome-wide significant (rs3135196, P_MTAG_= 2.44×10^-11^, P_HIC_= 2.67.00×10^-6^, P_RA_= 2.41×10^-7^). *HLA-DPB1* was also significant in the cross-trait meta-analysis of HIC and RA (rs12528890, P_MTAG_= 2.01×10^-8^, P_HIC_= 1.91×10^-7^, P_RA_= 2.20×10^-4^). Notably, although *PSMB8* is not significant in the MTAG analysis, both *PSMB8* and *PSMB9* belong to the gene encoding the proteasome 20S subunit. Additionally, *PSMB9* is significant in the MTAG analysis of HIC-RA.

### 3.6 The transcriptomic expression levels of shared genes between HIC and ADs.

**Figure 4:**
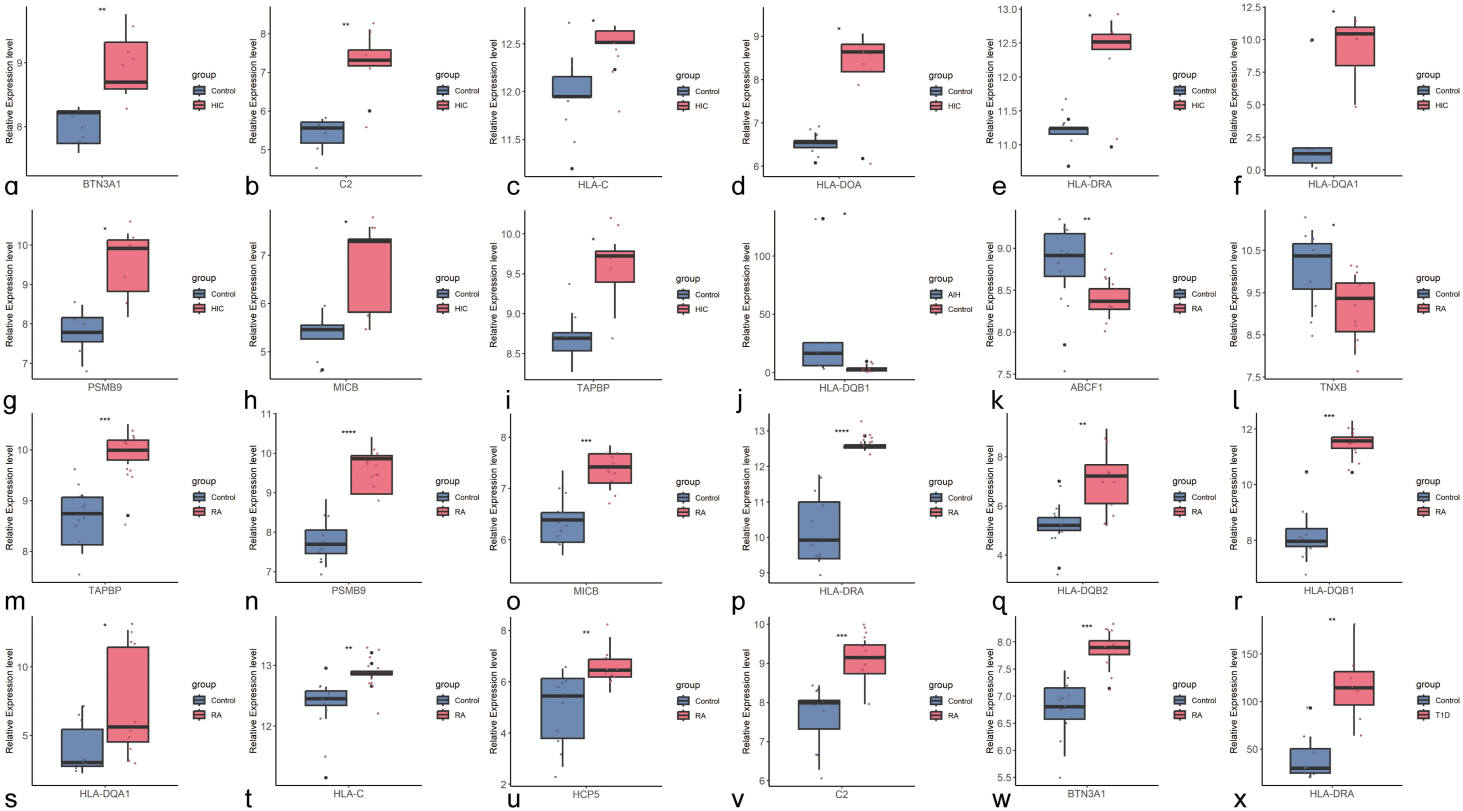
Boxplots of Shared Genes with Significant Differential Expression Between Control and Disease Groups **(A–I)** Boxplots of shared genes with significant differential expression between HIC and the control group (*BTN3A1, C2, HLA-C, HLA-DOA, HLA-DRA, HLA-DOA1, PSMB9, MICB, TAPBP*); **(J)**. Boxplot of the shared gene with significant differential expression between AIH and the control group (*HLA-DQB1*); **(K–W)**. Boxplots of shared genes with significant differential expression between RA and the control group (*ABCF1, TNXB, TAPBP, PSMB9, MICB, HLA-DRA, HLA-DQB2, HLA-DQB1, HLA-BQA1, HLA-C, HCP5, C2, BTN3A1*); **(X)**. Boxplot of the shared gene with significant differential expression between T1D and the control group.

We further validated the differences in the expression of shared genes between the disease and control groups at the transcriptomic level (**Figure 4**). After excluding pseudogenes and different transcripts, we found that among the shared genes identified through cross-trait meta-analysis, *HLA-DQB1*, a shared gene between HIC and AIH, was upregulated only in AIH, but the difference was not statistically significant in HIC (**Supplementary Figure S129**). *HLA-DRA* was significantly upregulated in HIC, RA, and T1D. Seven shared genes between HIC and RA trait pairs, including *BTN3A1, C2, HLA-C, HLA-DQA1, PSMB9, MICB*, and *TAPBP*, were significantly upregulated in both HIC and RA (P<0.05). However, *HLA-DOA* was upregulated only in HIC, while *HLA-DQB2*, *HLA-DQB1*, and *HCP5* were upregulated only in RA. Additionally, *ABCF1 and TNXB* were significantly downregulated only in RA.

## 4 Discussion

We conducted, to the best of our knowledge, the first comprehensive assessment of the shared genetics between HIC and ADs in the East Asian population by analyzing large-scale GWAS summary data using multiple statistical genetic approaches. Through bidirectional MR analysis, we investigated the causal relationships between HIC and several ADs. Notably, we observed a significant positive causal effect of AIH and RA on HIC, which is consistent with previous epidemiological findings. A nationwide population-based study conducted in Taiwan also indicated an association between IC/BPS and the development of RA. Furthermore, our study unveiled a robust positive causal effect of HIC on T1D. In contrast, in the MR analysis, we observed a significant negative causal effect of UC on HIC, which diverges from previous epidemiological reports (12).

Moreover, several ADs, including GD, SA, and SLE, demonstrated statistical significance in the bidirectional MR analysis with HIC. However, these results did not pass the multiple corrections. Interestingly, although previous epidemiological reports have suggested a common comorbidity between Sjögren’s syndrome and IC, we did not observe a significant correlation in our causal inference. This discrepancy might be due to the relatively small sample size of GWAS studies on Sjögren’s syndrome.

We also identified 64 independent loci shared between HIC and AIH, RA, and T1D at genome-wide significant level. We highlighted HLA region (several sentinel SNPs) for its significant role in between HIC and ADs. HLA region harbors more than 200 genes located close to each other on chromosome 6, one of the most extensively studies regions in human genome that contains abundant pleiotropy for many complex diseases, especially involved in the immune-related process (38). The genome-wide association study has identified that three amino acid positions in human leukocyte antigen *HLA-DQB1* and one amino acid position in *HLA-DPB1* were associated with the increased risk of HIC, which revealed that genetic contributions to HIC risk that may be associated with class II MHC molecule antigen presentation. Notably, there is no significant difference in the transcriptional levels of *HLA-DQB1* and *HLA-DPB1* between the HIC and control groups, suggesting that the associated risk SNPs may contribute to disease by affecting protein function rather than regulating gene transcription levels. Furthermore, Tseng et al. compared global gene expression profiles in bladder epithelial cells between patients with HIC and normal controls, and observed upregulations of major histocompatibility complex (MHC) class I(*HLAF*) and class II (*HLA-F, HLA-DQB1, HLA-DRB1, HLA-DPA1, HLA-DOA, HLA-DMA and HLA-DRA*) molecules in bladder epithelial from IC and ulcerative IC area.

In this study, we also confirmed that *HLA-DQB1* is the most significant locus in the MTAG analysis of HIC-AIH. Additionally, we have uncovered distinct potential susceptibility loci between HIC and different ADs, with the majority of these loci being located within the HLA region mentioned above. Specifically, we emphasized several genes, including overlapping loci across trait pairs such as *HLA-DRB1, HLA-DRA* and two genes that were significant in both MTAG and TWAS analysis, namely *HLA-DOA* and *PSMB9*.

*HLA-DRA* is the most significant loci shared between HIC and T1D, and it also exhibited significance between HIC and RA. *HLA-DRA* is one of the *HLA* class II alpha chain paralogues, playing a crucial role in antigen presentation (39). Notably, *DRA* lacks polymorphisms in the peptide binding region and acts as the sole alpha chain for *DRB1, DRB3, DRB4*, and *DRB5*. The HLA-DR4 (DRB1*0405–DQB1*0401) and HLA-DR9 (DRB1*0901–DQB1*0303) haplotypes were primarily associated with T1D in East Asian populations (40). Genetic variation at the *HLA-DRB1* gene is associated with RA, and HLA-DRB1*0404, *0405 have also been found to exhibit strong associations with RA in Asians (41).

*HLA-DOA*, a member of the HLA class II alpha chain paralogues, exhibited a significant association between HIC and RA, as well as in TWAS analyses of HIC-RA, HIC-T1D and HIC-UC. *HLA-DOA* forms a heterodimer with *HLA-DOB*. The heterodimer, *HLA-DO*, is localized in lysosomes of B cells and regulates *HLA-DM*-mediated peptide loading on MHC class II molecules (42). Okada et al. found that *HLA-DOA*, a non-classical HLA gene, was an independent risk factor on ACPA-positive RA and demonstrated a cis-eQTL effect of the causal variant in Japanese population (43). Furthermore, *HLA-DOA* has also been confirmed as a susceptibility locus within MHC with a moderate contribution to T1D that is independent of *HLA-DRB1* locus (44).

*PSMB9* and *PSMB8* encode beta subunits of the proteasome and are located within the class II region of the MHC. The proteasome is primarily responsible for cleaving class I MHC peptides in an ATP/ubiquitin-dependent process within a non-lysosomal pathway (45). Li et al. identified *PSMB9* as a diagnostic marker for RA using a machine learning approach (46). Furthermore, we also observed *AL662789.1* and *AL671883.3*, which are human DNA sequences derived from clone XXbac-254F23 on chromosome 6 and clone CH501-248L24 on chromosome 6, respectively. These sequences were found to be shared by more than one pair of HIC and ADs. However, there is currently limited research on these genes.

This study possesses several notable strengths. Firstly, most of the cross-trait studies now focus on the European population, and this is the first analysis to identify the shared genetic architecture of HIC and ADs using a large-scale observational GWAS dataset consisting exclusively of East Asian samples, after identifying ten GWAS sources (details were shown in **Supplementary Table S1**). Furthermore, we utilized multi-omics statistical methods such as MTAG and TWAS to identify novel genes and pathways associated with both HIC and ADs. The novel genes we discovered may serve as potential drug targets for the treatment of the disease, although further validation is required. Notably, they could offer new diagnostic and therapeutic avenues for patients with HIC, particularly those with comorbid autoimmune disorders.

We would like to acknowledge several potential limitations in our study. Firstly, the limited sample size of participants with each mental disorder and the restriction to individuals of East Asian ancestry have limited the statistical power of our GWAS analysis, which may limit the generalizability of our findings to other ancestral populations. Secondly, the lack of GWAS data for other subtypes of IC, such as non-ulcerative IC, has hindered our ability to investigate and differentiate the genetic associations between different subtypes of interstitial cystitis and immune-related disorders. Thirdly, it is important to consider common non-genetic risk factors for the occurrence of hypersensitivity HIC and ADs, such as medication and environmental factors. Our current study focused solely on evaluating shared genetic factors between HIC and ADs, and future research should investigate shared environmental factors between these conditions.

## 5 Conclusion

Our study provides evidence of a genetic correlation and causality between HIC and ADs. We identified genetic loci associated with both HIC and autoimmune disorders, as well as potential causal relationships between disease trait pairs, thereby enriching our understanding of HIC and shedding light on the shared genetic etiology of HIC and autoimmune disorders. In addition, we discovered multiple potential common biological mechanisms that can enhance our knowledge of the link between HIC and ADs. These discoveries open up new avenues for future research on functional validation, disease prevention, and clinical treatment strategies.

## Data Availability

The original contributions presented in the study are included in the article/**Supplementary Material**. Further inquiries can be directed to the corresponding authors.

## Glossary

HIC: Hunner-type interstitial cystitis
AD: Atopic dermatitis
ADs: Autoimmune disorders
AIH: Autoimmune hepatitis
AR: Allergic rhinitis
AS: Asthma
ATP: Adenosine triphosphate
BH: Benjamini-Hochberg correction
CD: Contact dermatitis
ConMix: Contamination mixture method
eQTL: Expression quantitative trait locus
FDR: False Discovery Rate correction
FUMA: Functional Mapping and Annotation of GWAS
GD: Graves’ disease
GO: Gene Ontology
GTEx: Genome-Tissue Expression project
GWAS: Genome-wide association studies
HLA: Human leukocyte antigen
HT: Hashimoto’s thyroiditis
HY: Hypothyroidism
HYPE: Hyperthyroidism
IC/BPS: Interstitial cystitis/bladder pain syndrome
IVW: Inverse-variance weighted method
KEGG: Kyoto Encyclopedia of Genes and Genomes
LD: Linkage disequilibrium
LUTS: Lower urinary tract syndrome
MBE: Meidan-based method
MG: Myasthenia gravis
MHC: Major histocompatibility complex
MR: Mendelian randomization
MR-PRESSO: Mendelian Randomization Pleiotropy RESidual Sum and Outlier
MTAG: Multi-trait analysis of GWAS
NBDC: National Bioscience Database Center
OR: Odds ratio
PBMC: Peripheral blood mononuclear cell
PIP: Posterior inclusion probabilities
PO: Pollinosis
PV: Psoriasis vulgaris
RA: Rheumatoid arthritis
SA: Sarcoidosis
SLE: Systemic lupus erythematosus
SNP: Single nucleotide polymorphism
SS: Sjögren’s syndrome
T1D: Type 1 diabetes mellitus
TWAS: Transcriptome-wide association studies
UC: Ulcerative colitis
UV: Uveitis
WM: Weighted-medium
