# Supplementary material for: A genome-wide cross-trait analysis identifying shared genetic basis and causal relationships between Hunner-type interstitial cystitis and autoimmune diseases in East Asian populations
Source: Front Immunol. 2024 Nov 15;15:1417899. doi: 10.3389/fimmu.2024.1417899 (PMC11604611; doi:10.3389/fimmu.2024.1417899)
Supplement: Supplementary file 2 [file DataSheet2.docx]

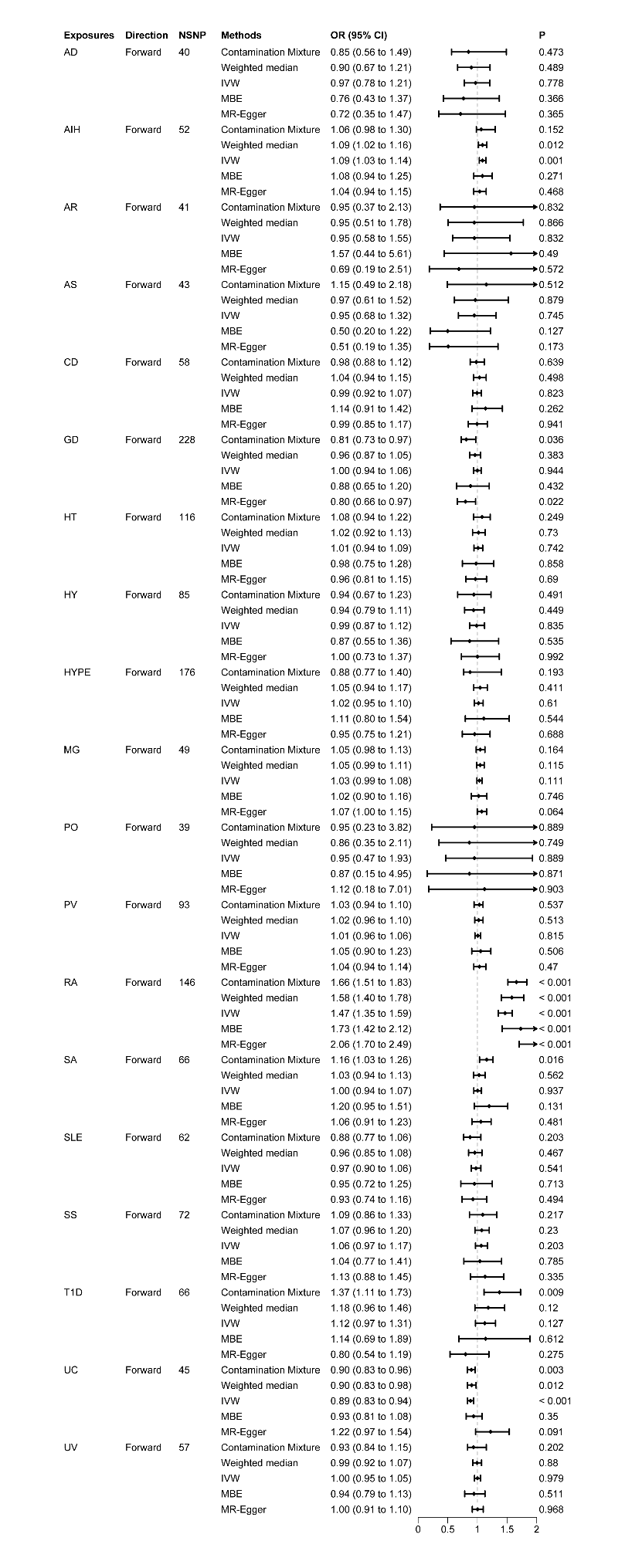


Fig S1. Causal inference of ADs on HIC using Mendelian Randomization (MR) analysis


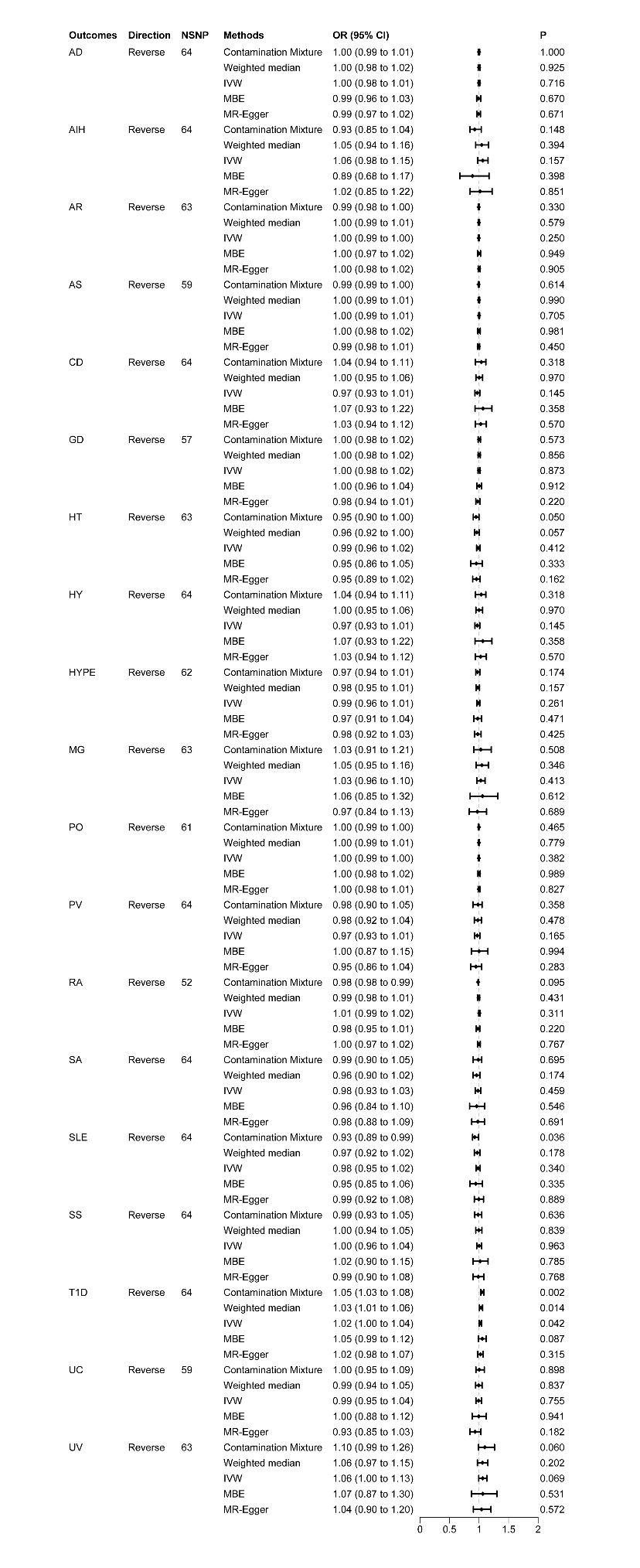


Fig S2. Causal inference of HIC on ADs using Mendelian Randomization (MR) analysis


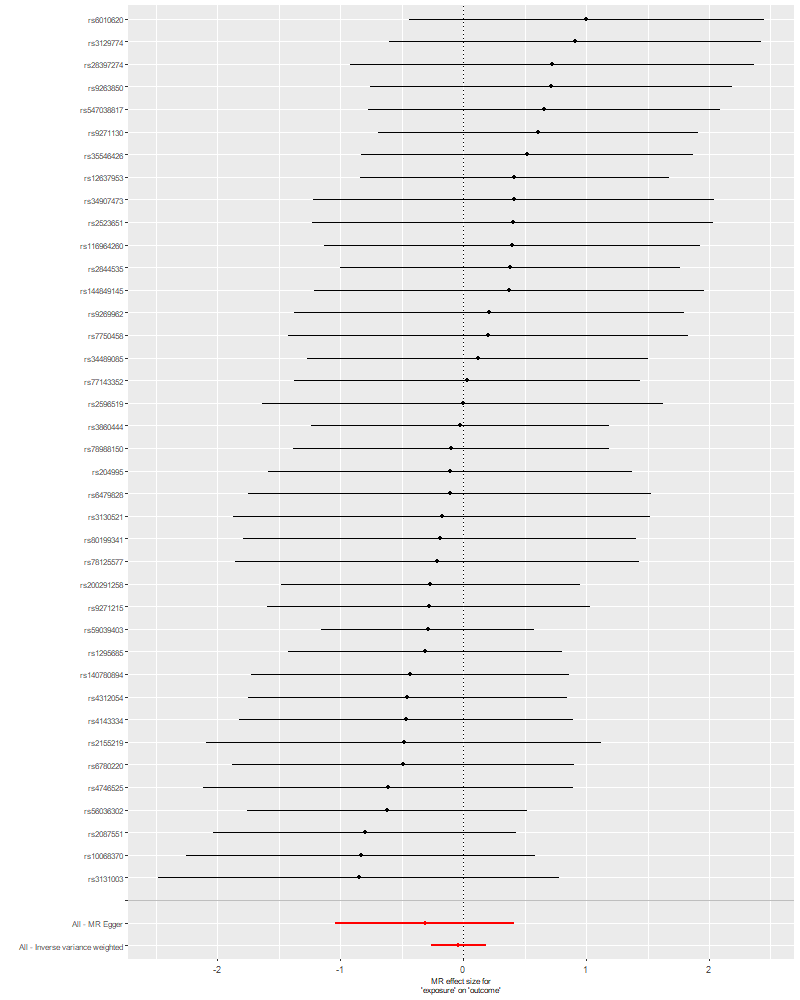


Fig S3. The forest plot of single snp for the forward MR analysis of AD on HIC.


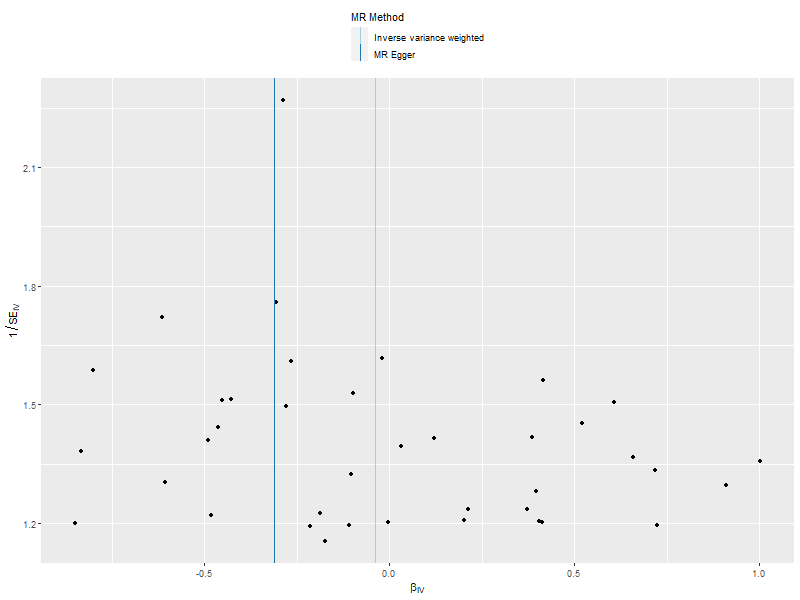


Fig S4. The funnel plot for the forward MR analysis of AD on HIC.


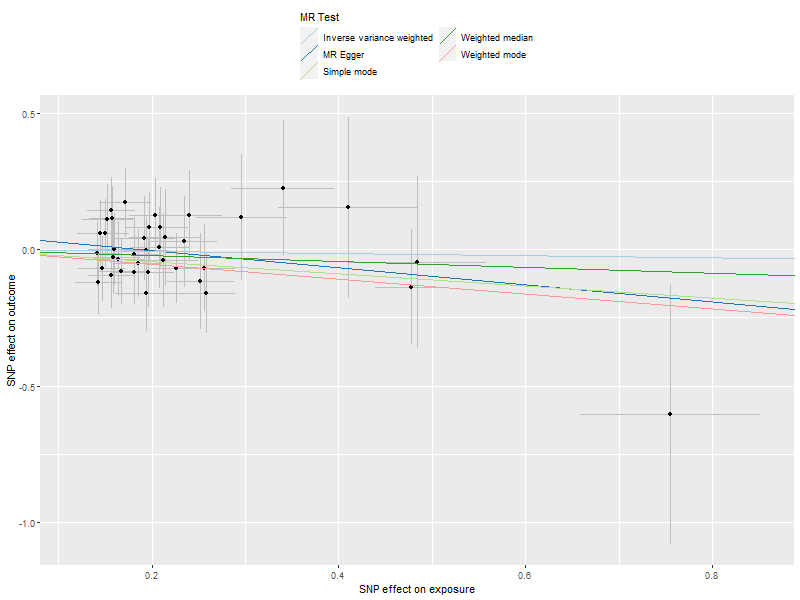


Fig S5. The scatter plot for the forward MR analysis of AD on HIC.


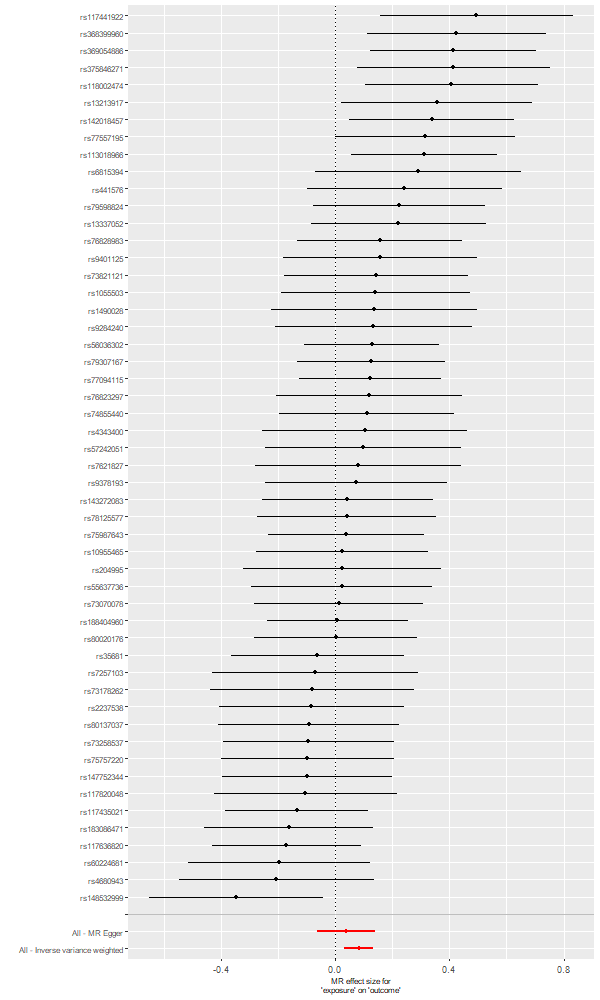


Fig S6. The forest plot of single snp for the forward MR analysis of AIH on HIC.


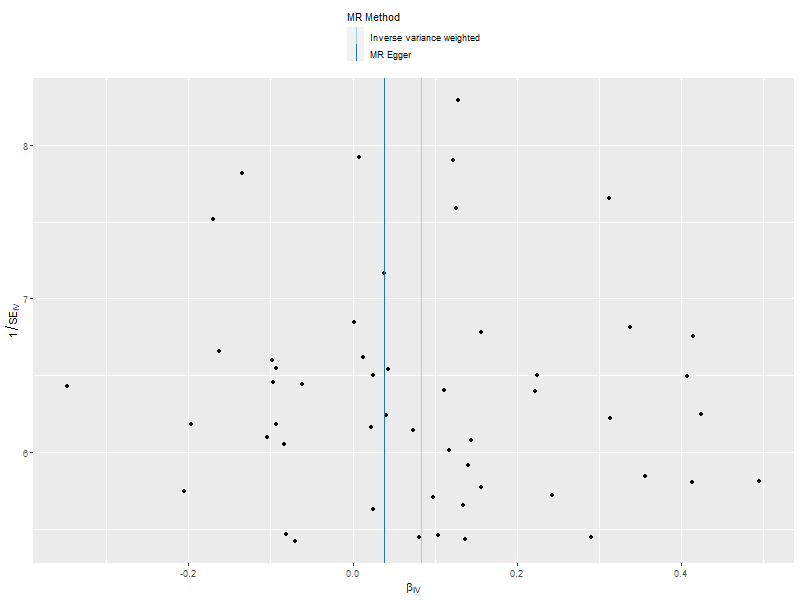


Fig S7. The funnel plot for the forward MR analysis of AIH on HIC.


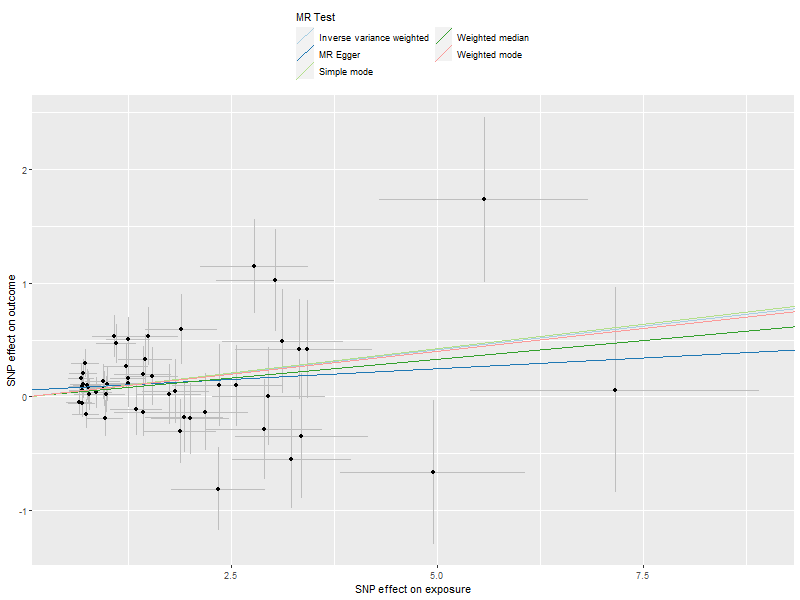


Fig S8. The scatter plot for the forward MR analysis of AIH on HIC.


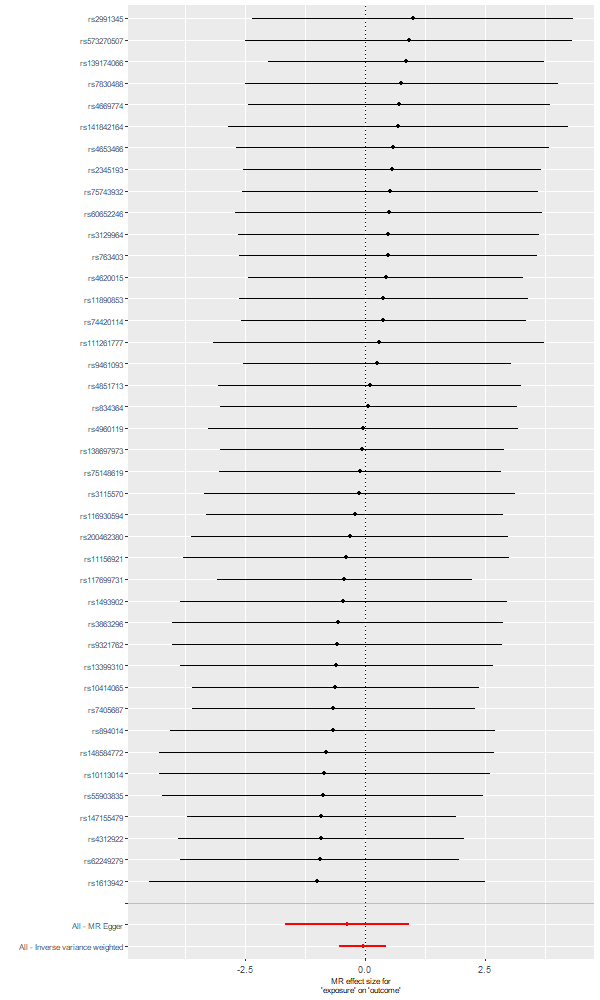


Fig S9. The forest plot of single snp for the forward MR analysis of AR on HIC.


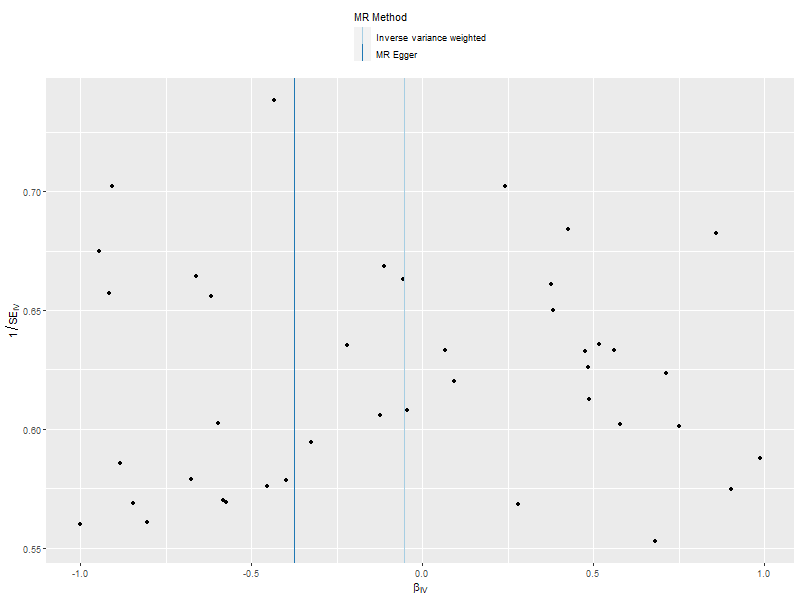


Fig S10. The funnel plot for the forward MR analysis of AR on HIC.


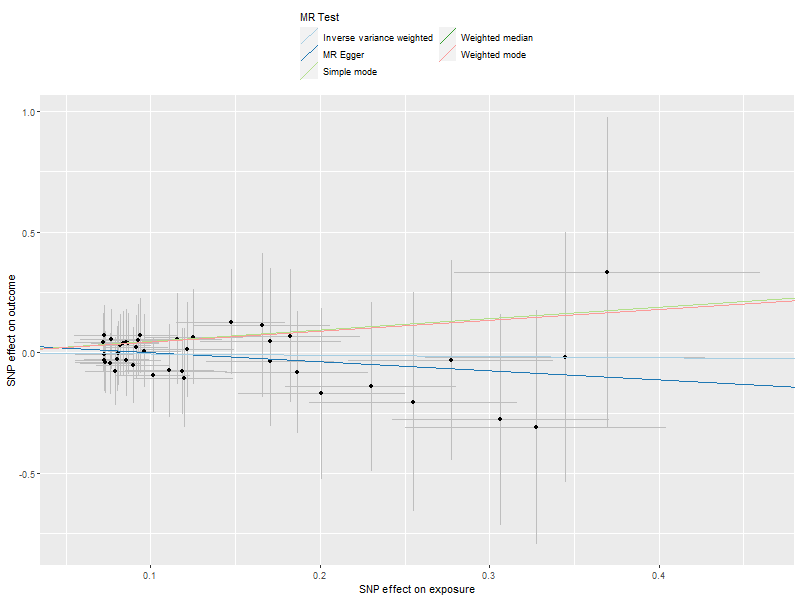


Fig S11. The scatter plot for the forward MR analysis of AR on HIC.


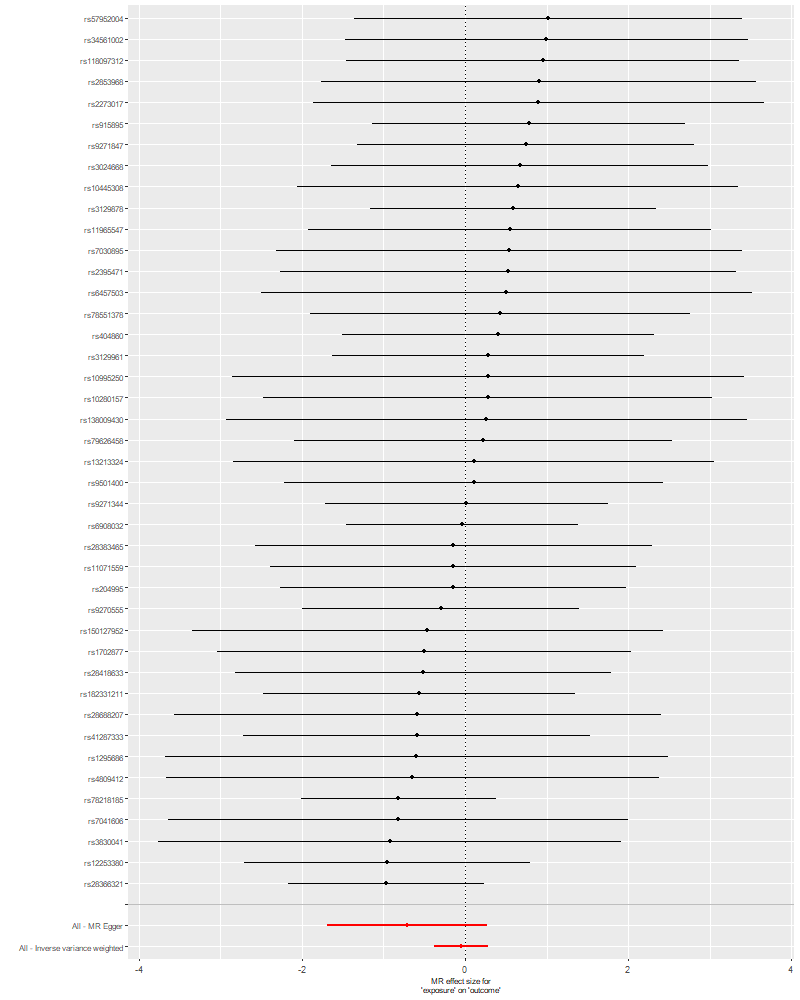


Fig S12. The forest plot of single snp for the forward MR analysis of AS on HIC.


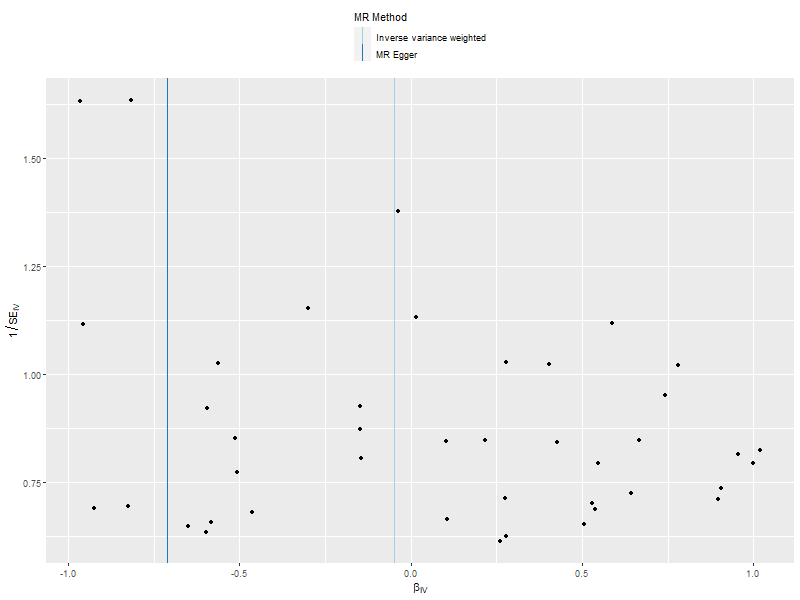


Fig S13. The funnel plot for the forward MR analysis of AS on HIC.


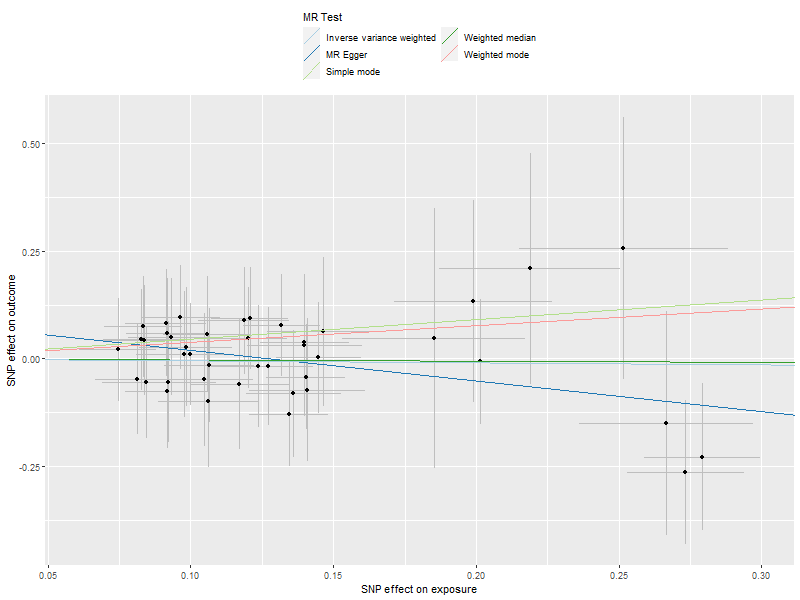


Fig S14. The scatter plot for the forward MR analysis of AS on HIC.


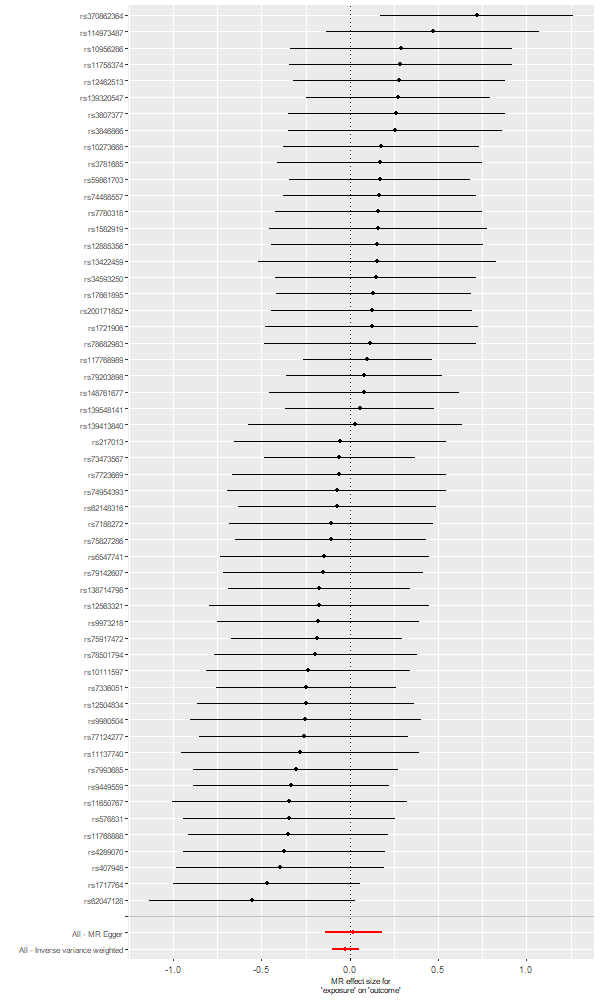


Fig S15. The forest plot of single snp for the forward MR analysis of CD on HIC.


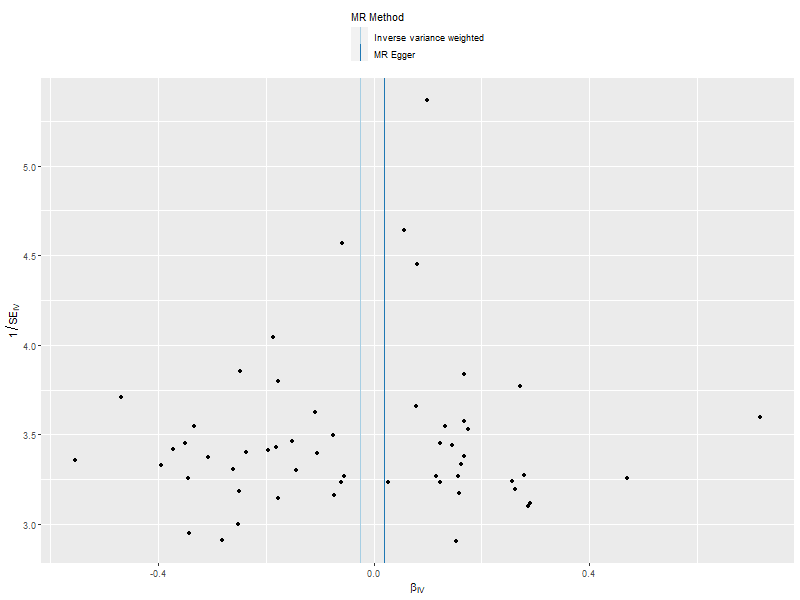


Fig S16. The funnel plot for the forward MR analysis of CD on HIC.


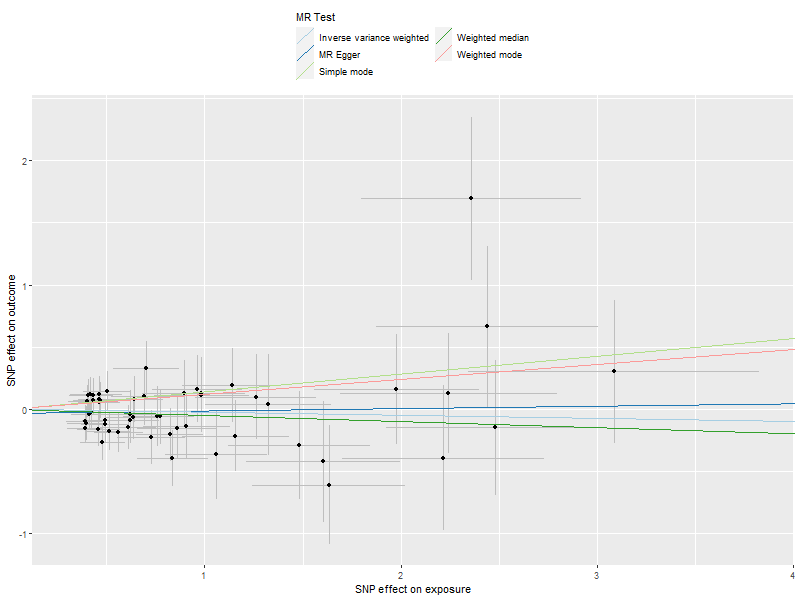


Fig S17. The scatter plot for the forward MR analysis of CD on HIC.


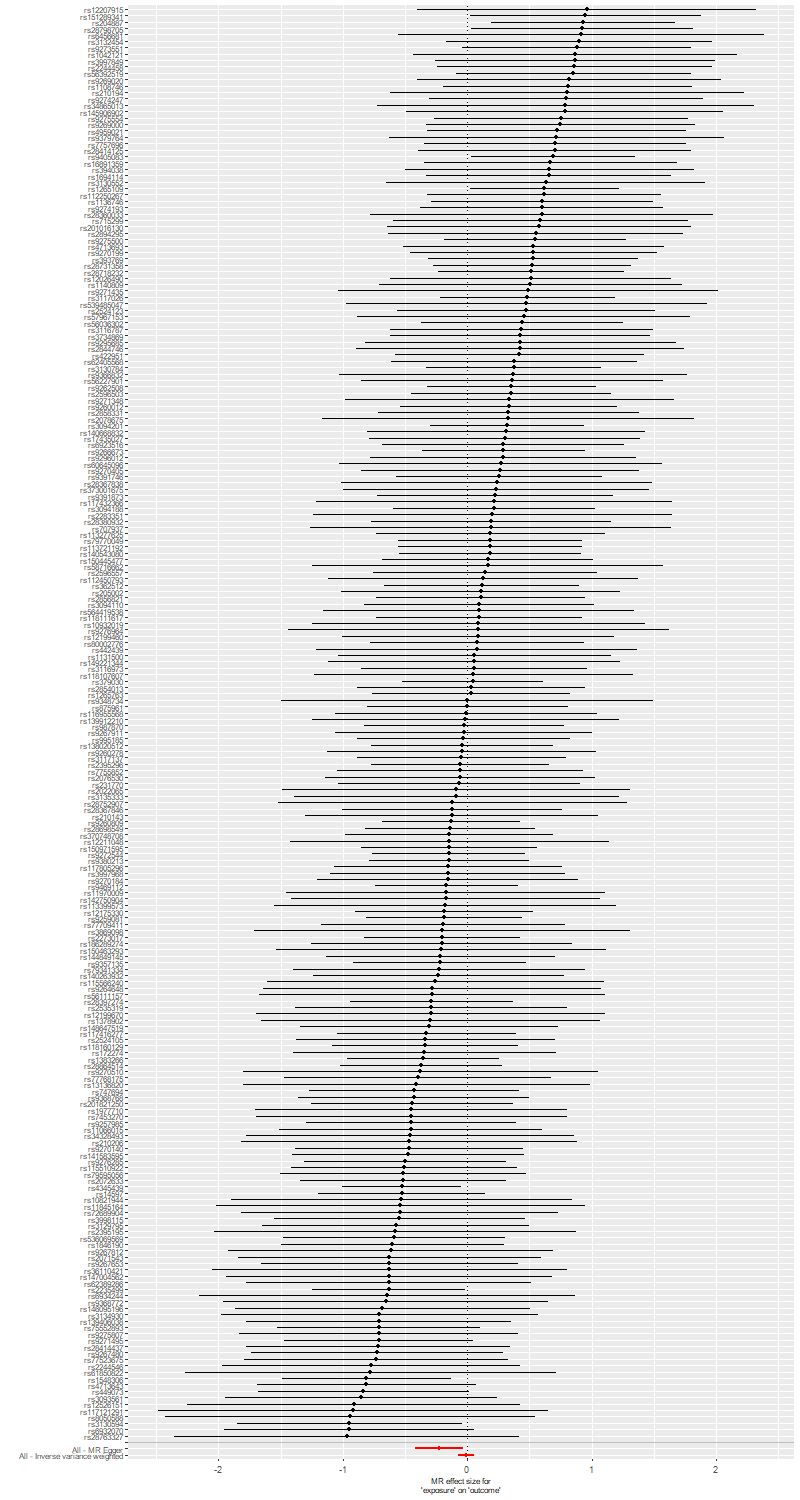


Fig S18. The forest plot of single snp for the forward MR analysis of GD on HIC.


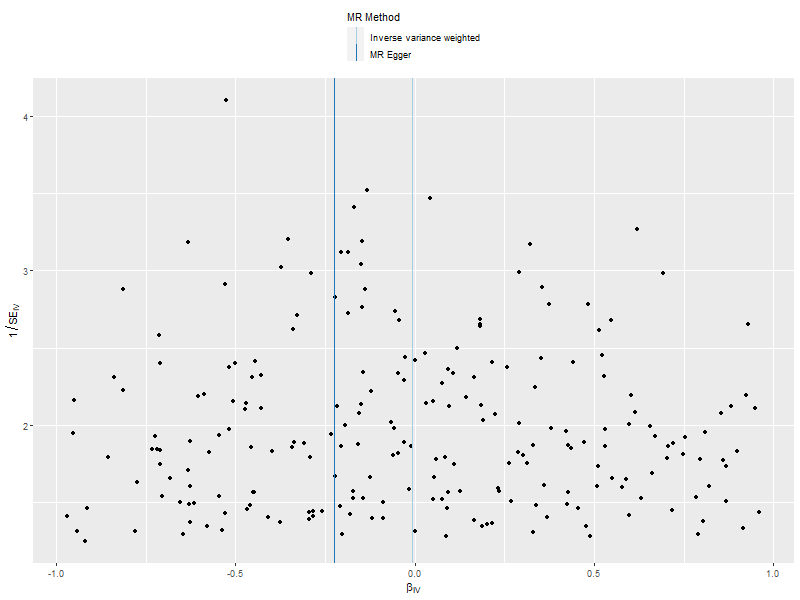


Fig S19. The funnel plot for the forward MR analysis of GD on HIC.


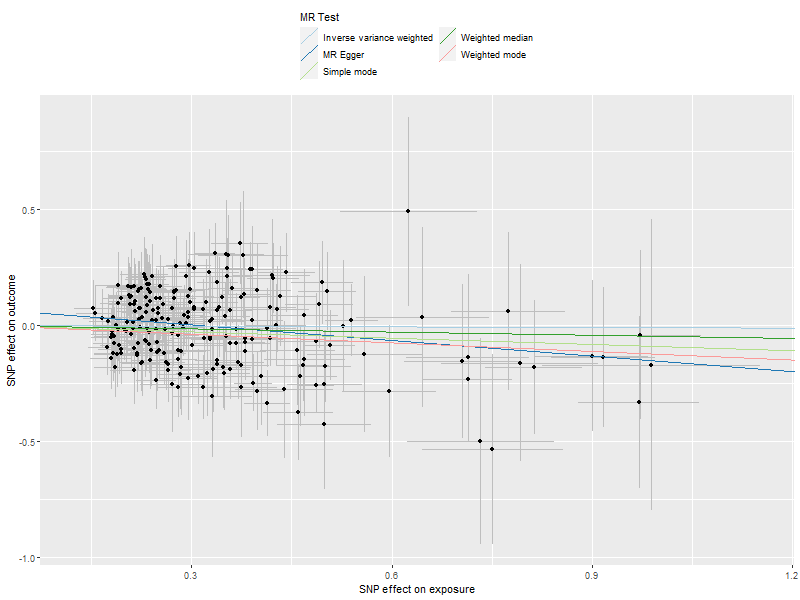


Fig S20. The scatter plot for the forward MR analysis of GD on HIC.


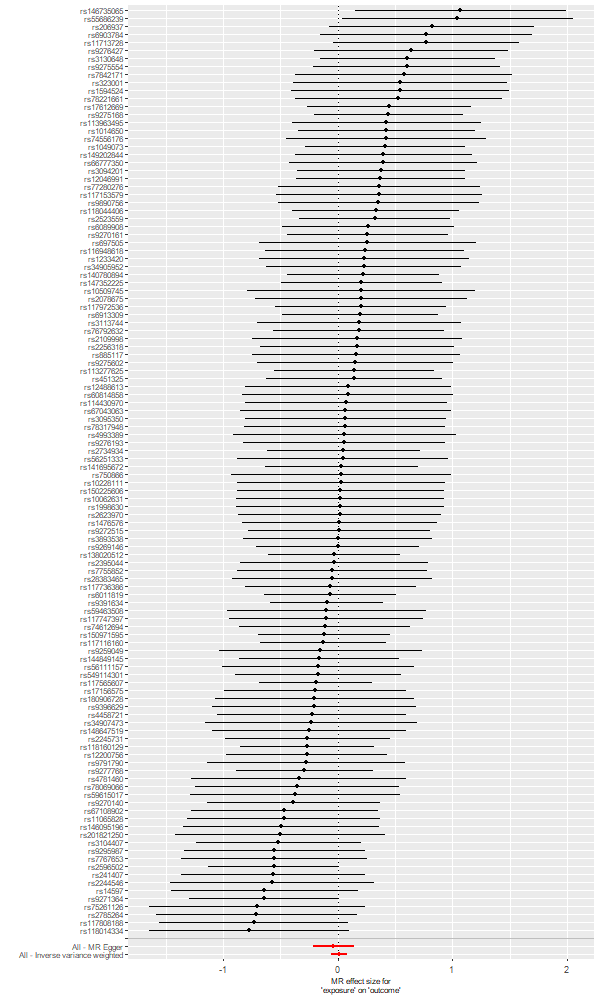


Fig S21. The forest plot of single snp for the forward MR analysis of HT on HIC.


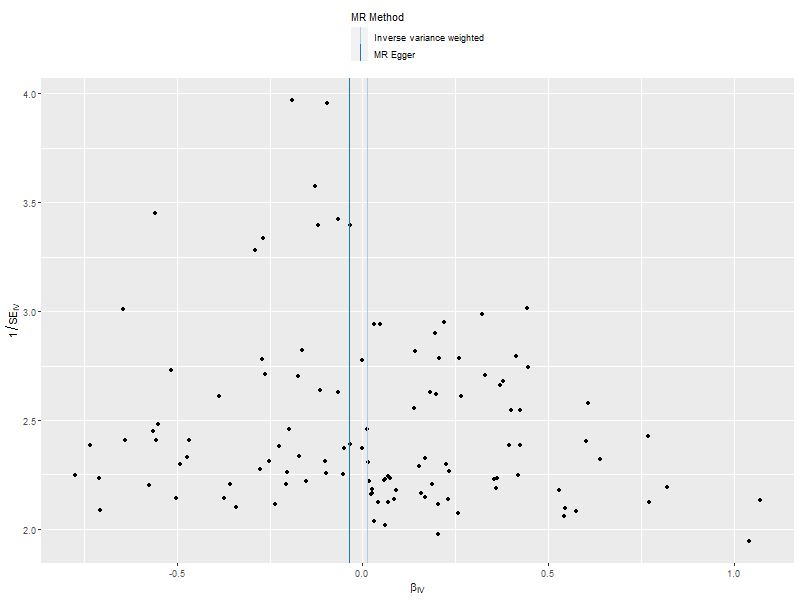


Fig S22. The funnel plot for the forward MR analysis of HT on HIC.


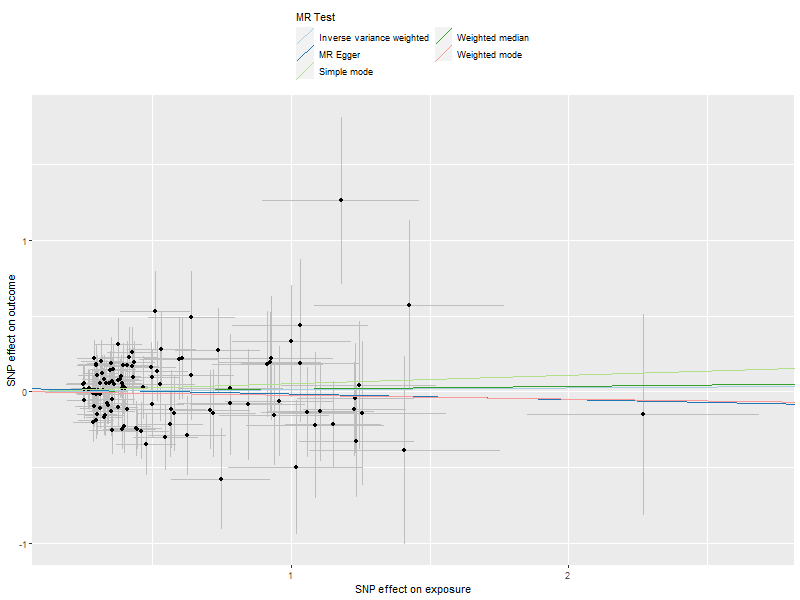


Fig S23. The scatter plot for the forward MR analysis of HT on HIC.


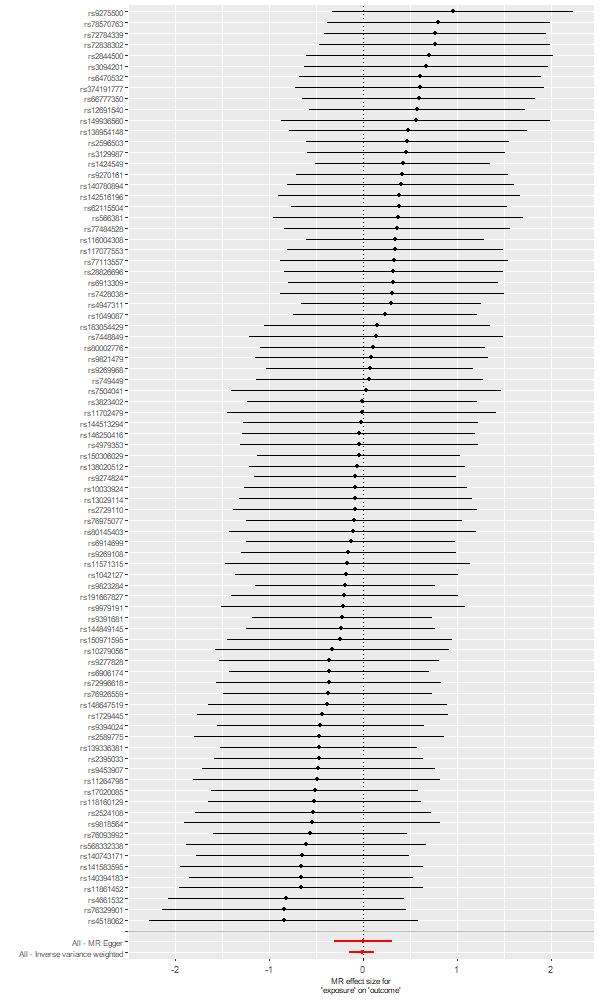


Fig S24. The forest plot of single snp for the forward MR analysis of HY on HIC.


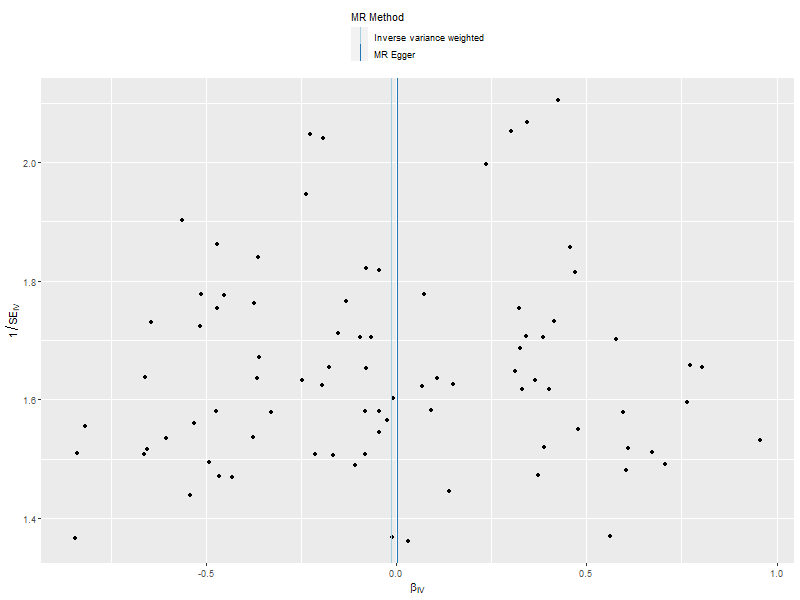


Fig S25. The funnel plot for the forward MR analysis of HY on HIC.


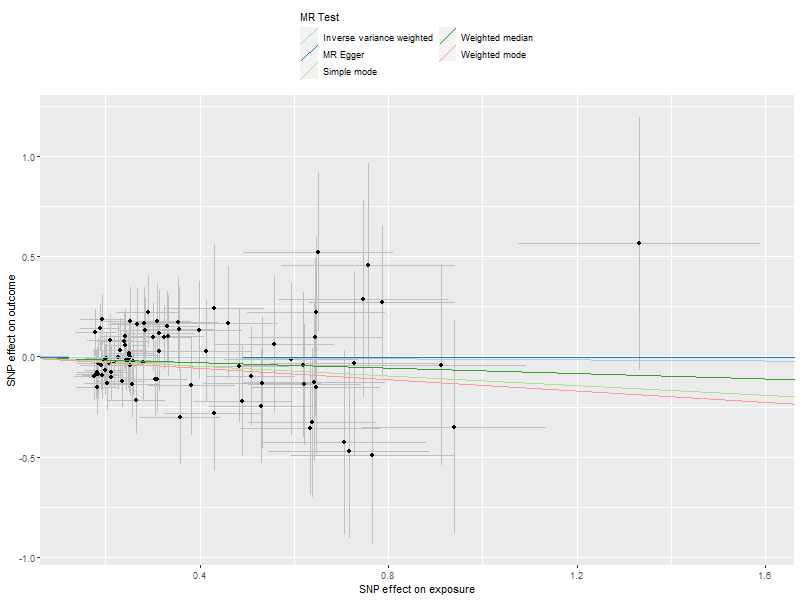


Fig S26. The scatter plot for the forward MR analysis of HY on HIC.


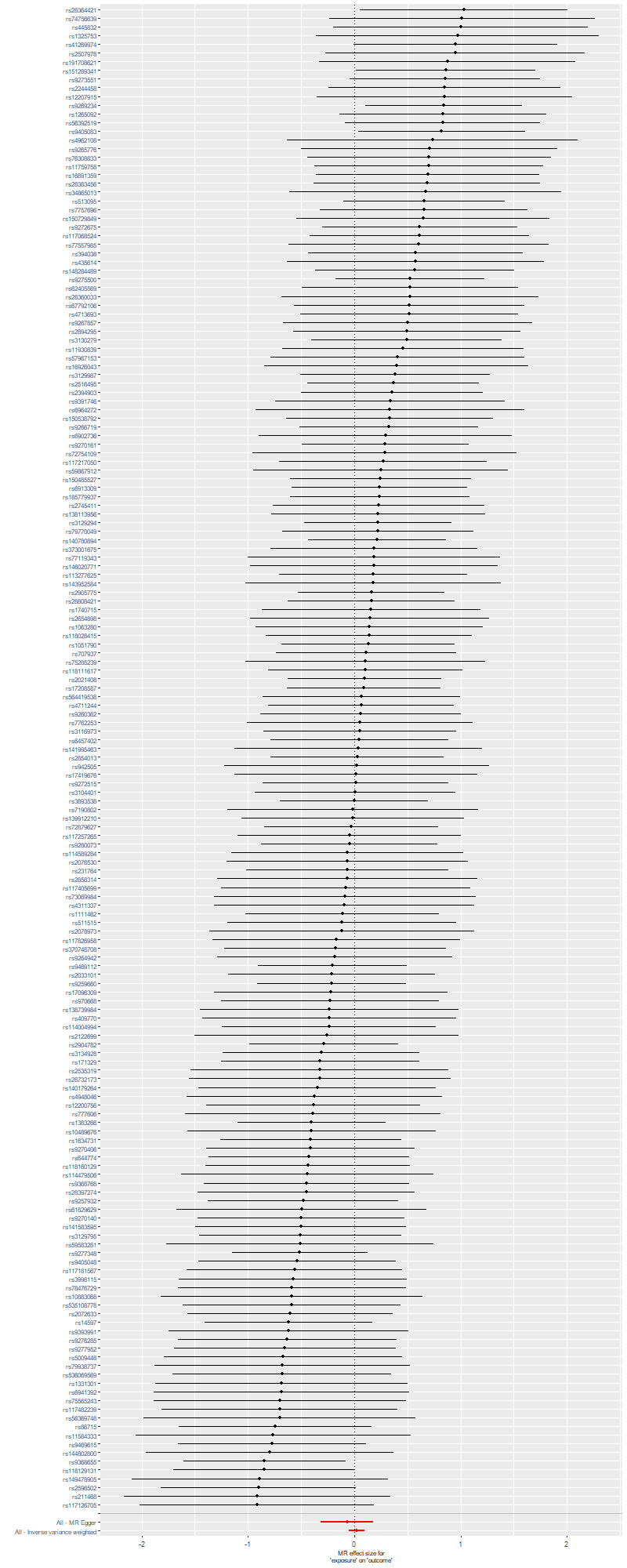


Fig S27. The forest plot of single snp for the forward MR analysis of HYPE on HIC.


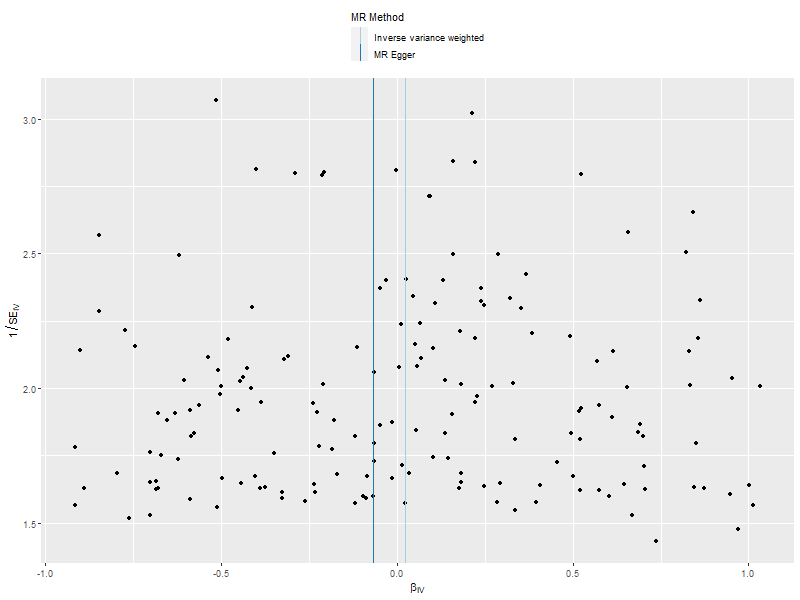


Fig S28. The funnel plot of single snp for the forward MR analysis of HYPE on HIC.


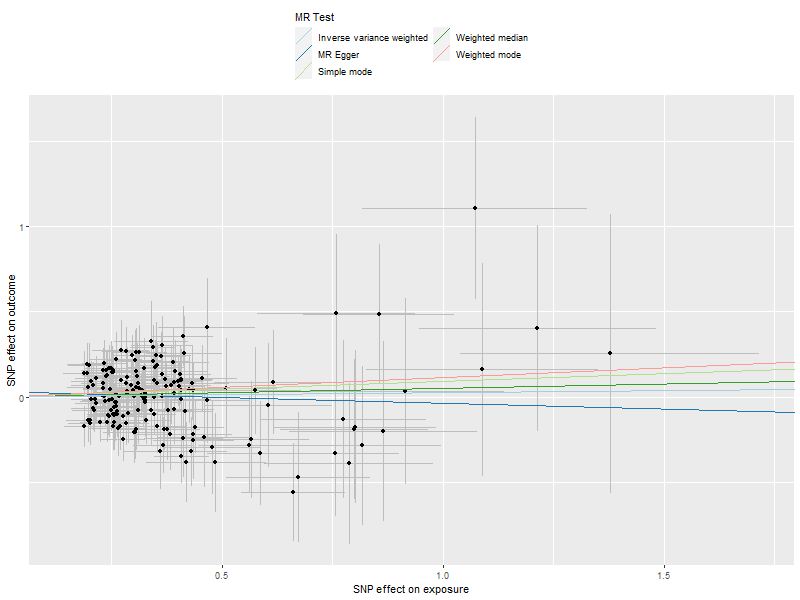


Fig S29. The scatter plot of single snp for the forward MR analysis of HYPE on HIC.


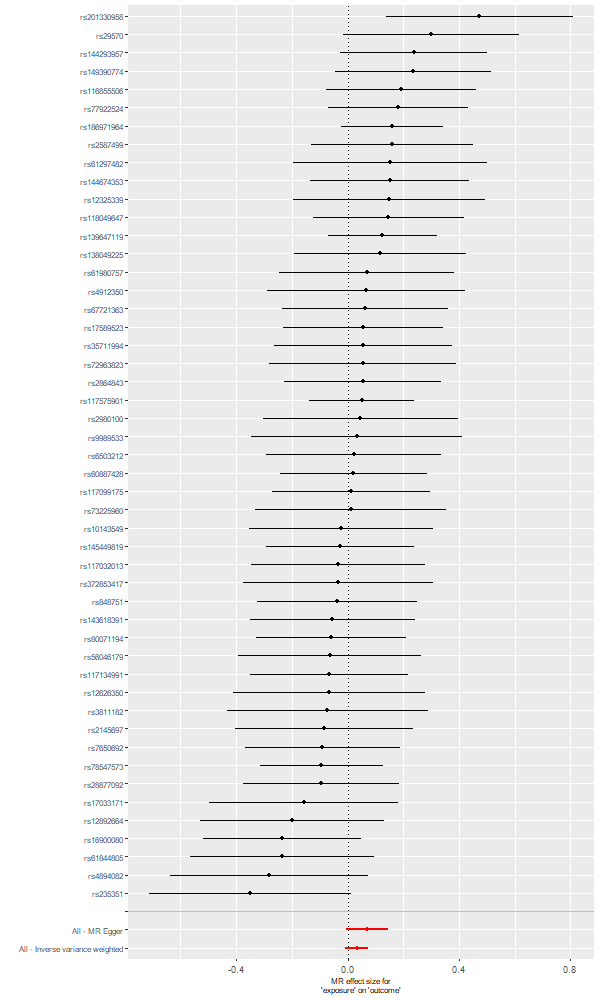


Fig S30. The forest plot of single snp for the forward MR analysis of MG on HIC.


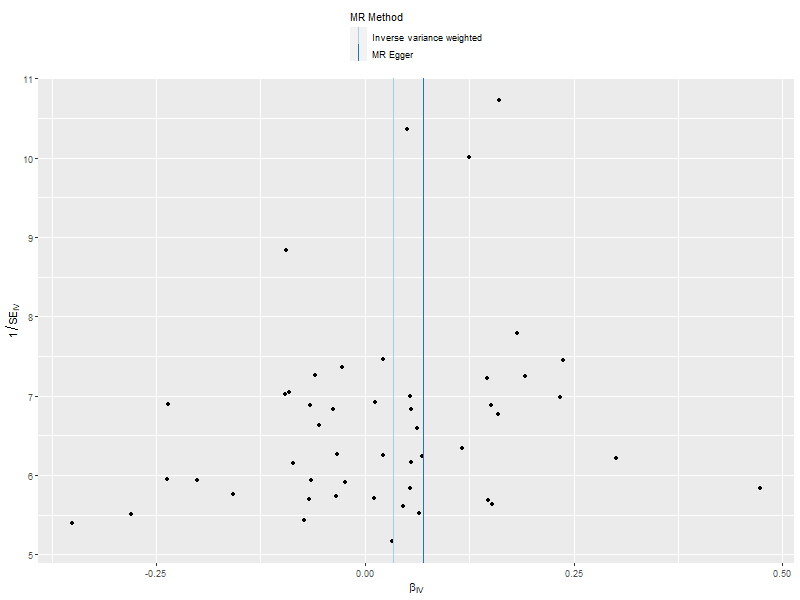


Fig S31. The funnel plot for the forward MR analysis of MG on HIC.


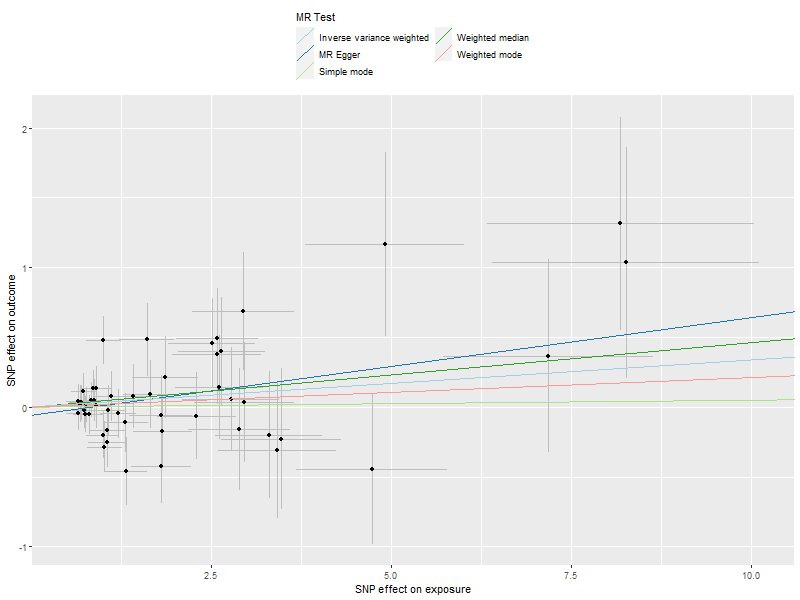


Fig S32. The scatter plot for the forward MR analysis of MG on HIC.


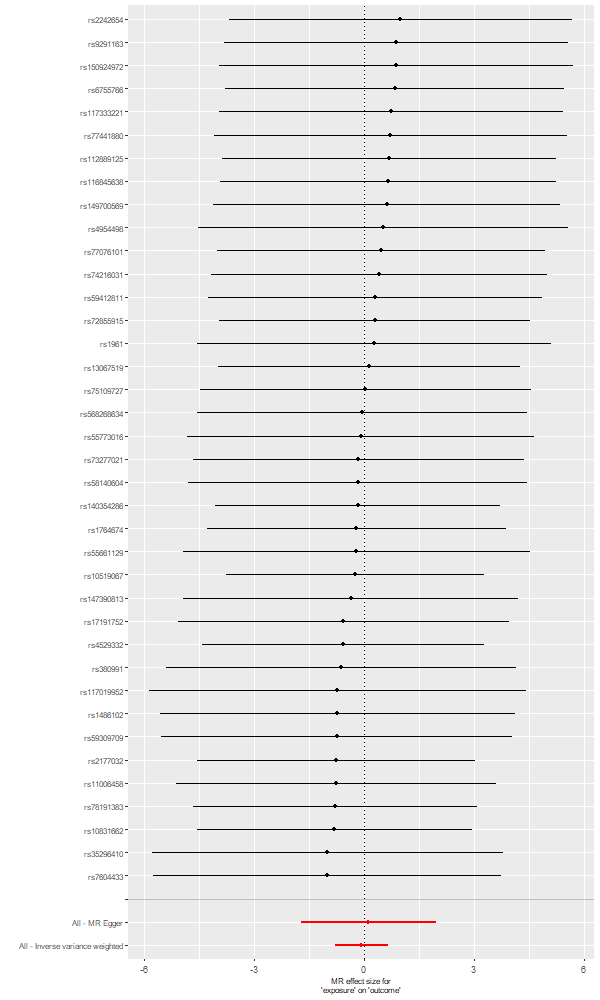


Fig S33. The forest plot of single snp for the forward MR analysis of PO on HIC.


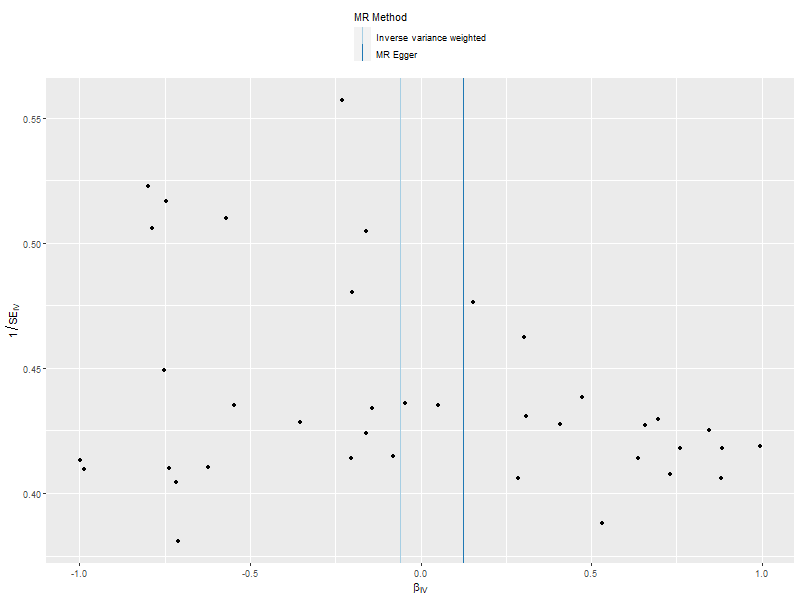


Fig S34. The funnel plot for the forward MR analysis of PO on HIC.


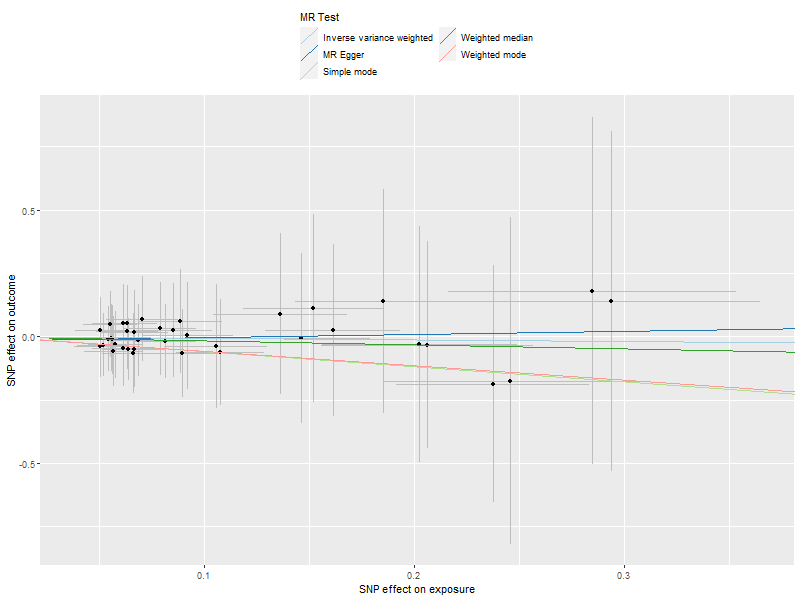


Fig S35. The scatter plot of single snp for the forward MR analysis of PO on HIC.


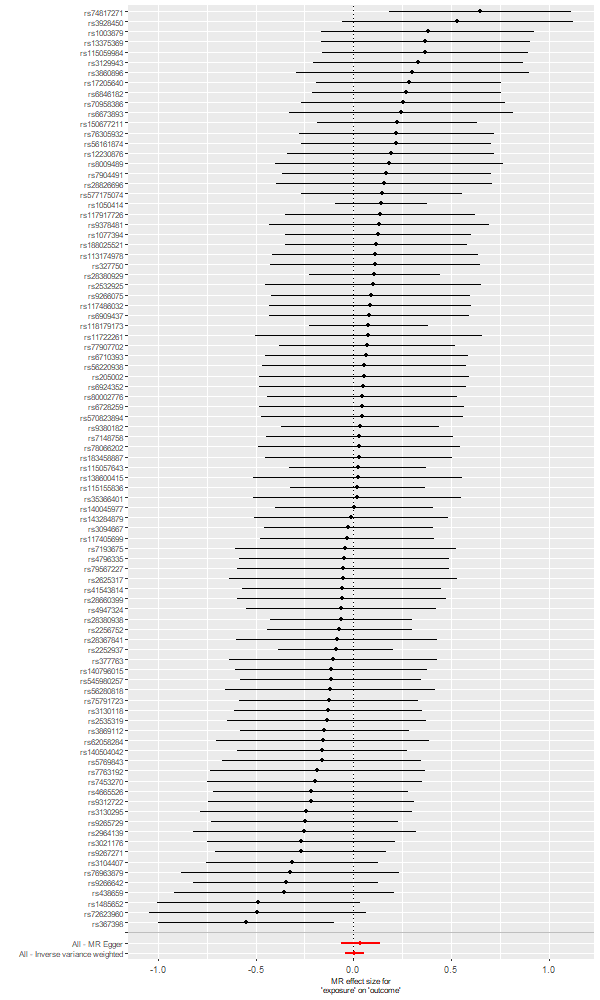


Fig S36. The forest plot of single snp for the forward MR analysis of PV on HIC.


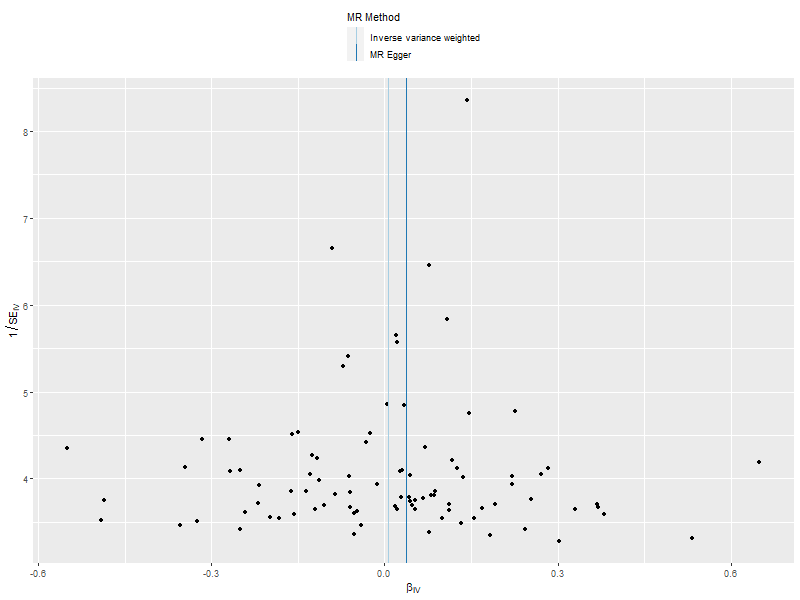


Fig S37. The funnel plot for the forward MR analysis of PV on HIC.


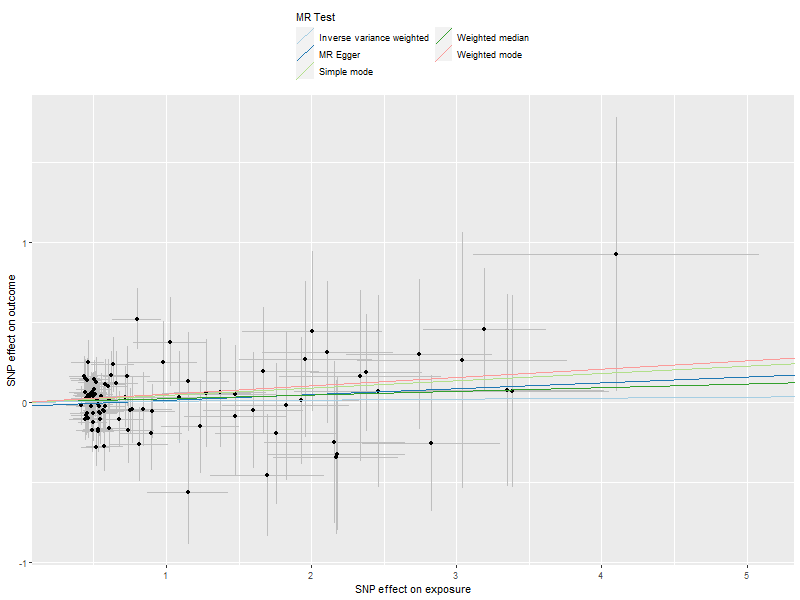


Fig S38. The scatter plot for the forward MR analysis of PV on HIC.


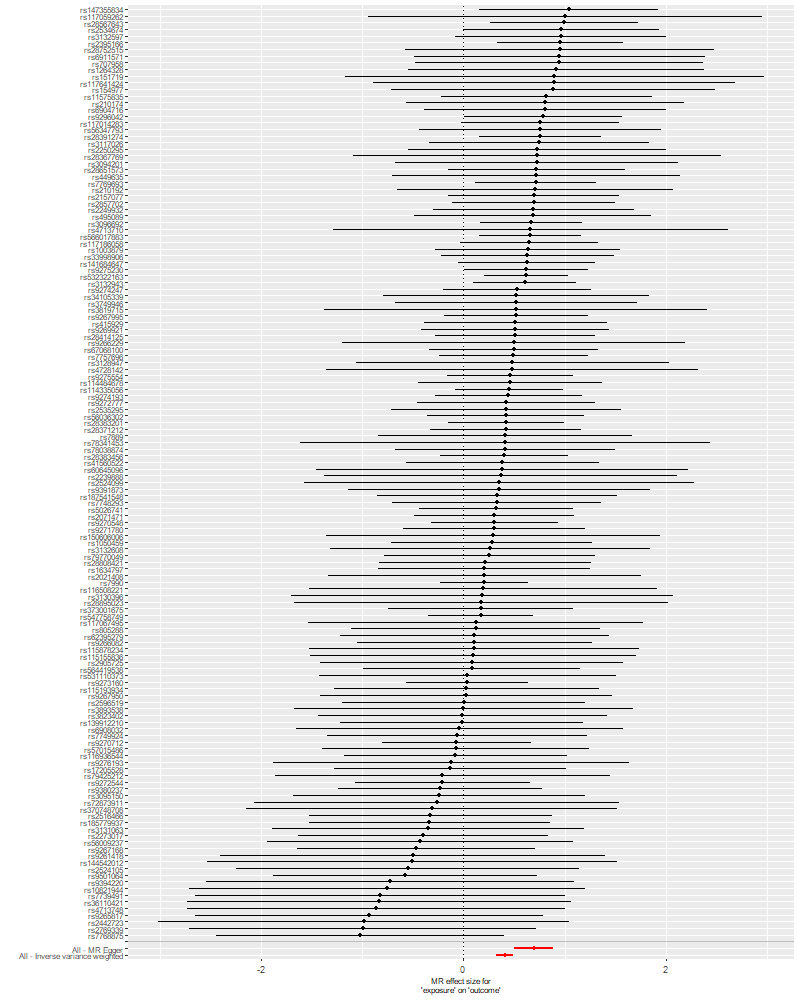


Fig S39. The forest plot of single snp for the forward MR analysis of RA on HIC.


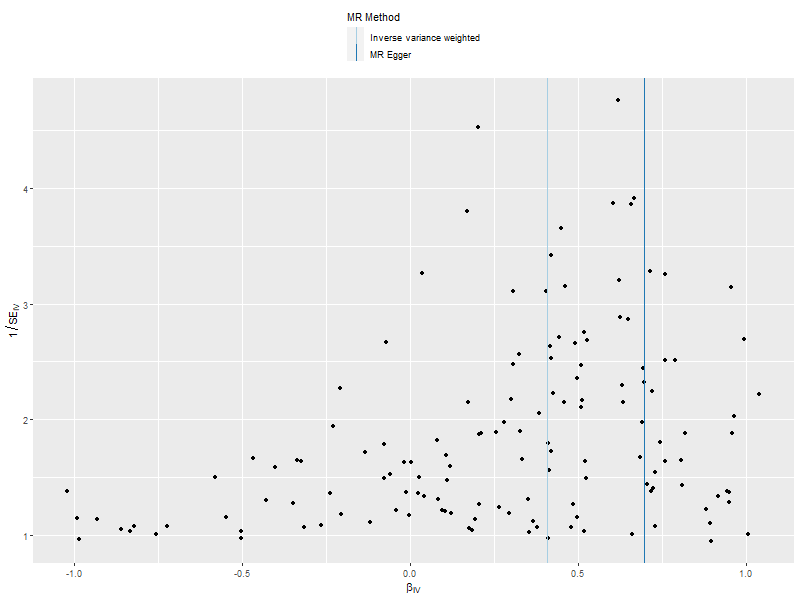


Fig S40. The funnel plot for the forward MR analysis of RA on HIC.


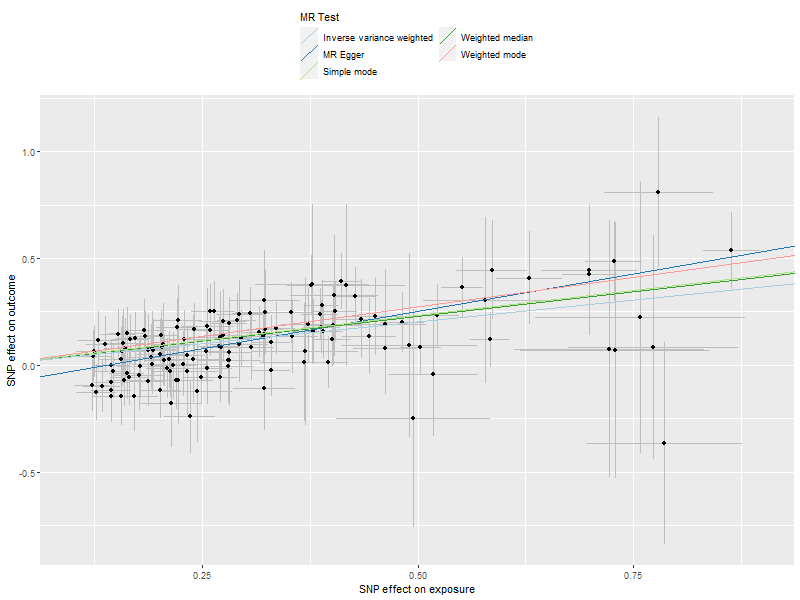


Fig S41. The scatter plot for the forward MR analysis of RA on HIC.


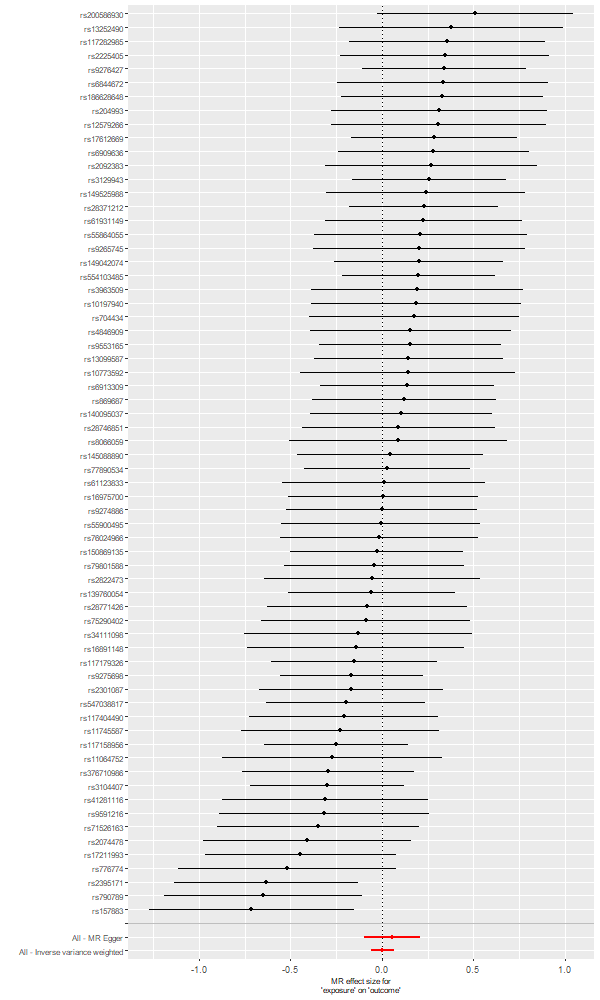


Fig S42. The forest plot of single snp for the forward MR analysis of SA on HIC.


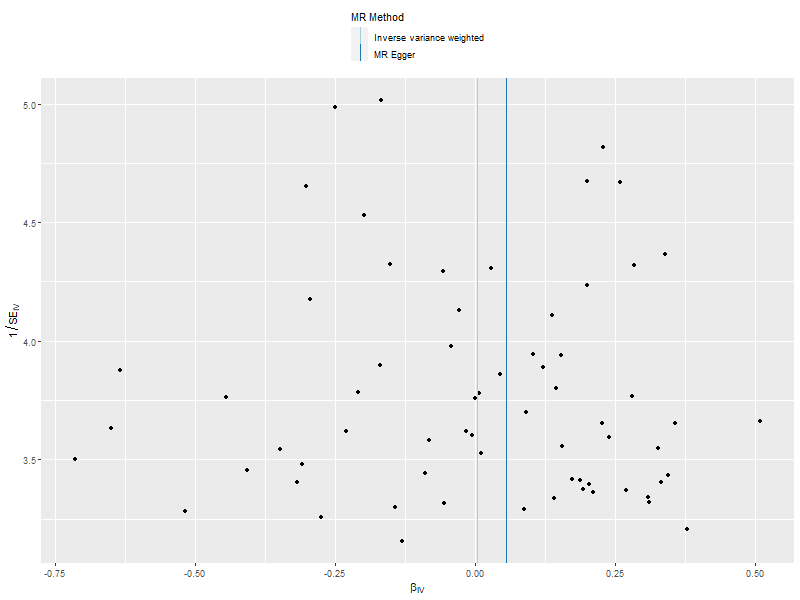


Fig S43. The funnel plot for the forward MR analysis of SA on HIC.


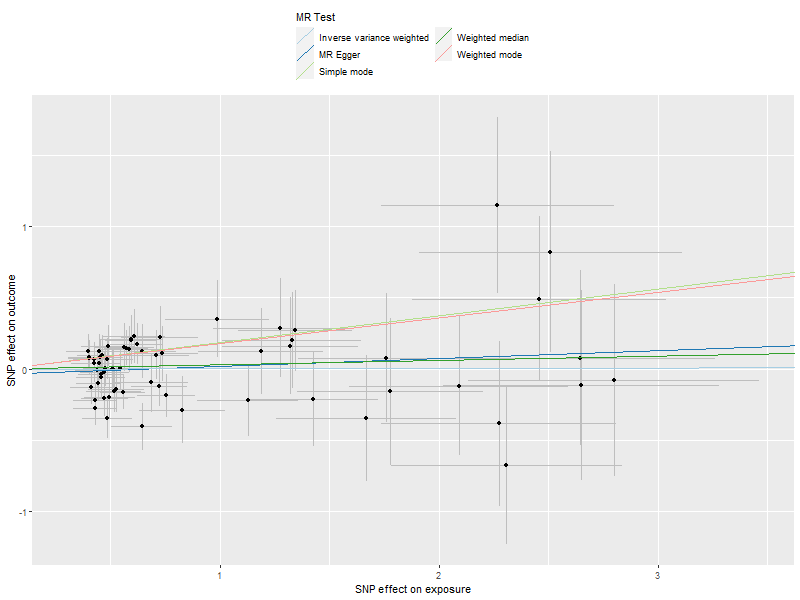


Fig S44. The scatter plot for the forward MR analysis of SA on HIC.


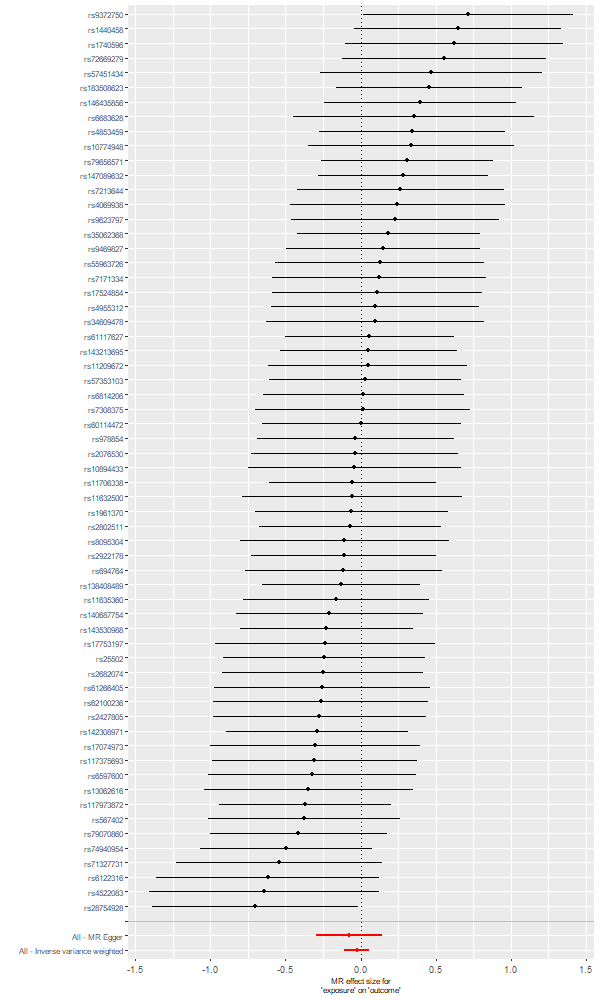


Fig S45. The forest plot of single snp for the forward MR analysis of SLE on HIC.


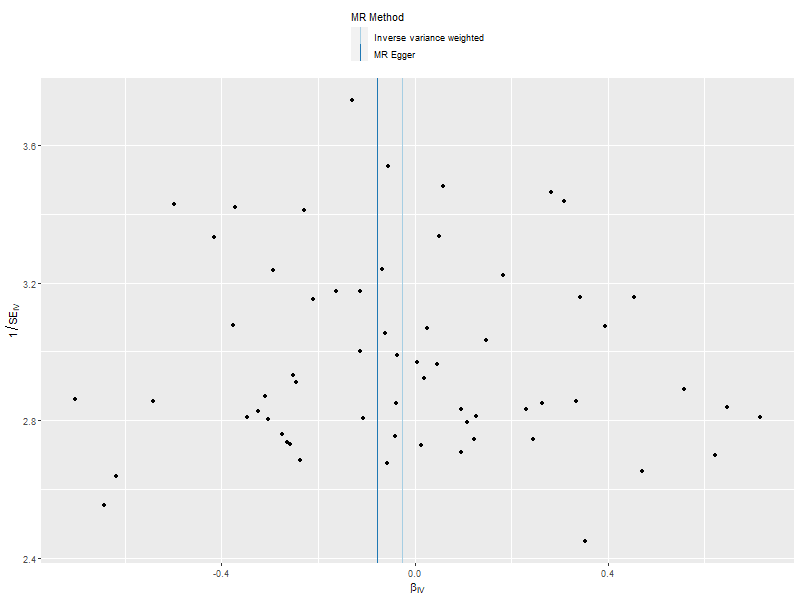


Fig S46. The funnel plot for the forward MR analysis of SLE on HIC.


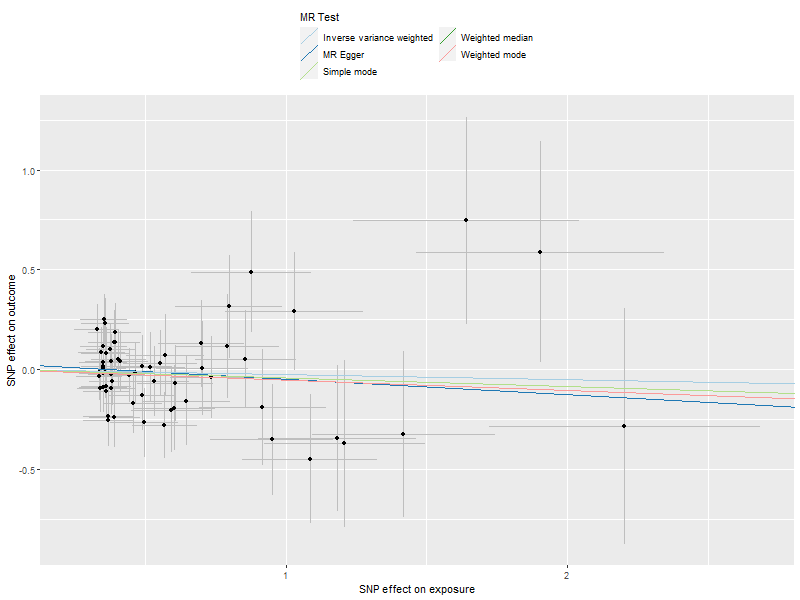


Fig S47. The scatter plot for the forward MR analysis of SLE on HIC.


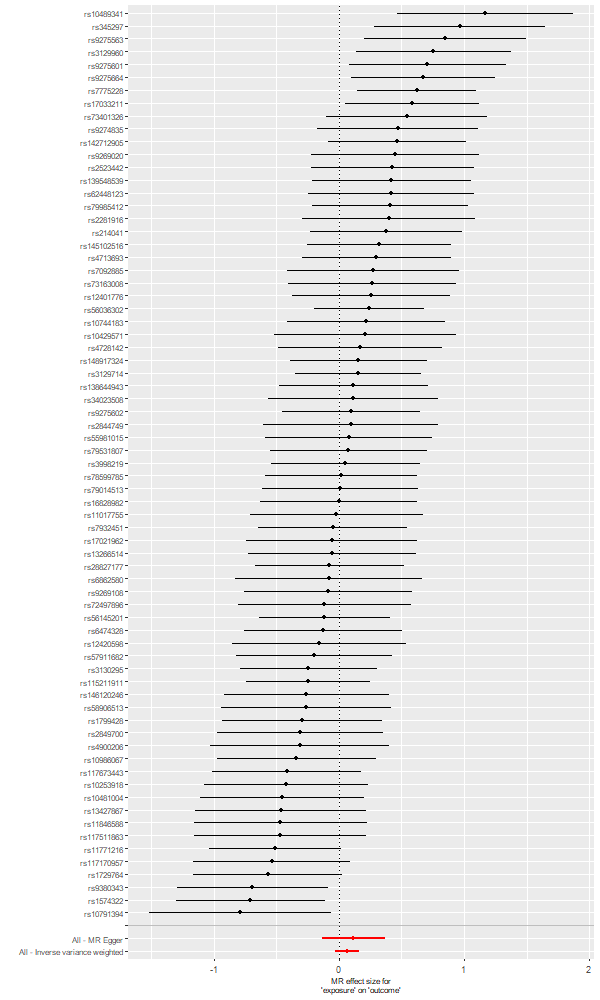


Fig S48. The forest plot of single snp for the forward MR analysis of SS on HIC.


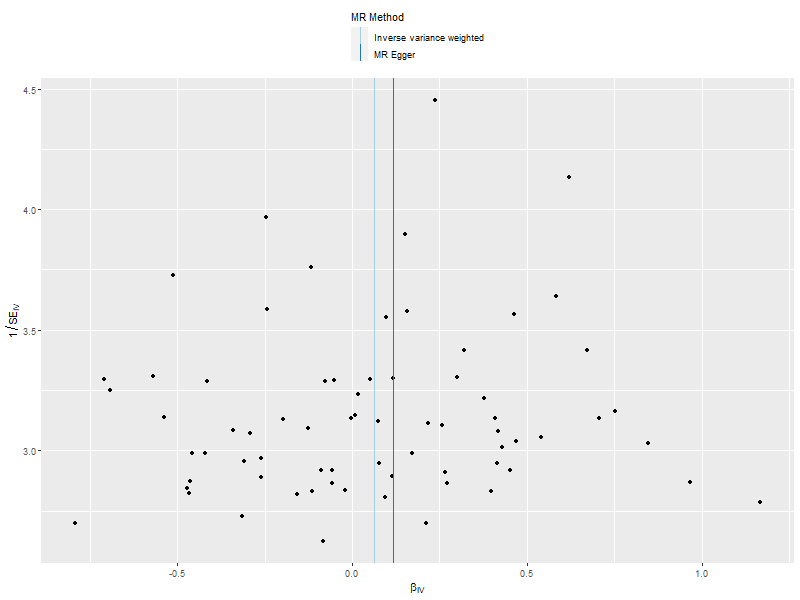


Fig S49. The funnel plot for the forward MR analysis of SS on HIC.


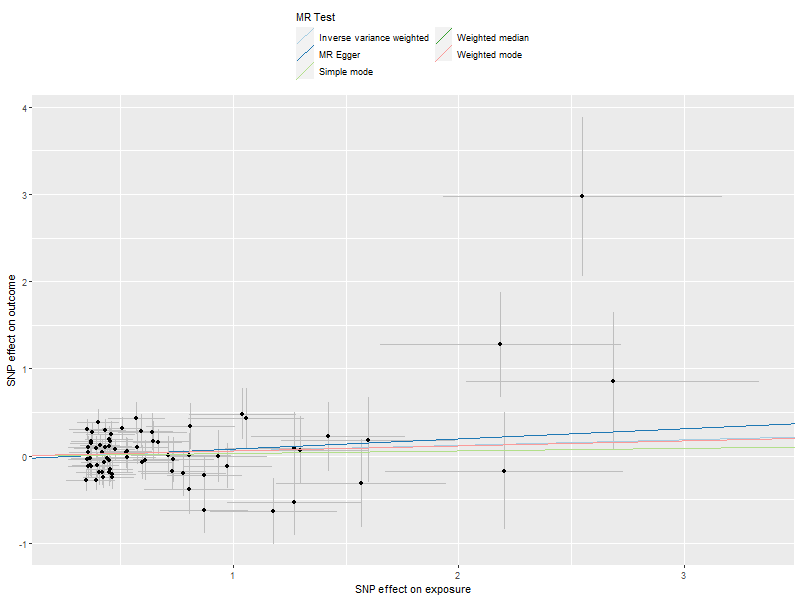


Fig S50. The scatter plot for the forward MR analysis of SS on HIC.


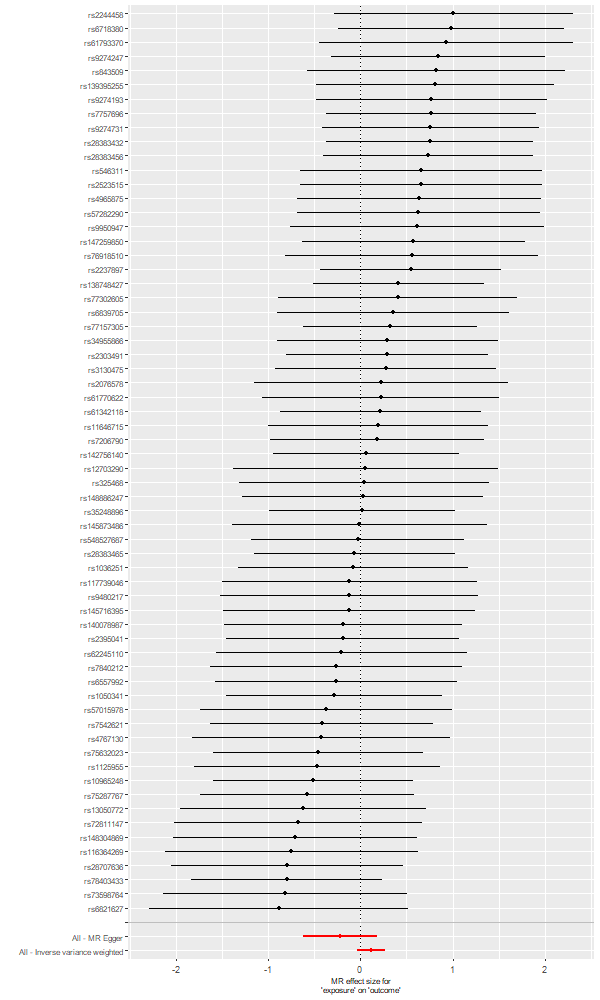


Fig S51. The forest plot of single snp for the forward MR analysis of T1D on HIC.


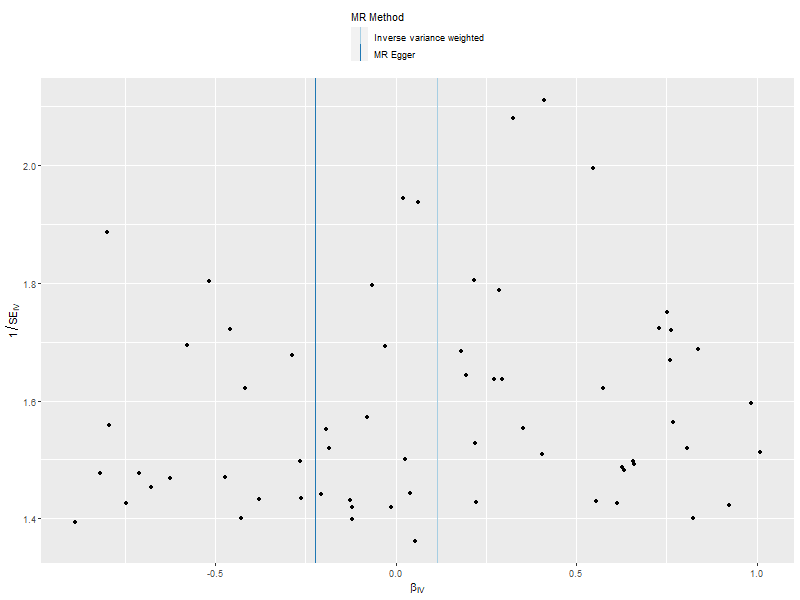


Fig S52. The funnel plot for the forward MR analysis of T1D on HIC.


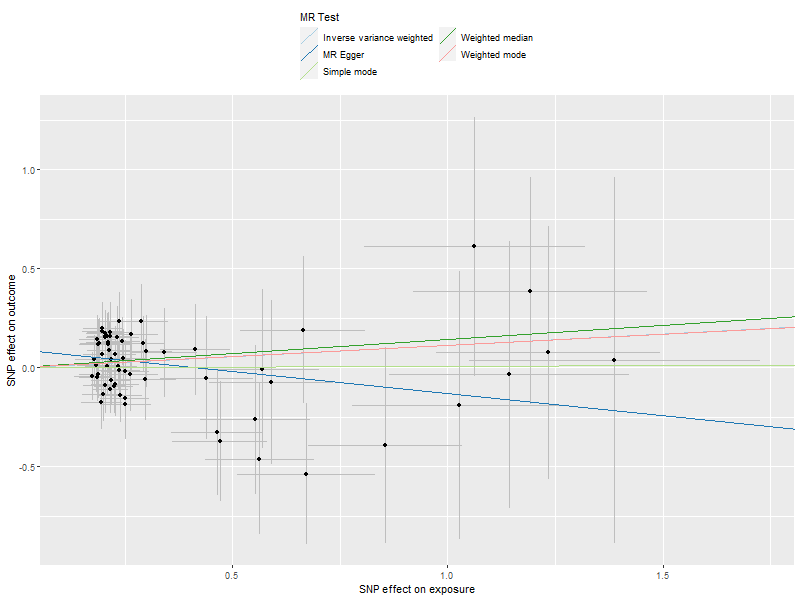


Fig S53. The scatter plot of single snp for the forward MR analysis of T1D on HIC.


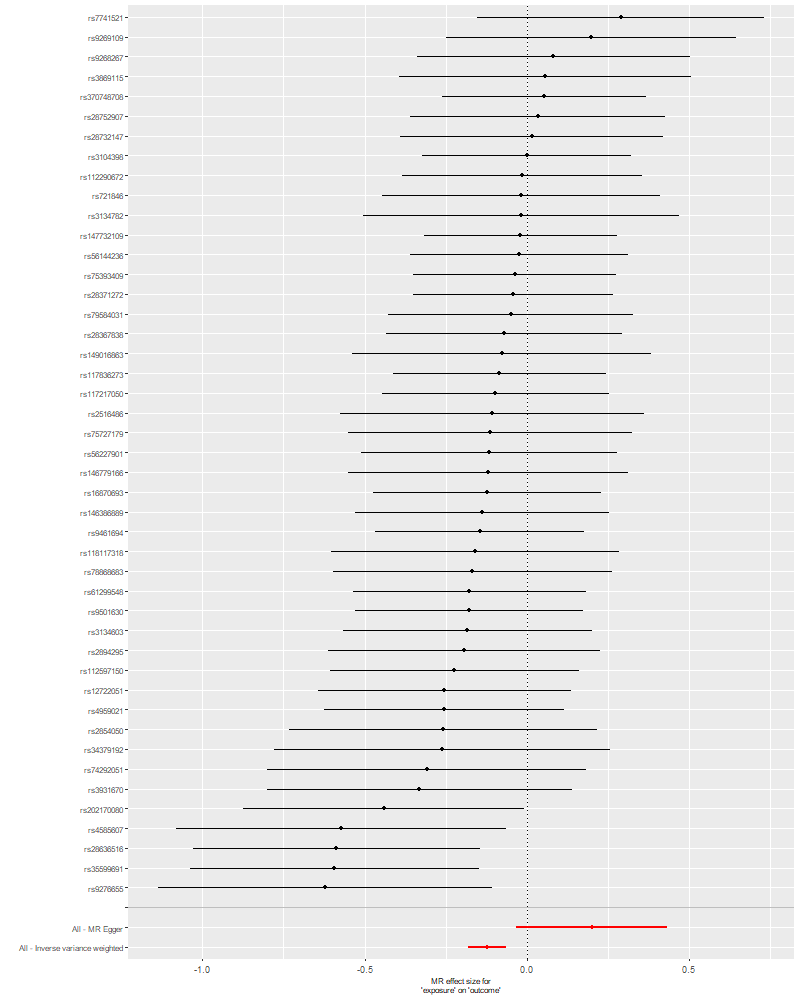


Fig S54. The forest plot of single snp for the forward MR analysis of UC on HIC.


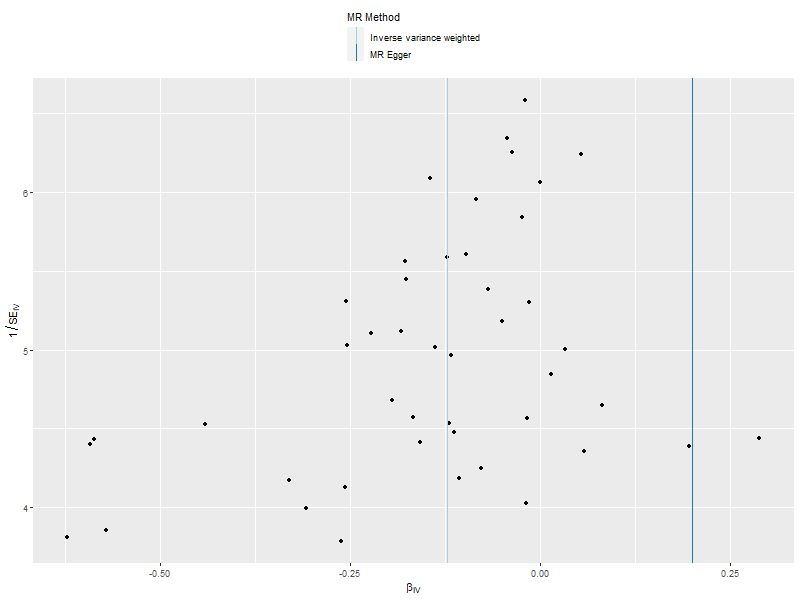


Fig S55. The funnel plot of single snp for the forward MR analysis of UC on HIC.


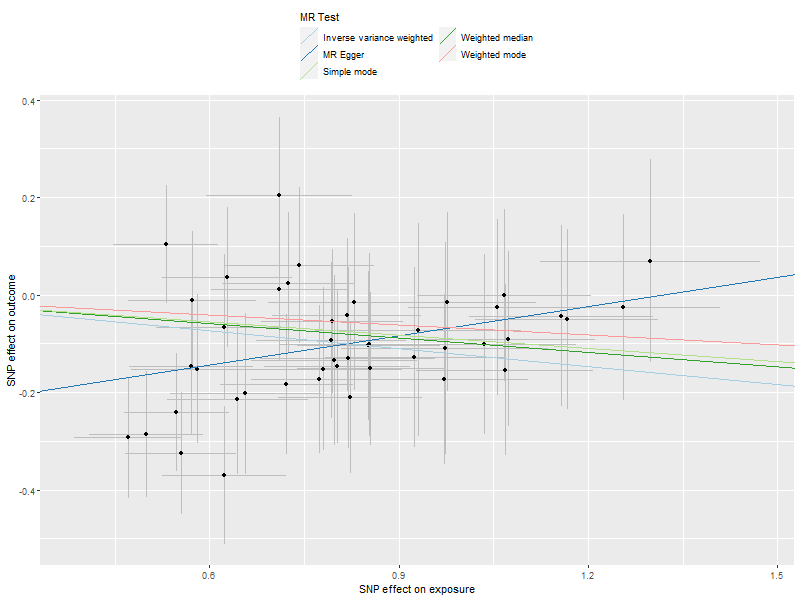


Fig S56. The scatter plot of single snp for the forward MR analysis of UC on HIC.


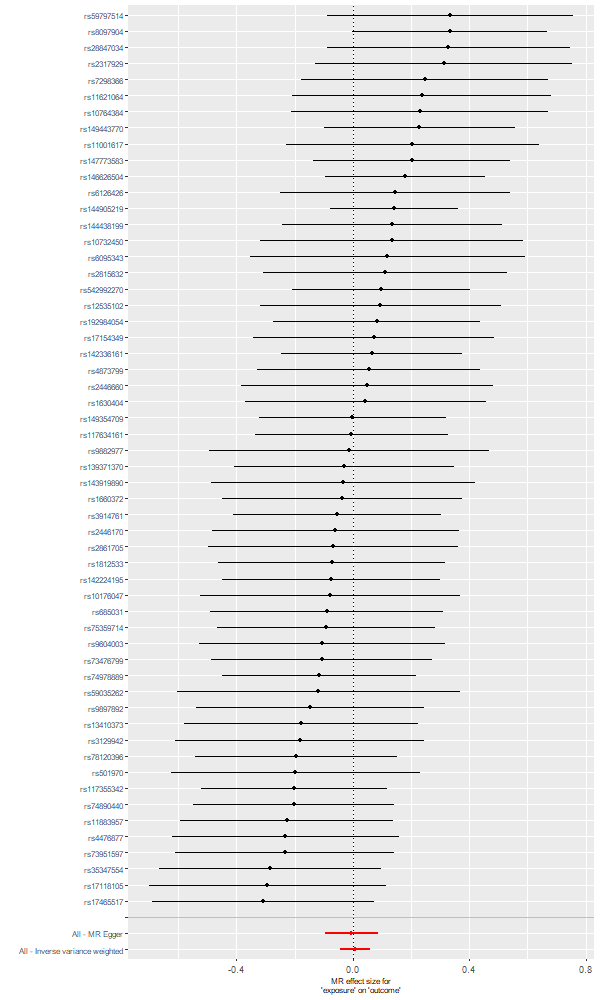


Fig S57. The forest plot of single snp for the forward MR analysis of UV on HIC.


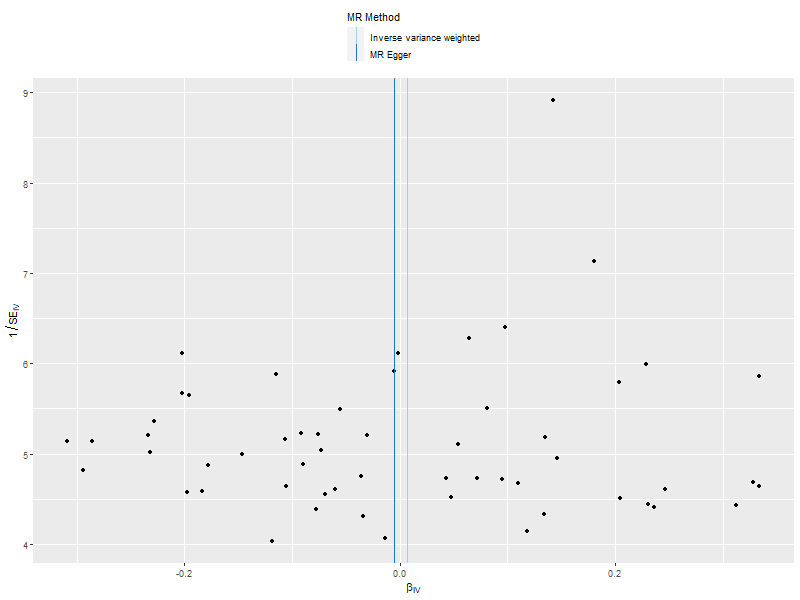


Fig S58. The funnel plot for the forward MR analysis of UV on HIC.


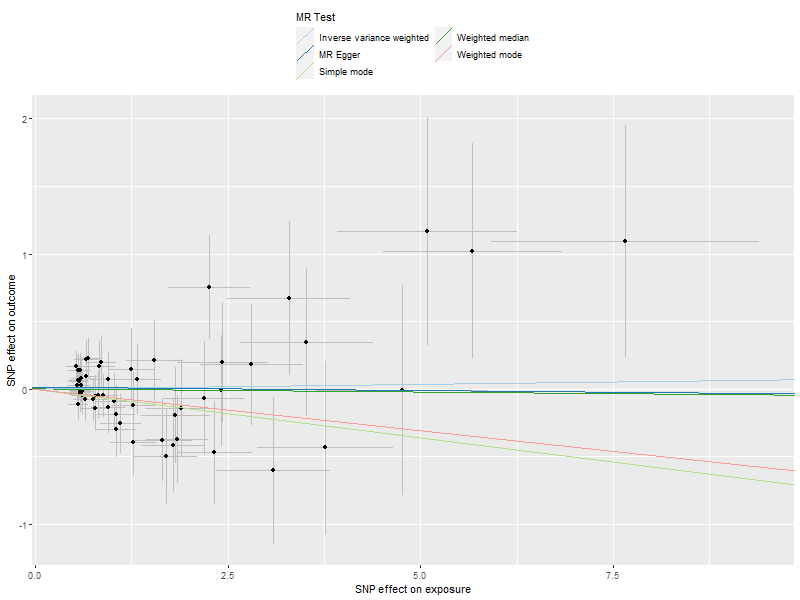


Fig S59. The scatter plot for the forward MR analysis of UV on HIC.


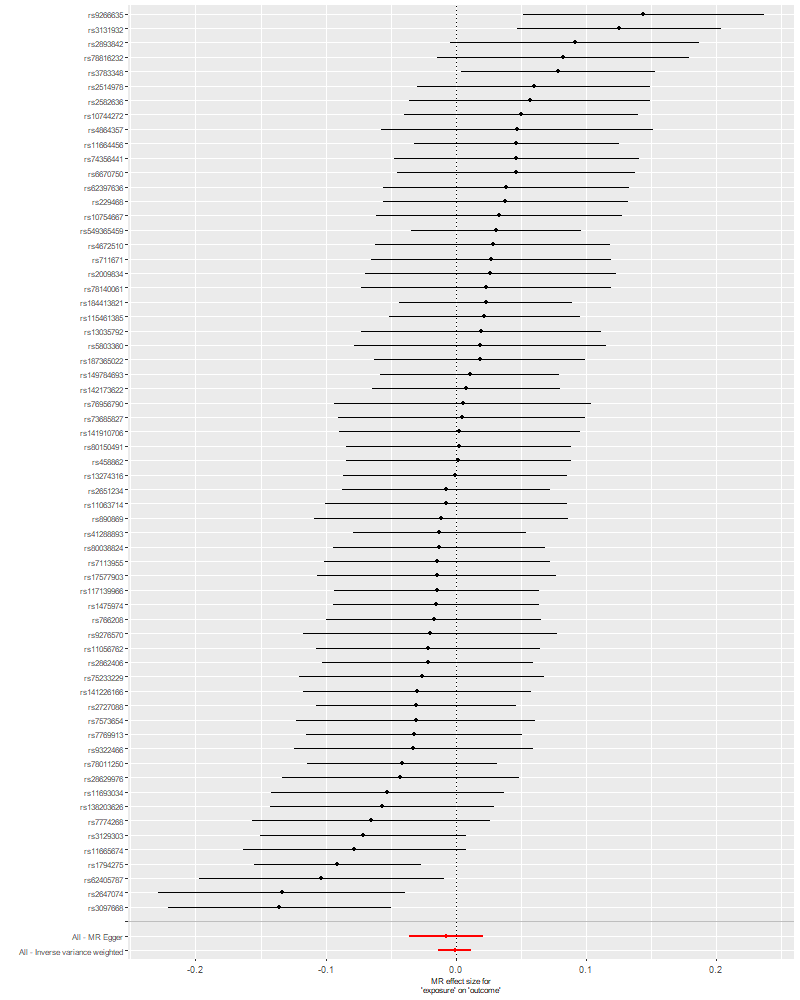


Fig S60. The forest plot of single snp for the reverse MR analysis of HIC on AD.


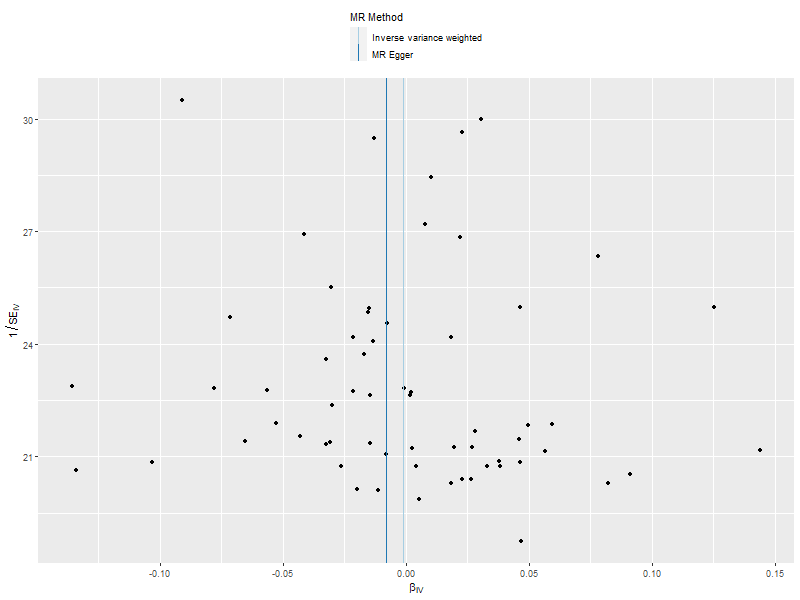


Fig S61. The funnel plot for the reverse MR analysis of HIC on AD.


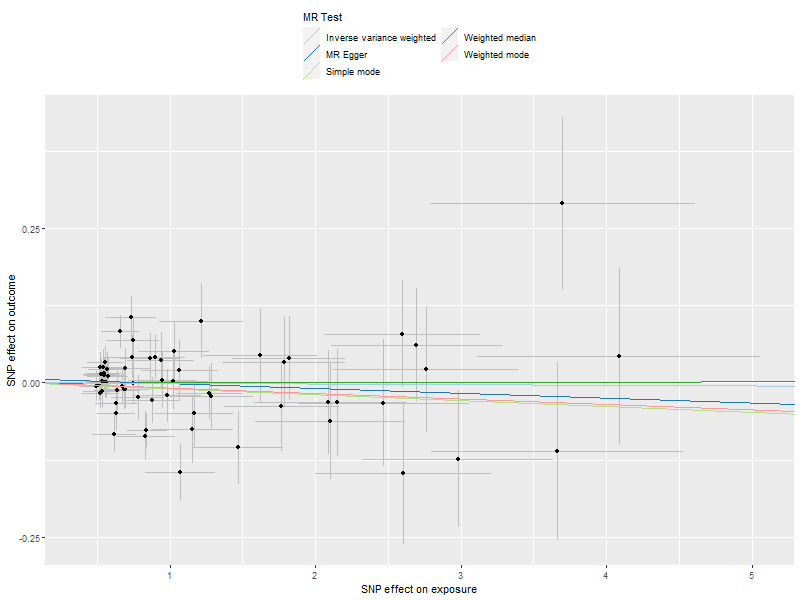


Fig S62. The scatter plot for the reverse MR analysis of HIC on AD.


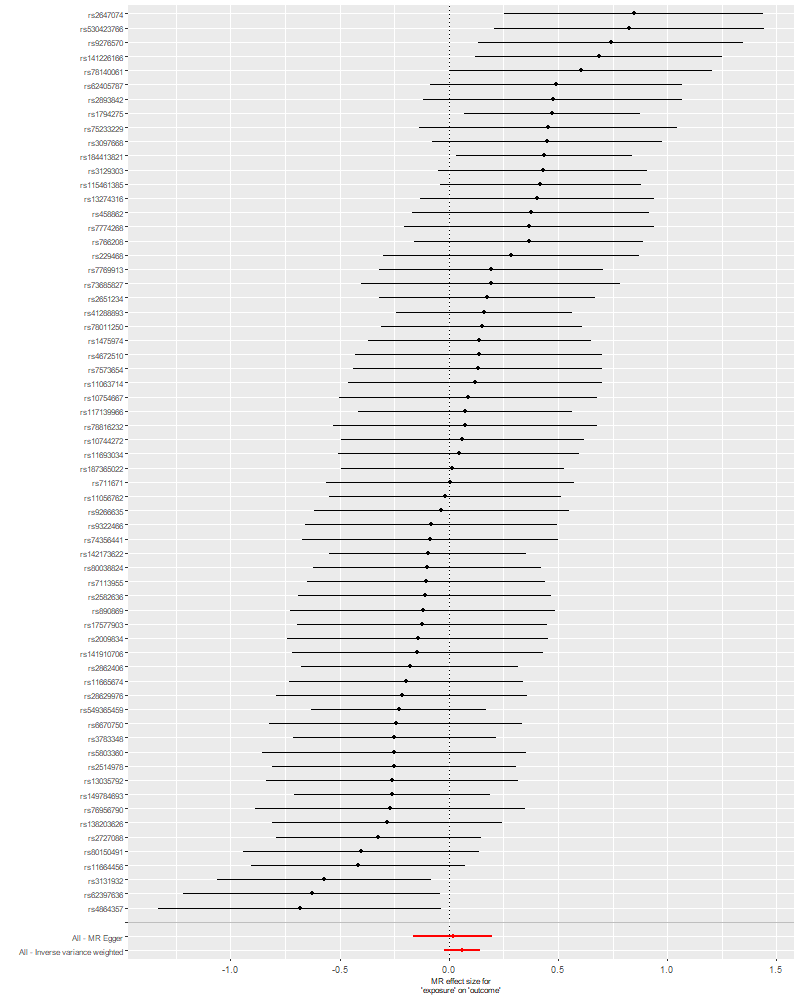


Fig S63. The forest plot of single snp for the reverse MR analysis of HIC on AIH.


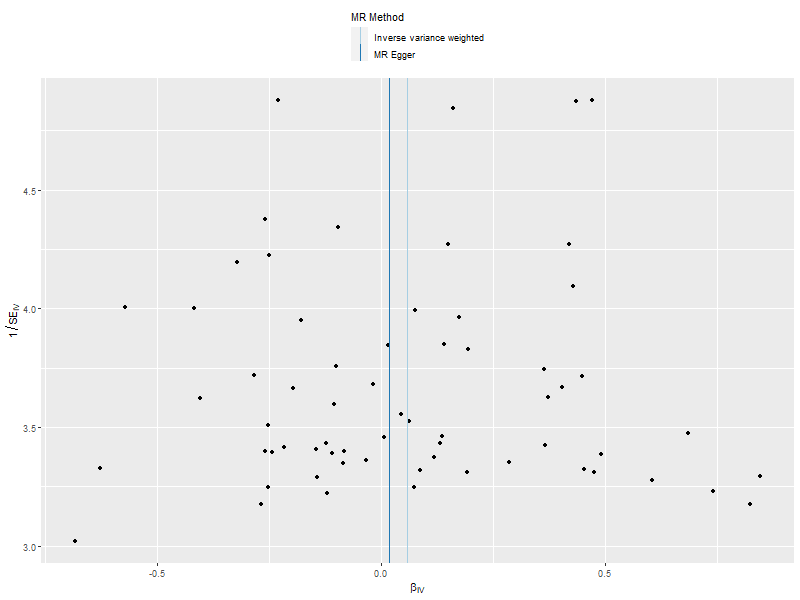


Fig S64. The funnel plot for the reverse MR analysis of HIC on AIH.


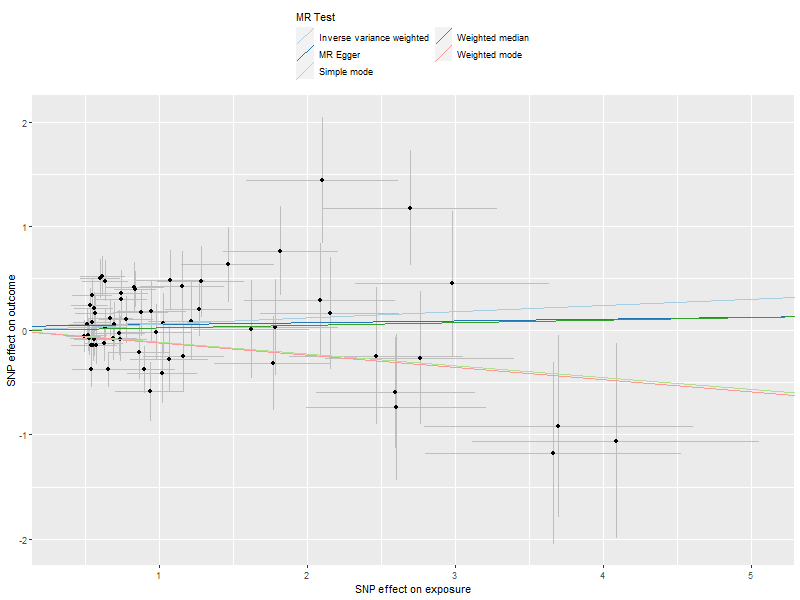


Fig S65. The scatter plot for the reverse MR analysis of HIC on AIH


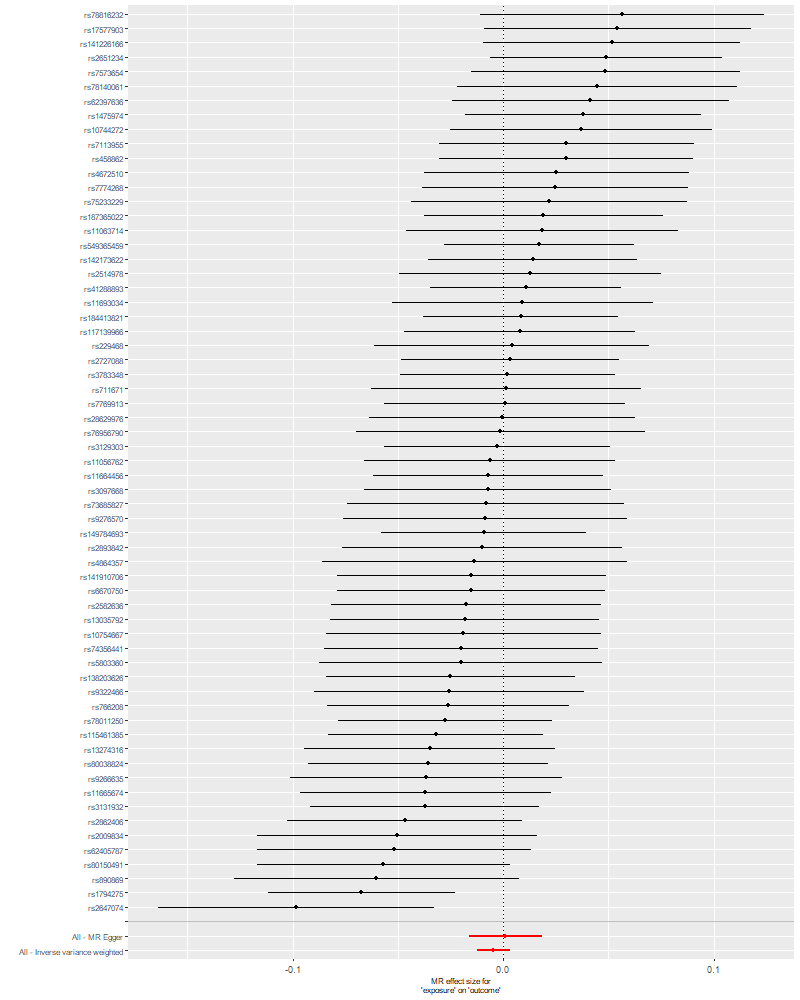


Fig S66. The forest plot of single snp for the reverse MR analysis of HIC on AR.


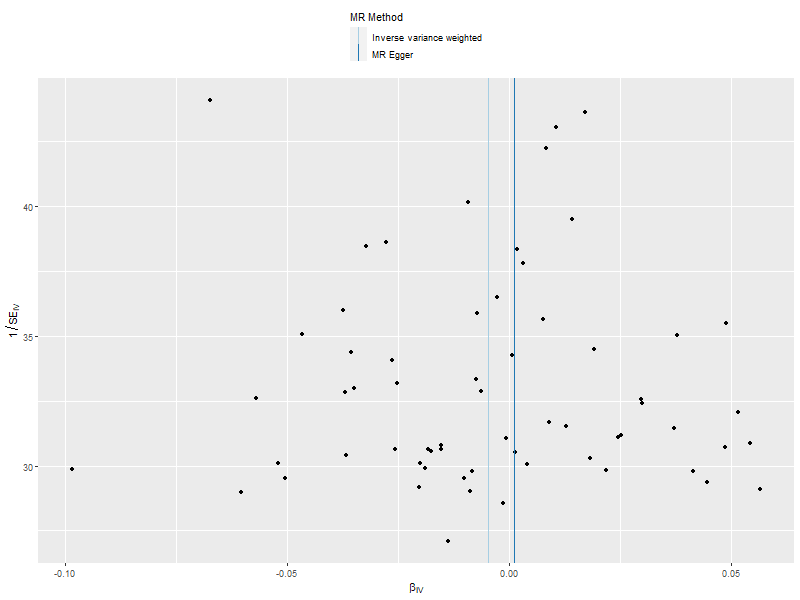


Fig S67. The funnel plot for the reverse MR analysis of HIC on AR.


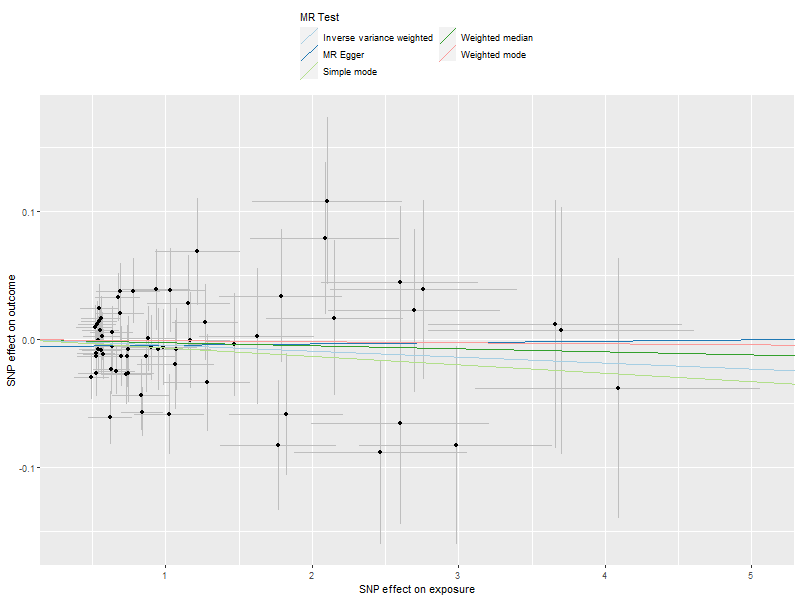


Fig S68. The scatter plot for the reverse MR analysis of HIC on AR.


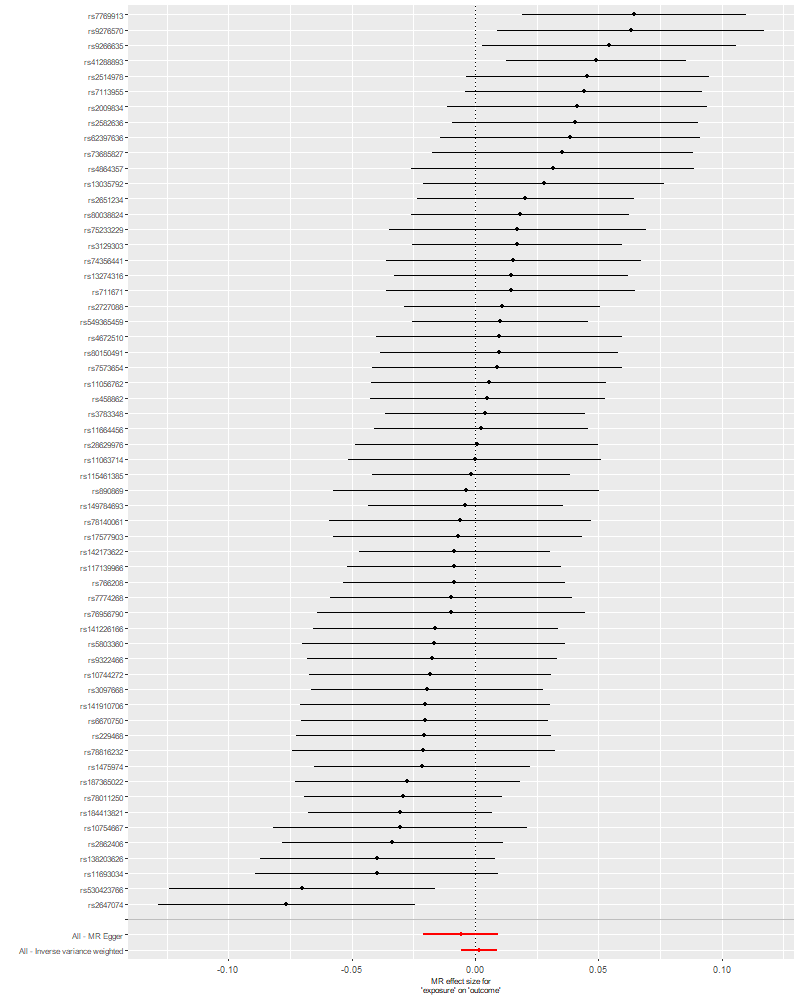


Fig S69. The forest plot of single snp for the reverse MR analysis of HIC on AS.


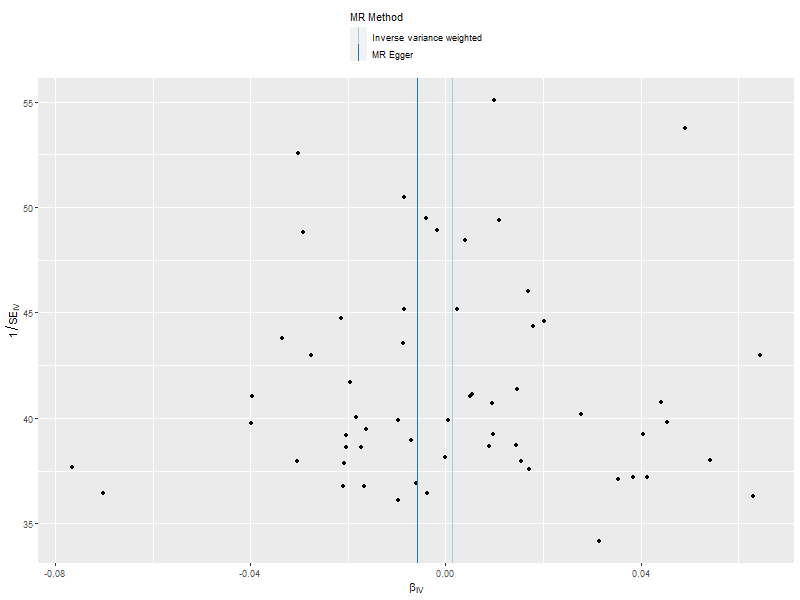


Fig S70. The funnel plot for the reverse MR analysis of HIC on AS.


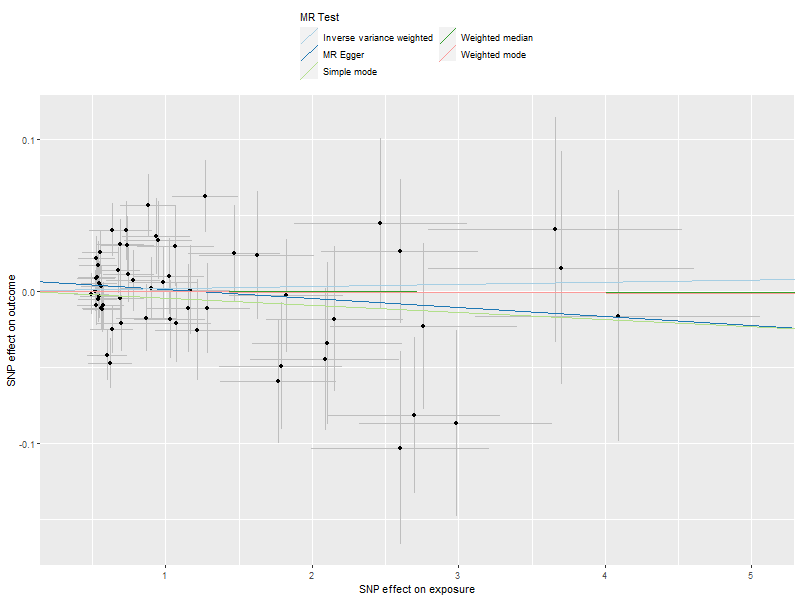


Fig S71. The scatter plot for the reverse MR analysis of HIC on AS.


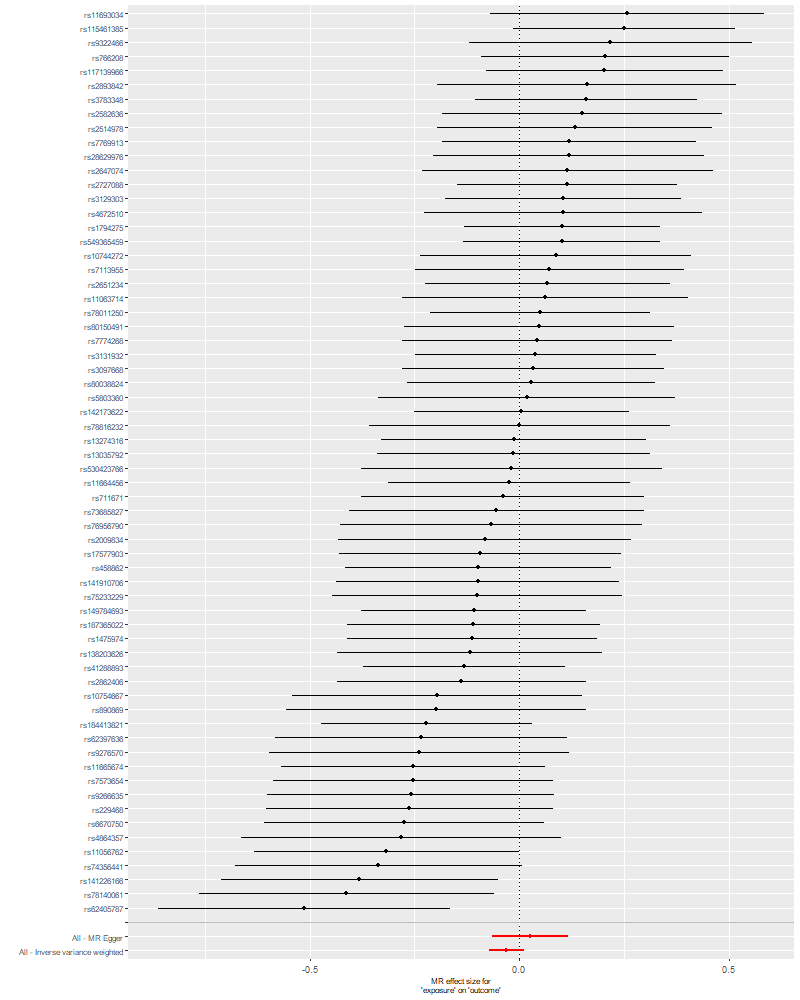


Fig S72. The forest plot of single snp for the reverse MR analysis of HIC on CD.


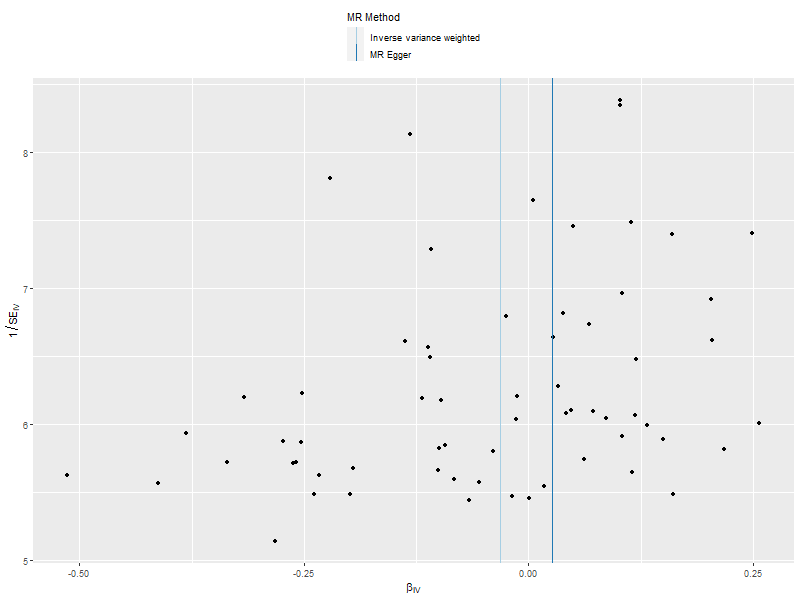


Fig S73. The funnel plot for the reverse MR analysis of HIC on CD.


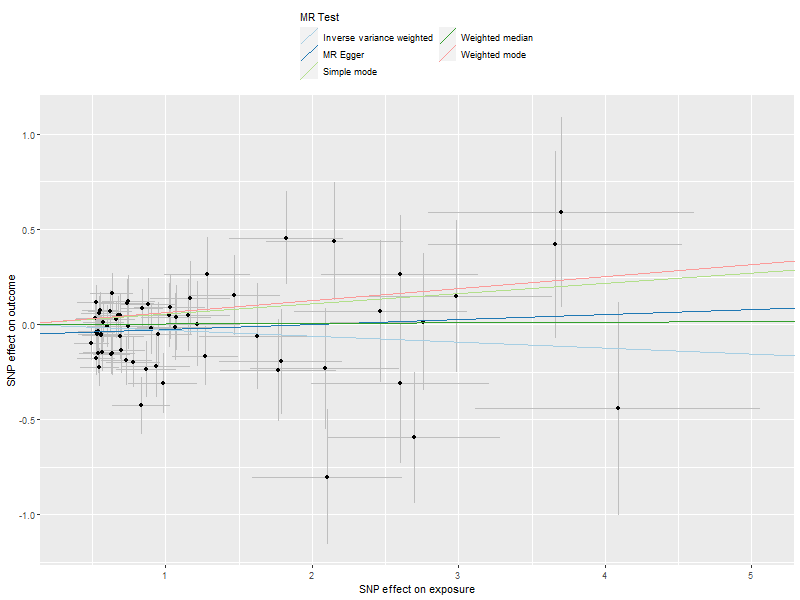


Fig S74. The scatter plot of single snp for the reverse MR analysis of HIC on CD.


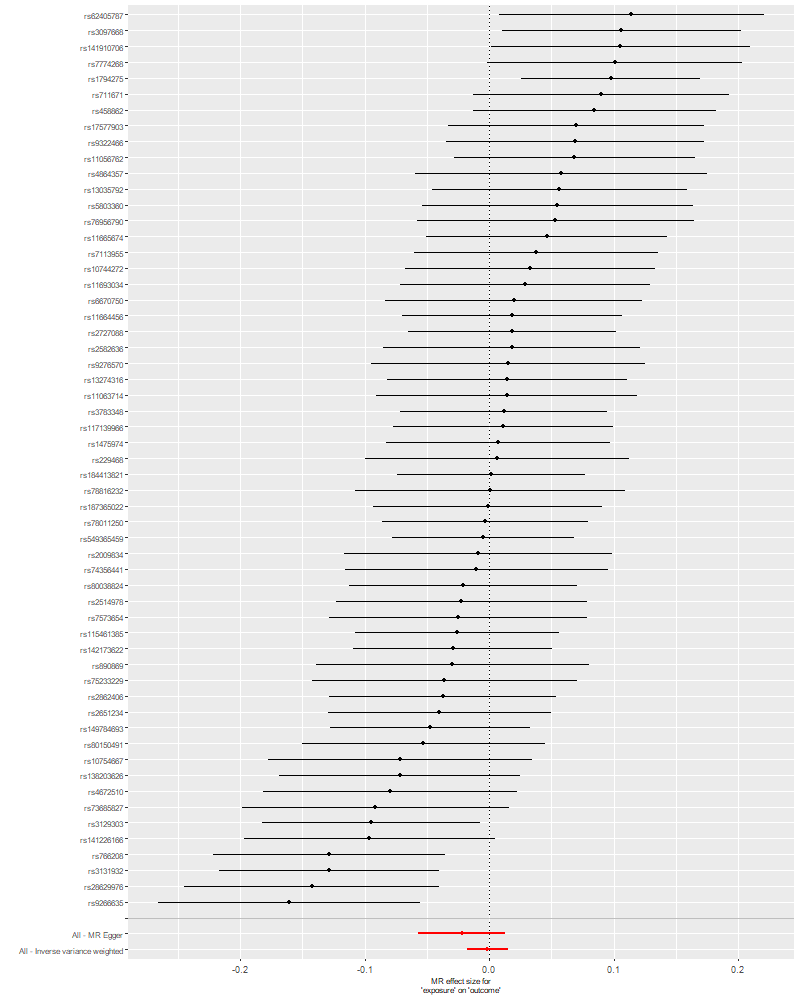


Fig S75. The forest plot for the reverse MR analysis of HIC on GD.


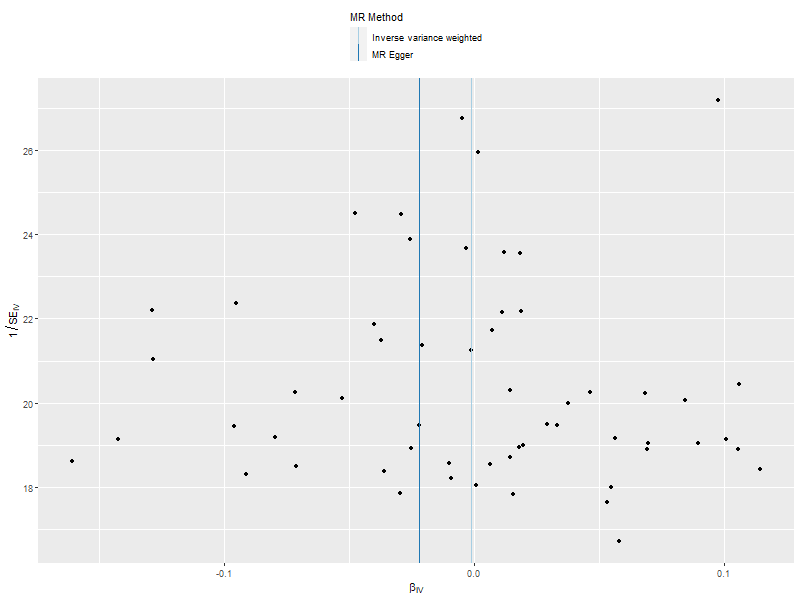


Fig S76. The funnel plot for the reverse MR analysis of HIC on GD.


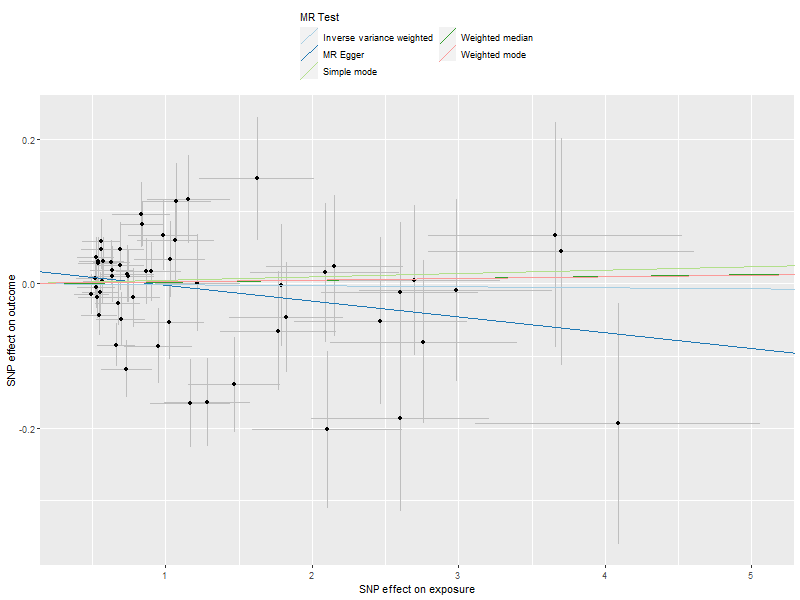


Fig S77. The scatter plot of single snp for the reverse MR analysis of HIC on GD.


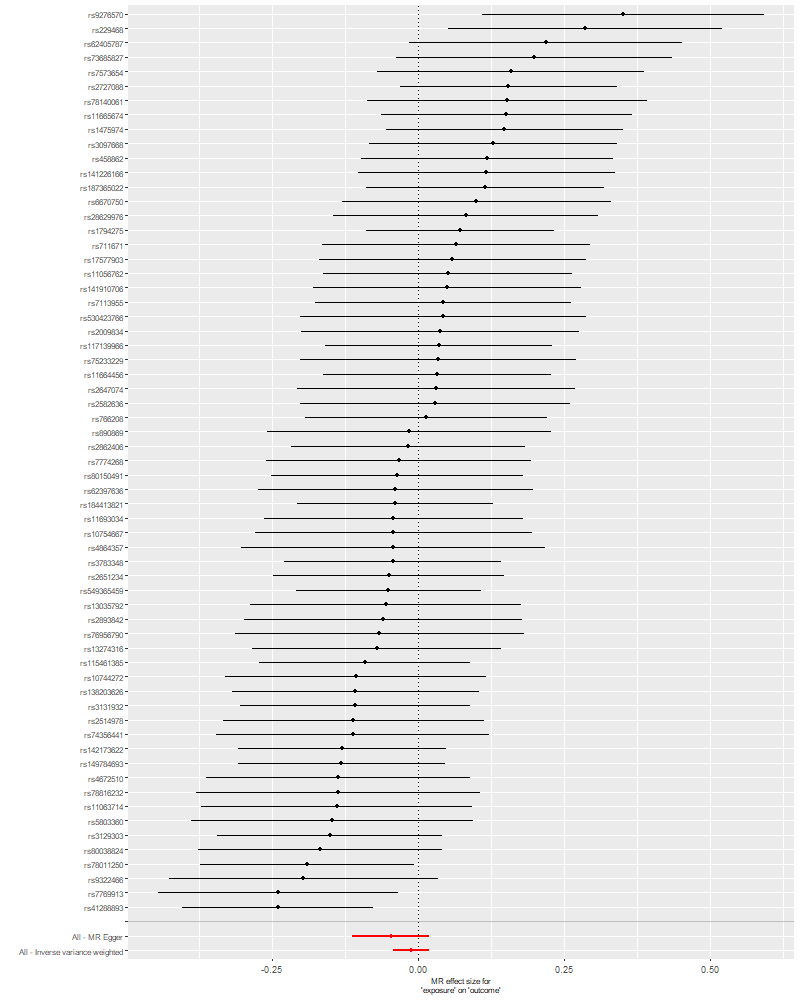


Fig S78. The forest plot of single snp for the reverse MR analysis of HIC on HT.


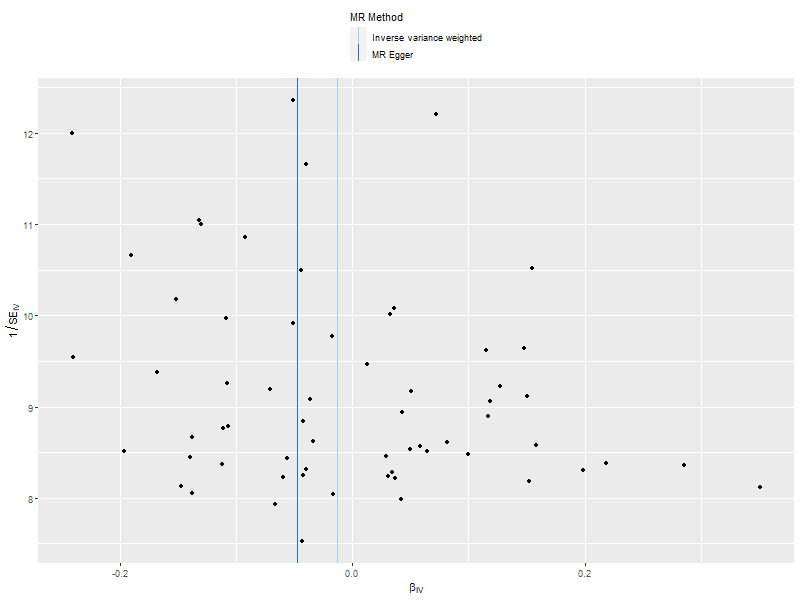


Fig S79. The funnel plot for the reverse MR analysis of HIC on HT.


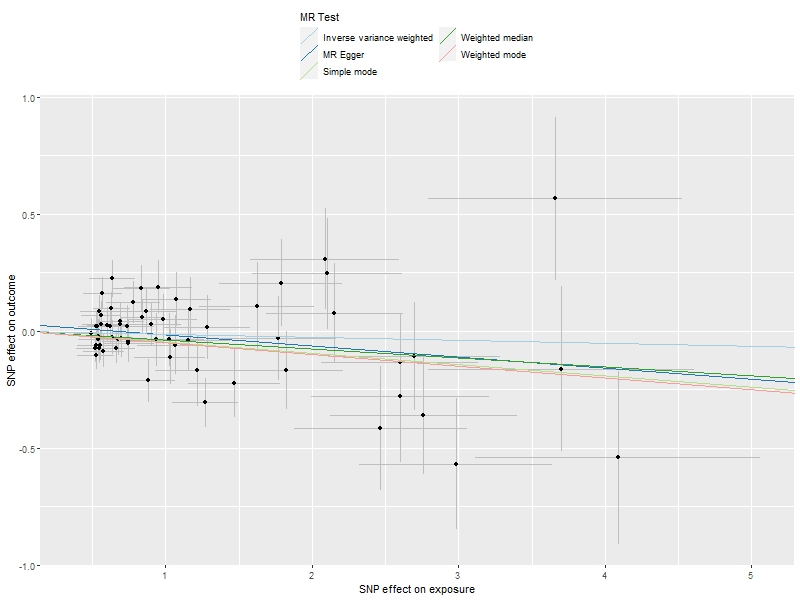


Fig S80. The scatter plot for the reverse MR analysis of HIC on HT.


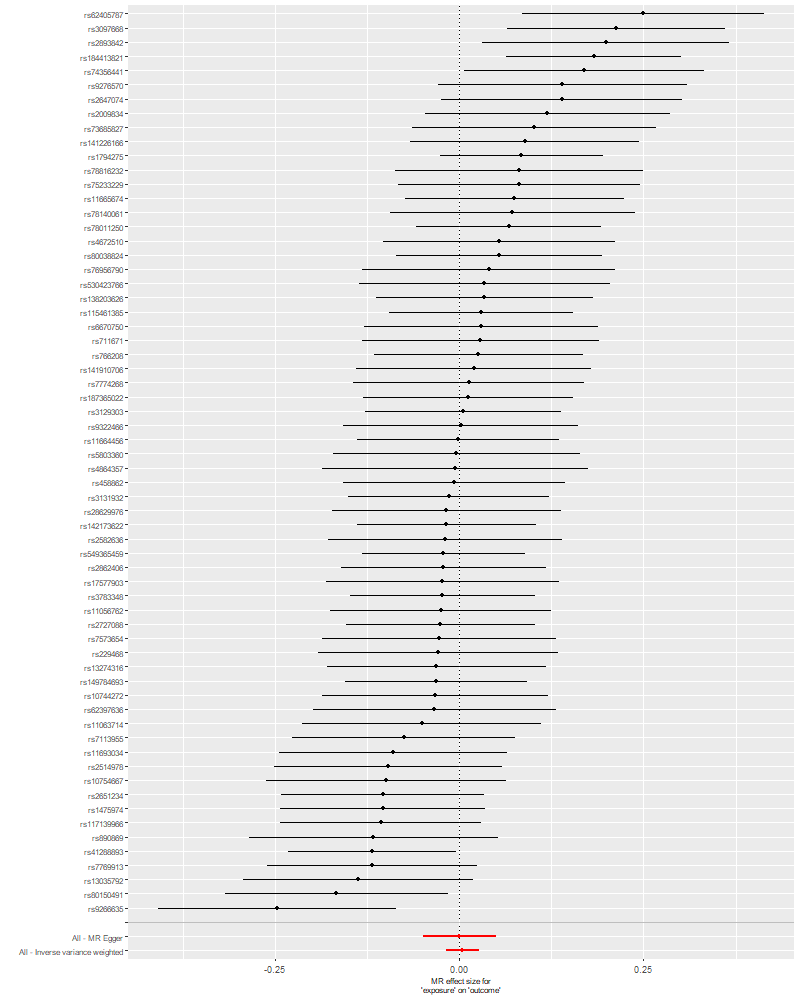


Fig S81. The forest plot of single snp for the reverse MR analysis of HIC on HY.


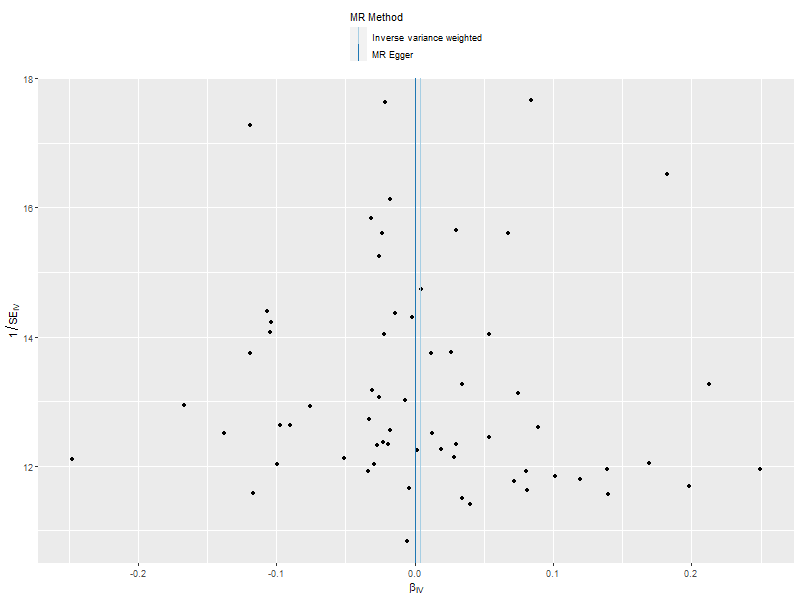


Fig S82. The funnel plot for the reverse MR analysis of HIC on HY.


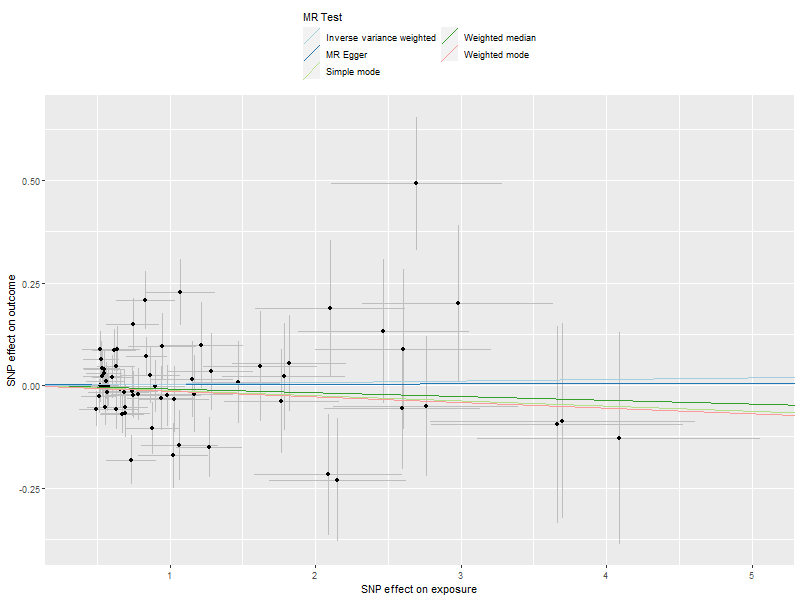


Fig S83. The scatter plot for the reverse MR analysis of HIC on HY.


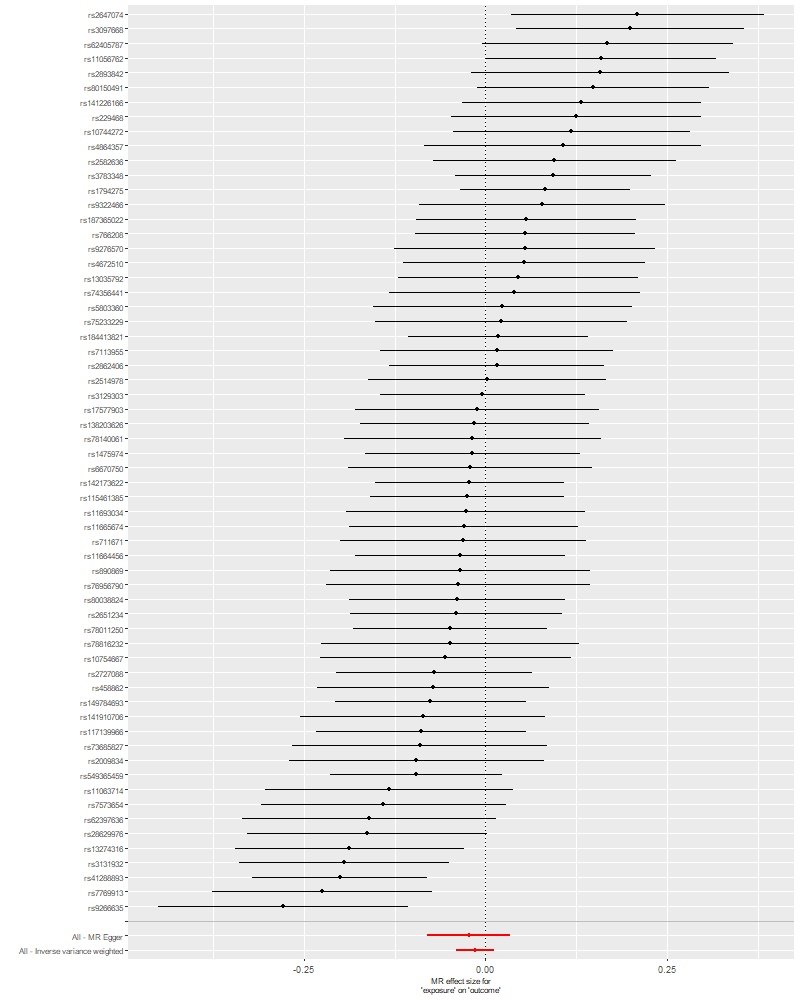


Fig S84. The forest plot of single snp for the reverse MR analysis of HIC on HYPE.


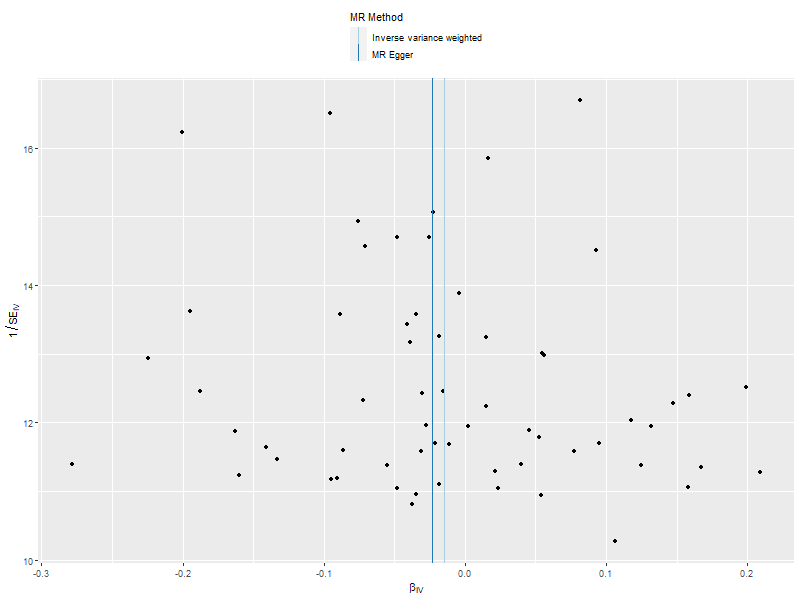


Fig S85. The funnel plot for the reverse MR analysis of HIC on HYPE.


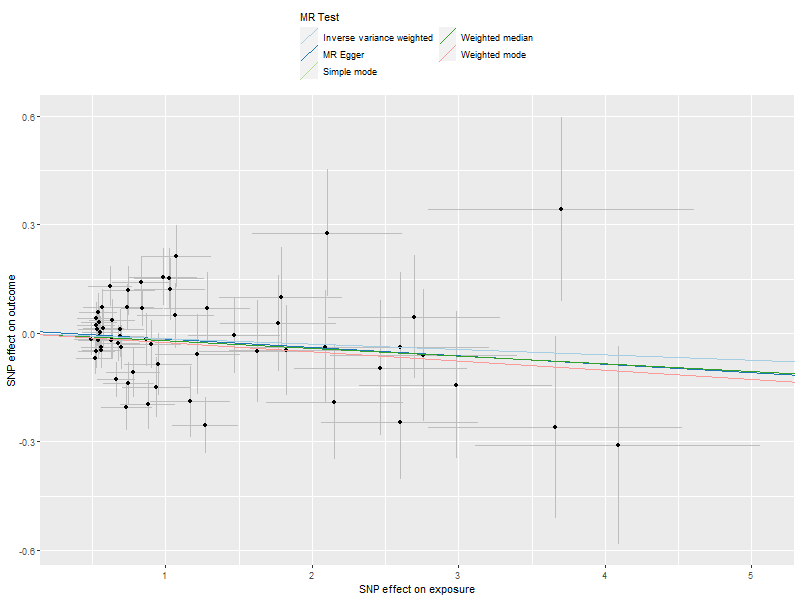


Fig S86. The scatter plot for the reverse MR analysis of HIC on HYPE.


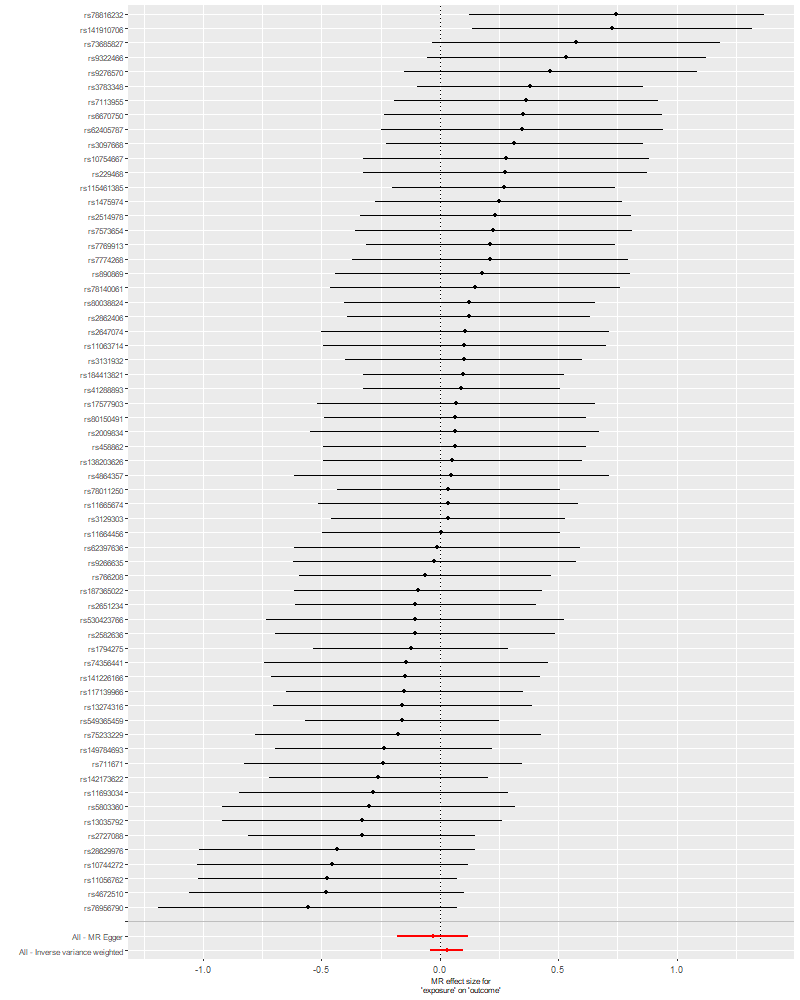


Fig S87. The forest plot of single snp for the reverse MR analysis of HIC on MG.


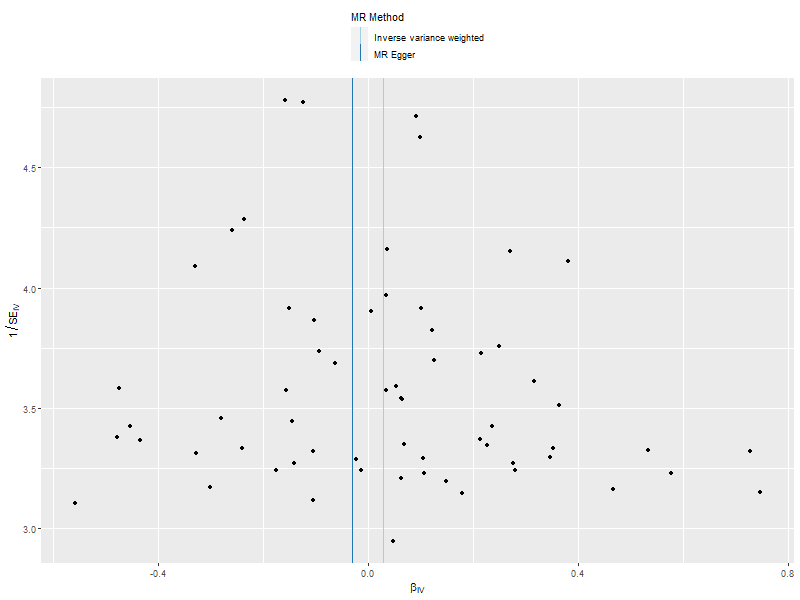


Fig S88. The funnel plot for the reverse MR analysis of HIC on MG.


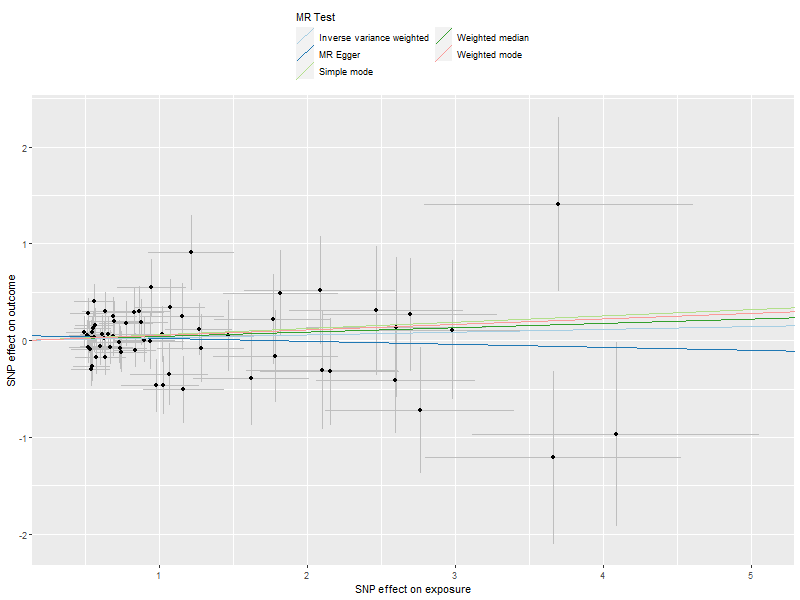


Fig S89. The scatter plot for the reverse MR analysis of HIC on MG.


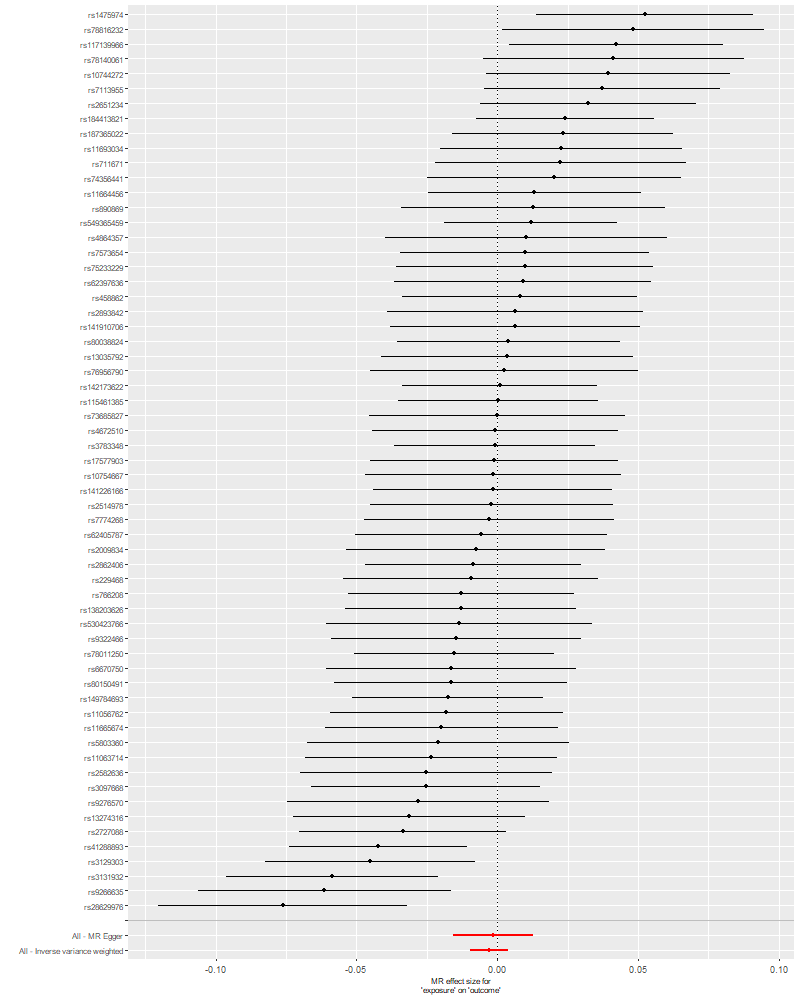


Fig S90. The forest plot of single snp for the reverse MR analysis of HIC on PO.


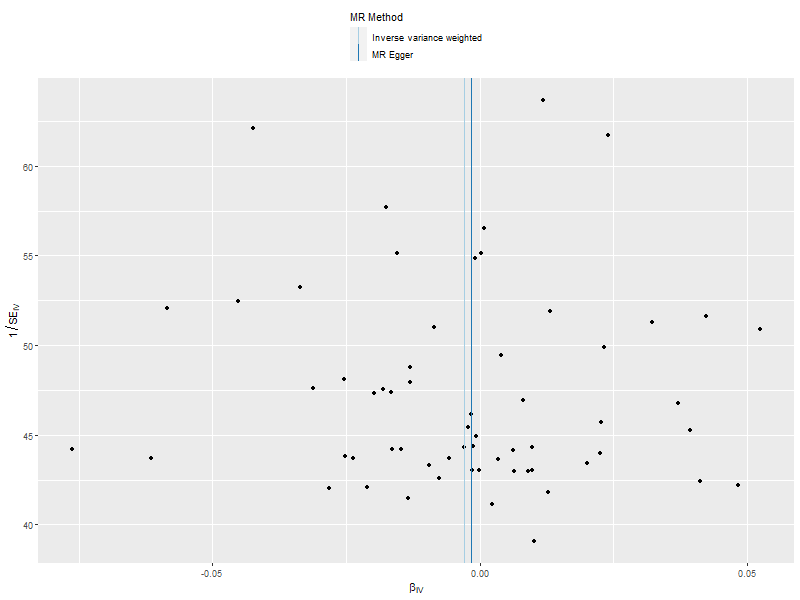


Fig S91. The funnel plot for the reverse MR analysis of HIC on PO.


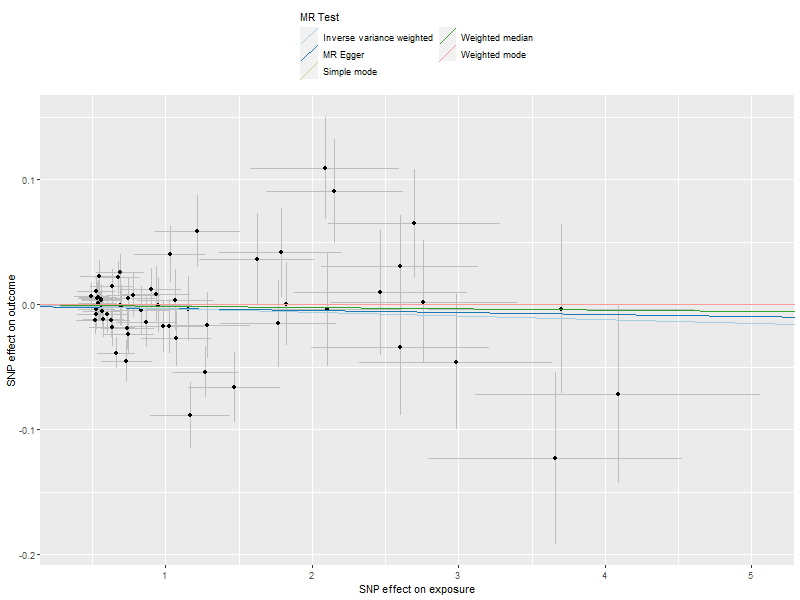


Fig S92. The scatter plot for the reverse MR analysis of HIC on PO.


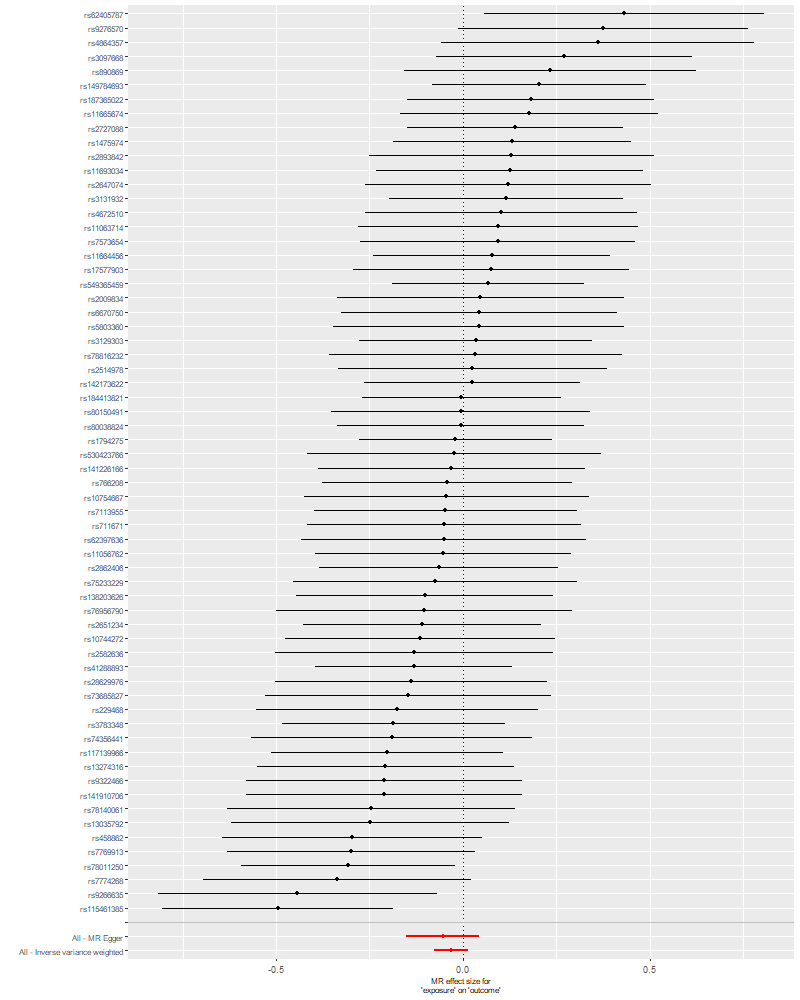


Fig S93. The forest plot of single snp for the reverse MR analysis of HIC on PV.


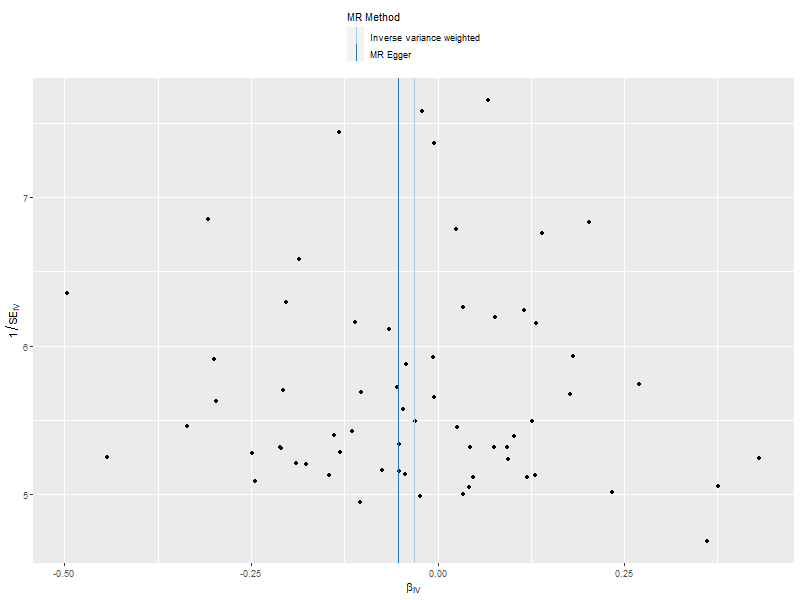


Fig S94. The funnel plot for the reverse MR analysis of HIC on PV.


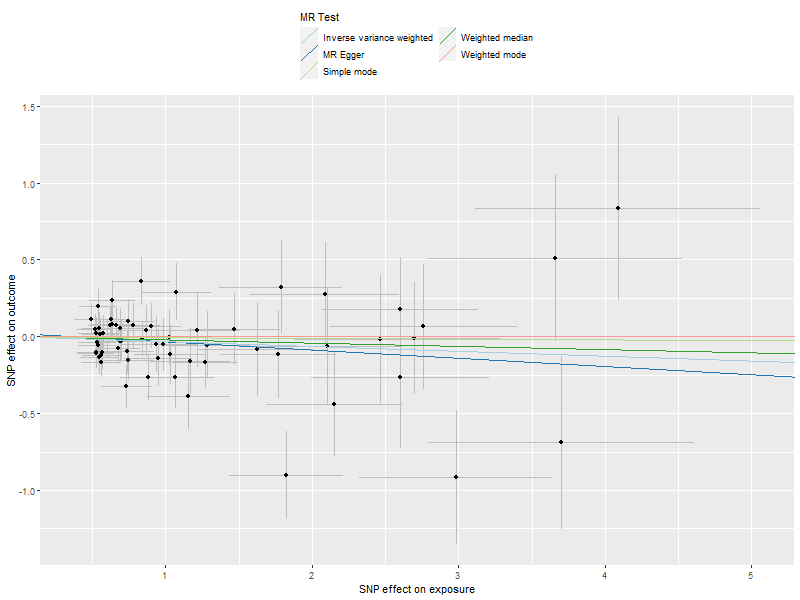


Fig S95. The scatter plot for the reverse MR analysis of HIC on PV.


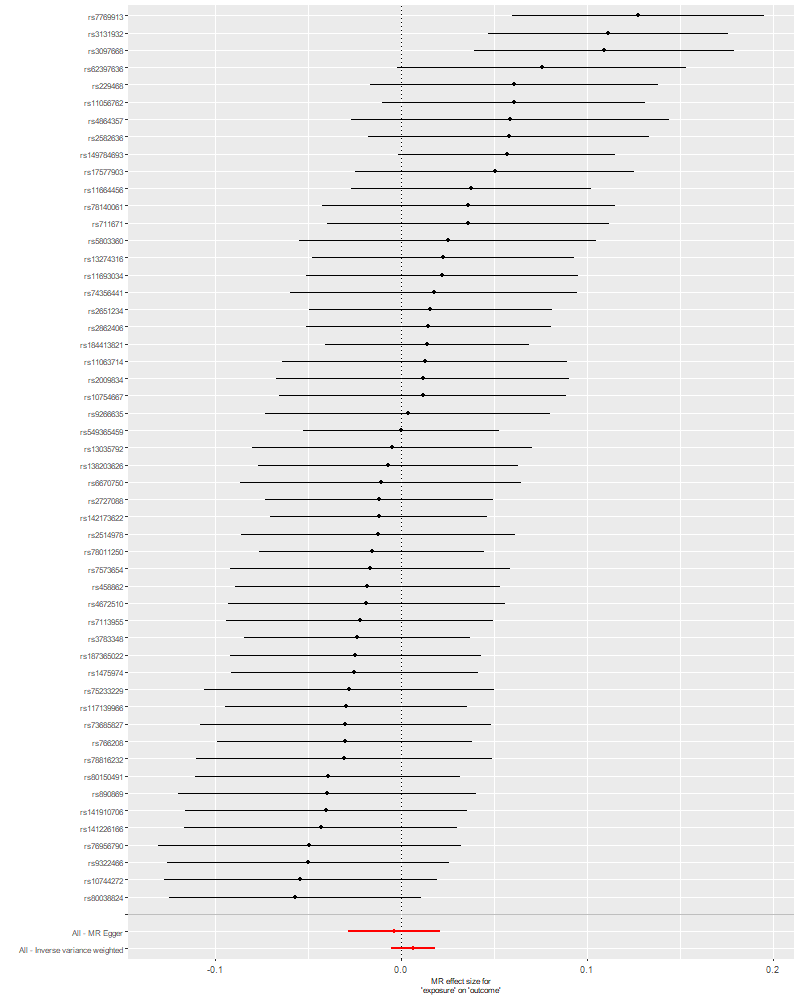


Fig S96. The forest plot of single snp for the reverse MR analysis of HIC on RA.


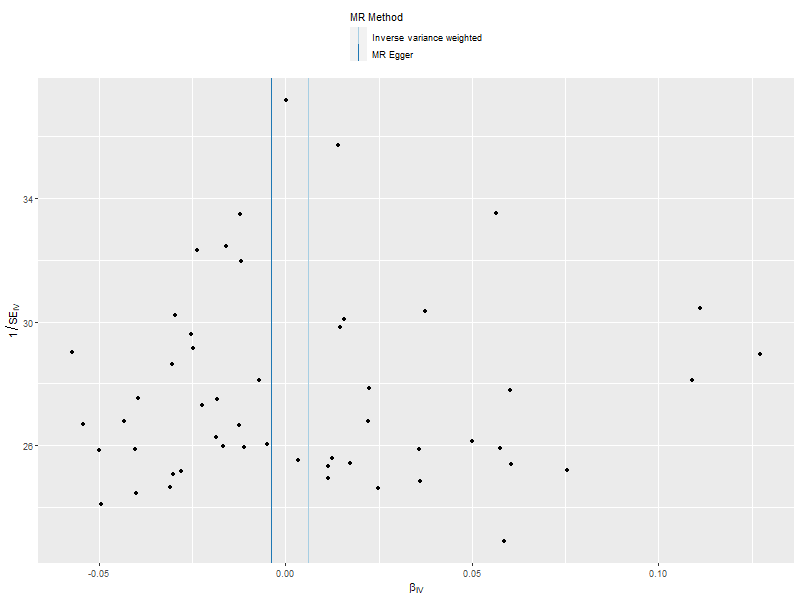


Fig S97. The funnel plot for the reverse MR analysis of HIC on RA.


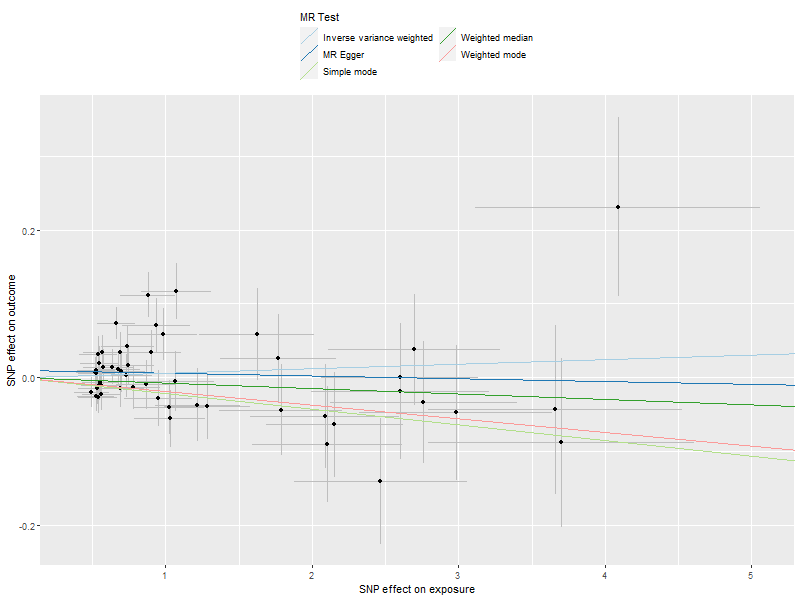


Fig S98. The scatter plot for the reverse MR analysis of HIC on RA.


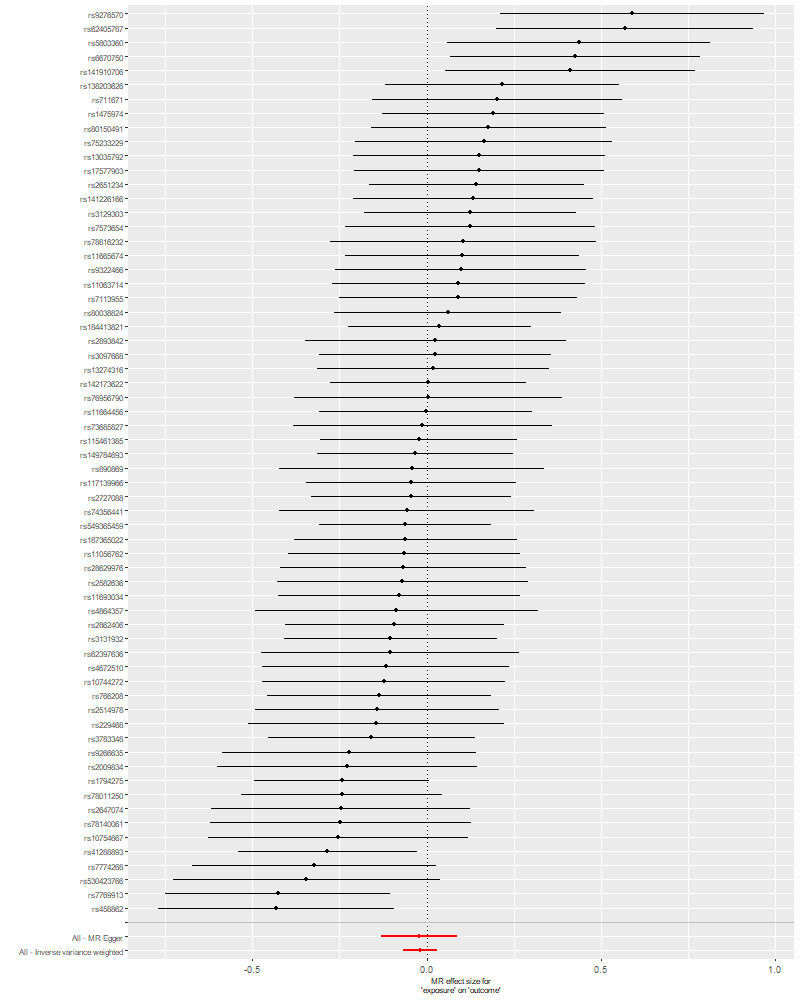


Fig S99. The forest plot of single snp for the reverse MR analysis of HIC on SA.


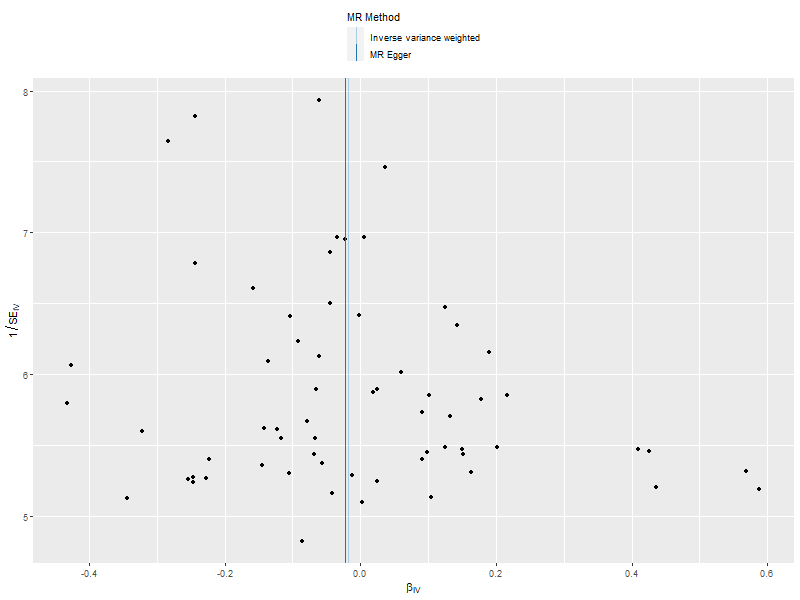


Fig S100. The funnel plot for the reverse MR analysis of HIC on SA.

Fig S101. The scatter plot for the reverse MR analysis of HIC on SA.

Fig S102. The forest plot of single snp for the reverse MR analysis of HIC on SLE.

Fig S103. The funnel plot for the reverse MR analysis of HIC on SLE.

Fig S104. The scatter plot for the reverse MR analysis of HIC on SLE.

Fig S105. The forest plot of single snp for the reverse MR analysis of HIC on SS.

Fig S106. The funnel plot for the reverse MR analysis of HIC on SS.

Fig S107. The scatter plot for the reverse MR analysis of HIC on SS.

Fig S108. The forest plot of single snp for the reverse MR analysis of HIC on T1D.

Fig S109. The funnel plot for the reverse MR analysis of HIC on T1D.

Fig S110. The scatter plot for the reverse MR analysis of HIC on T1D.

Fig S111. The forest plot of single snp for the reverse MR analysis of HIC on UC.

Fig S112. The funnel plot for the reverse MR analysis of HIC on UC.

Fig S113. The scatter plot for the reverse MR analysis of HIC on UC.

Fig S114. The forest plot of single snp for the reverse MR analysis of HIC on UV.

Fig S115. The funnel plot for the reverse MR analysis of HIC on UV.

Fig S116. The scatter plot for the reverse MR analysis of HIC on UV.

Fig S117. GTEx tissue enrichment analysis for expression of all cross-phenotype associated genes within clumping area identified by MTAG for AIH and HIC.

Fig S118. GTEx tissue enrichment analysis for expression of all cross-phenotype associated genes within clumping area identified by MTAG for RA and HIC.

Fig S119. GTEx tissue enrichment analysis for expression of all cross-phenotype associated genes within clumping area identified by MTAG for T1D and HIC.

Fig S120. GTEx tissue enrichment analysis for expression of all cross-phenotype associated genes within clumping area identified by MTAG for UC and HIC.

Fig S121. GO pathway enrichment bubble plot for cross-trait meta-analysis between AIH and HIC

Fig S122. KEGG pathway enrichment bubble plot for cross-trait meta-analysis between AIH and HIC

Fig S123. GO pathway enrichment bubble plot for cross-trait meta-analysis between RA and HIC

Fig S124. KEGG pathway enrichment bubble plot for cross-trait meta-analysis between RA and HIC

Fig S125. GO pathway enrichment bubble plot for cross-trait meta-analysis between T1D and HIC

Fig S126. KEGG pathway enrichment bubble plot for cross-trait meta-analysis between T1D and HIC

Fig S127. GO pathway enrichment bubble plot for cross-trait meta-analysis between UC and HIC

Fig S128. KEGG pathway enrichment bubble plot for cross-trait meta-analysis between UC and HIC

Fig S129. Boxplots of Shared Genes with Non-significant Differential Expression Between Control and Disease Groups

a-n. Boxplots of shared genes with non-significant differential expression between HIC and the control group (*ZSCAN9, TNXB, SFTA2, OR5V1, MUCL3, LINC01149, HLA-DQB2, NOTCH4, COL11A2, HCG9, HLA-DQB1, LINC00240, ABCF1, GABBR1*); o-t. Boxplot of the shared gene with non-significant differential expression between RA and the control group (*COL11A2, NOTCH4, GABBR1, HLA-DOA, HCG9, ZSCAN9*)
