# Supplementary material for: A genome-wide cross-trait analysis identifying shared genetic basis and causal relationships between Hunner-type interstitial cystitis and autoimmune diseases in East Asian populations
Source: Front Immunol. 2024 Nov 15;15:1417899. doi: 10.3389/fimmu.2024.1417899 (PMC11604611; doi:10.3389/fimmu.2024.1417899)
Supplement: Supplementary file 3 [file DataSheet3.docx]

**A genome-wide cross-trait analysis between Hunner-type interstitial cystitis and autoimmune disorders in East Asian ancestry.**

**Supplementary note**

**Method**

1. **Study population, design and data summary and quality control**

The workflow of our analysis was shown in Figure 1. In brief, there were three main parts in our study: causal inference analysis, cross-trait meta-analysis and post-GWAS analysis between HIC and the 19 autoimmune disorders.

- 1. **Hunner-type interstitial cystitis**

The GWAS summary statistic of HIC was obtained from the National Bioscience Database Center (NBDC) Human Database, the Dataset ID for this data is hum0197.v17.hic-gwas.v1[1], which includes a total of 153 cases and 46,087 controls. The 153 cases were recruited at Tokyo University Hospital in Japan between 2018 and 2020, while the DNA samples for the control group were sourced from the BioBank Japan project.

All control subjects did not have a history of immune-related diseases. Diagnosis of HIC was made by two urologists with expertise in managing IC/BPS, who are both board members of the East Asian IC/BPS Clinical Guidelines Committee (Y.A. and Y.H.), based on the East Asian clinical guidelines and the International Society for the Study of IC/BPS (ESSIC) criteria.1,23 All patients with HIC favorably responded to electrocautery of Hunner lesions andmanifested the histological characteristics consistent with HIC in bladder pathology. For more detailed patient information, please refer to the original article[1].

**1.2 Autoimmune disorders**

For Autoimmune disorder traits, we retrieved summary statistics from publicly available GWAS studies of atopic dermatitis (AD)[2], autoimmune hepatitis (AIH), allergic rhinitis (AR), asthma (AS), contact dermatitis (CD), Graves’ disease (GD), Hashimoto’s thyroiditis (HT), hypothyroidism (HY), hyperthyroidism (HYPE), myasthenia gravis (MG), pollinosis (PO), psoriasis vulgaris (PV), rheumatoid arthritis (RA), sarcoidosis (SA), systemic lupus erythematosus (SLE), sjogren's syndrome (SS), type 1 diabetes mellitus (T1D), ulcerative colitis (UC), uveitis (UV)[3]. Furthermore, the authors of the original GWAS obtained all necessary ethical approvals for their research. Additional details regarding each dataset can be found in Supplementary Table S1.

**1.3 Data resources**

**1.3.1 NBDC Human Database**

The GWAS data BioBank Japan (BBJ) used in this study are available from the Japanese Genotype-phenotype Archive (JGA) with accession codes JGAS000114/JGAD000123 and JGAS000114/JGAD000220, which can be accessed through application at <https://humandbs.biosciencedbc.jp/en/hum0014-latest>. In 2003, BBJ started developing one of the world’s largest disease biobanks, creating a foundation for research aimed at achieving medical care tailored to the individual traits of each patient. From a total of 260,000 patients representing 440,000 cases of 51 primarily multifactorial (common) diseases, BBJ has collected DNA, serum, medical records (clinical information), etc. with their consent. No less than 5,800 items of screened information are available for research, including the patients’ survival information, with 95% of the patients tracked over an average of 10 years. In addition to large-scale genomic analyses, omics analyses including whole genome sequencing and metabolome/proteome analyses have been performed on the DNA, serum and other biological samples collected, producing significant research findings. The genomic information acquired through the analyses continues to be used as data. The biological samples and data are widely distributed and used by researchers.

1. **Bidirectional Mendelian Randomization (MR) analysis**

To identify independent genetic instruments, we utilized the PLINK clumping function with the following parameters: clump-p1=5e-8, clump-p2=0.01, clump_kb=500Kb, and clump_r2=0.2. This allowed us to determine the top loci that were independent of each other.

We utilized several MR methods to examine the causal relationships between each ADs and HIC. Our primary MR analysis was the contamination mixture (ConMix) approach [4], which explicitly modeled multiple potential causal estimates and inferred multiple causal mechanisms associated with the same risk factor that affects the outcome to different degrees. Additionally, we also applied several sensitivity analyses to validate our results. The MR-PRESSO[5] was employed to remove outliers and ensure efficient use of valid IVs. MR-Egger regression [6] provided estimates after the correction of pleiotropy. The weighted-median (WM) estimator approach, as a median of the weighted estimates, provides a consistent effect even if half of the IVs are pleiotropic[7]. The median-based method(MBE) proceeds by constructing a kernel-weighted density of the variant-specific estimates, and taking the maximum point of this density as the point estimate. A confidence interval is obtained by bootstrapping[8].Finally, we employed the inverse-variance weighted (IVW) method[9], which is a robust approach.

We also performed several sensitivity analyses to assess the robustness of our results to potential violations of several MR assumptions. a) Heterogeneity was estimated by the Cochran Q test of IVW and MR-Egger; b) The horizontal pleiotropy was estimated using MR-Egger’s intercept; c) The influential outlier IVs due to pleiotropy was identified using MR-PRESSO’s outlier test.

The same approach was taken for the reverse MR which was used to eliminate spurious results due to reverse causation. Generally, all the analyses were conducted using R software 4.2.0. The MR-PRESSO method was performed using the “MRPRESSO” package. The IVW, MR–Egger, WM, ConMix and MBE methods were performed using the “MendelianRandomization” package. The forest plot of single snp, funnel plot and scatter plot were performed using the “TwoSampleMR” package.

**3. Cross-trait meta-analysis**

We then implemented a cross-trait meta-analysis of GWAS summary data using Multi-Trait Analysis of GWAS (MTAG)[10], a method for joint analysis of summary statistics from GWASs of different traits, to identify pleiotropic loci with strong signals associated with ADs and HIC. By analyzing multiple traits together, this approach increases the statistical power of detecting genetic associations for each trait. The MTAG estimator is a variant of the IVW meta-analysis that utilizes summary statistics from single-trait GWASs and generates trait-specific associations statistics. The resulting P-values can be considered as P-values from a single-trait GWAS. We used the BH correction for each trait’s all gene-tissue pairs on TWAS P values to account for multiple testing.

We then performed functional annotation by Functional Annotation of Variants-Online Resource (FAVOR), an open-access variant functional annotation portal for cross-trait meta-analysis, To gain biological insights into the shared loci identified between mental disorders and UI.

**4. Colocalization analysis**

We extracted summary statistics for variants within 500 kb(±250kb) of the index SNP at each of shared loci between HIC and ADs. We peformed coloclization analysis using R ‘coloc’ package[11] to calculated the probability that the two traits shared a common genetic causal variant. We caculated the posterior probability that the 2 traits were associated with different causal variants(H3) or that the 2 traits were associated and shared 1 common causal variant(H4). In our study, we considered loci with probability(PP.H4) greater than 0.4 to be colocalized[12].

**5. Fine-mapping credible set analysis**

We performed statistical fine-mapping using FINEMAP[13]. we computed LD in each locus using R package ‘LDlinkR’ [14]based on genome build GRCh37(hg19) and East Asian population of 1000 Genome project population. We defined a fine-mapping region as the 3Mb (±1.5Mb) window around each lead variant. This window size is based on recommendations for fine-mapping and colocalization analyses. We allowed up to 10 causal variants per window and extracted the posterior inclusion probabilities (PIP) of each variant using each method independently. The variants with PIP ＞0.90, along with having LD *r*^2^ ＞0.2 with the lead variant, are considered the final candidate causal variants. We applied 3DSNP, a comprehensive databases for human noncoding variants annotation, to annotate these causal variants[15].

**6.** **eQTL mapping, tissue enrichment analysis, and pathway analysis**

To map the shared SNPs between HIC and ADs traits to specific genes which they show a significant eQTL association with, we conducted the eQTL mapping analysis using the Functional Mapping and Annotation(FUMA) website [16], incorporating the SNP2GENE function with the cis-eQTLs obtained from the DICE eQTL section of the website[17], which were identified in 13 immune cell types isolated from 106 leukapheresis samples provided by 91 healthy subjects and the eQTLs obtained from the study conducted by van der Wijst et al[18], which were identified from 25,000 peripheral blood mononuclear cells (PBMCs) from 45 donors.

we conducted GTEx tissue enrichment analysis including all genes in the clumping region for each trait identified by MTAG using FUMA [16], the GENE2FUNC part with 54 tissue types from GTEx (version 8). FUMA provided us with differentially expressed gene (DEG) sets for each label of expression. The normalized expression (zero means of log2(EPLM or TPM)) and two-sided t-tests for each gene in each tissue were used for pre-calculating DEG sets. Genes were defined as DEG set in a specific tissue if Bonferroni corrected P-value<0.05 and had an absolute log-fold change≥ 0.58 (background DEGs). Genes identified by MTAG were tested against that background DEG sets by hypergeometric tests to examine if they were overrepresented in DEG sets in specific tissue types, thus, identifying the most relevant tissue types.

Additionally, we utilized the FUMA website to assess the enrichment of independent loci for each trait pairing and to explore shared genes between ADs and HIC, examining their association with Gene Ontology (GO) and Kyoto Encyclopedia of Genes and Genomes (KEGG) terms to elucidate relevant biological pathways. The method for multiple testing correction was BH with an adjusted *p-value* (FDR) cutoff (0.05).

**7. Transcriptome-wide association (TWAS)**

To explore the potential shared gene-tissue associations between ADs and HIC, we performed a TWAS using FUSION (R package), based on 49 GTEx (version 8) multi-tissue expression weights. FUSION adopts a Bayesian sparse linear mixed model (BSLMM)[19] that combines Bayesian variable selection (BVSR)[20] and linear mixed model (LMN)[21] with the normal mixture prior assumption to train weights between observed gene expressions and cis-acting genetic variants with reference dataset. This method tests the association between predicted gene expression and phenotypes of interest. Besides, we applied Benjamini-Hochberg correction for each trait’s all gene-tissue pairs on TWAS P-values, accounting for multiple tests (false discovery rate < 0.05).

**8. Extraction of TPM Expression Matrix of Genes of Interest from the GEO Database**

The microarray datasets GSE1178335, GSE5523536, GSE20636437, and GSE18167438 were extracted from the GEO database (https://www.ncbi.nlm.nih.gov/geo/). Specifically, the GSE11783 dataset for HIC is based on the GPL570 platform, the GSE55235 dataset for RA is based on the GPL96 platform, the GSE206364 dataset for AIH is based on the GPL20301 platform, and the GSE181674 dataset for T1D is based on the GPL21290 platform. We selected the TPM expression matrix of the genes of interest and performed a Wilcoxon rank-sum test to compare gene expression levels between the control and disease groups.

**URLs**

1000 Genomes Project: http://www.1000genomes.org/;

ANNOVAR: https://annovar.openbioinformatics.org/en/latest/

PLINK: https://www.cog-genomics.org/plink2;

Multi-Trait Analysis of GWAS (MTAG): https://github.com/JonJala/mtag;

Functional Annotation of Variants - Online Resource (FAVOR): http://favor.genohub.org/

Functional Mapping and Annotation (FUMA): https://fuma.ctglab.nl/;

GTEx: http://www.gtexportal.org;

Coloc: <https://chr1swallace.github.io/coloc/>

FINEMAP: http://www.christianbenner.com/

Functional Summary-based Imputation (FUSION): <https://github.com/gusevlab/fusion_twas>;

GEO: https://www.ncbi.nlm.nih.gov/geo/

Mendelian Randomization Pleiotropy RESidual Sum and Outlier (MR-PRESSO): <https://github.com/rondolab/MR-PRESSO>;

MendelianRandomization : <https://cran.r-project.org/web/packages/MendelianRandomization/index.html>;

TwoSampleMR: https://mrcieu.github.io/TwoSampleMR

R: <https://www.r-project.org/>;

**Ethics approval and consent to participate.**

This study is a secondary analysis of existing GWAS summary data from public repositories, and international research consortia. Specific and relevant ethics approval for each of the data utilised is presented in the associated publications described in the section for GWAS summary data. No additional ethics approval is required for the conduct of the present study.

**References**

[1] Akiyama Y, Sonehara K, Maeda D, Katoh H, Naito T, Yamamoto K, et al. Genome-wide association study identifies risk loci within the major histocompatibility complex region for Hunner-type interstitial cystitis. Cell Reports Medicine 2023;4:101114. https://doi.org/10.1016/j.xcrm.2023.101114.

[2] Shirai Y, Nakanishi Y, Suzuki A, Konaka H, Nishikawa R, Sonehara K, et al. Multi-trait and cross-population genome-wide association studies across autoimmune and allergic diseases identify shared and distinct genetic component. Ann Rheum Dis 2022;81:1301–12. https://doi.org/10.1136/annrheumdis-2022-222460.

[3] Sakaue S, Kanai M, Tanigawa Y, Karjalainen J, Kurki M, Koshiba S, et al. A cross-population atlas of genetic associations for 220 human phenotypes. Nat Genet 2021;53:1415–24. https://doi.org/10.1038/s41588-021-00931-x.

[4] Burgess S, Foley CN, Allara E, Staley JR, Howson JMM. A robust and efficient method for Mendelian randomization with hundreds of genetic variants. Nat Commun 2020;11:376. https://doi.org/10.1038/s41467-019-14156-4.

[5] Verbanck M, Chen CY, Neale B, Do R. Detection of widespread horizontal pleiotropy in causal relationships inferred from Mendelian randomization between complex traits and diseases. Nature Genetics 2018;50:693–8. https://doi.org/10.1038/s41588-018-0099-7.

[6] Bowden J, Davey Smith G, Burgess S. Mendelian randomization with invalid instruments: effect estimation and bias detection through Egger regression. International Journal of Epidemiology 2015;44:512–25. https://doi.org/10.1093/ije/dyv080.

[7] Bowden J, Davey Smith G, Haycock PC, Burgess S. Consistent Estimation in Mendelian Randomization with Some Invalid Instruments Using a Weighted Median Estimator. Genetic Epidemiology 2016;40:304–14. https://doi.org/10.1002/gepi.21965.

[8] Hartwig FP, Davey Smith G, Bowden J. Robust inference in summary data Mendelian randomization via the zero modal pleiotropy assumption. International Journal of Epidemiology 2017;46:1985–98. https://doi.org/10.1093/ije/dyx102.

[9] S Y, Sc L. Assessing causal associations of obesity and diabetes with kidney stones using Mendelian randomization analysis. Molecular Genetics and Metabolism 2021;134. https://doi.org/10.1016/j.ymgme.2021.08.010.

[10] Turley P, Walters RK, Maghzian O, Okbay A, Lee JJ, Fontana MA, et al. Multi-trait analysis of genome-wide association summary statistics using MTAG. Nature Genetics 2018;50:229–37. https://doi.org/10.1038/s41588-017-0009-4.

[11] Giambartolomei C, Vukcevic D, Schadt EE, Franke L, Hingorani AD, Wallace C, et al. Bayesian Test for Colocalisation between Pairs of Genetic Association Studies Using Summary Statistics. PLOS Genetics 2014;10:e1004383. https://doi.org/10.1371/journal.pgen.1004383.

[12] Chen D, Wang X, Jia J, Huang T. Sleep and Alzheimer’s Disease: Shared Genetic Risk Factors, Drug Targets, Molecular Mechanisms, and Causal Effects. In Review; 2021. https://doi.org/10.21203/rs.3.rs-853181/v1.

[13] C B, Cc S, As H, V S, S R, M P. FINEMAP: efficient variable selection using summary data from genome-wide association studies. Bioinformatics (Oxford, England) 2016;32. https://doi.org/10.1093/bioinformatics/btw018.

[14] Myers TA, Chanock SJ, Machiela MJ. LDlinkR: An R Package for Rapidly Calculating Linkage Disequilibrium Statistics in Diverse Populations. Frontiers in Genetics 2020;11. https://doi.org/10.3389/fgene.2020.00157.

[15] C Q, J P, H L, G Z, Y L. 3DSNP 2.0: update and expansion of the noncoding genomic variant annotation database. Nucleic Acids Research 2022;50. https://doi.org/10.1093/nar/gkab1008.

[16] Watanabe K, Taskesen E, van Bochoven A, Posthuma D. Functional mapping and annotation of genetic associations with FUMA. Nature Communications 2017;8:1826. https://doi.org/10.1038/s41467-017-01261-5.

[17] Schmiedel BJ, Singh D, Madrigal A, Valdovino-Gonzalez AG, White BM, Zapardiel-Gonzalo J, et al. Impact of Genetic Polymorphisms on Human Immune Cell Gene Expression. Cell 2018;175:1701-1715.e16. https://doi.org/10.1016/j.cell.2018.10.022.

[18] van der Wijst MGP, Brugge H, de Vries DH, Deelen P, Swertz MA, Franke L. Single-cell RNA sequencing identifies cell type-specific cis-eQTLs and co-expression QTLs. Nat Genet 2018;50:493–7. https://doi.org/10.1038/s41588-018-0089-9.

[19] Zhou X, Carbonetto P, Stephens M. Polygenic modeling with bayesian sparse linear mixed models. PLoS Genetics 2013;9:e1003264. https://doi.org/10.1371/journal.pgen.1003264.

[20] Guan Y, Stephens M. Bayesian variable selection regression for genome-wide association studies and other large-scale problems. The Annals of Applied Statistics 2011;5:1780–815, 36.

[21] Yu J, Pressoir G, Briggs WH, Vroh Bi I, Yamasaki M, Doebley JF, et al. A unified mixed-model method for association mapping that accounts for multiple levels of relatedness. Nature Genetics 2006;38:203–8. https://doi.org/10.1038/ng1702.
