# Supplementary material for: A genome-wide cross-trait analysis identifying shared genetic basis and causal relationships between Hunner-type interstitial cystitis and autoimmune diseases in East Asian populations
Source: Front Immunol. 2024 Nov 15;15:1417899. doi: 10.3389/fimmu.2024.1417899 (PMC11604611; doi:10.3389/fimmu.2024.1417899)
Supplement: Supplementary file 4 [file SupplementaryFile1.docx]

**STROBE-MR checklist of recommended items to address in reports of Mendelian randomization studies**^1^ ^2^

| **Item No.** | **Section** | **Checklist item** | **Page No.** | **Relevant text from manuscript** |
| --- | --- | --- | --- | --- |
| 1 | **TITLE and ABSTRACT** | Indicate Mendelian randomization (MR) as the study’s design in the title and/or the abstract if that is a main purpose of the study |  | A genome-wide cross-trait analysis identifying shared genetic basis and causal relationships between Hunner-type interstitial cystitis and autoimmune diseases in East Asian populations  Abstract  Purpose: Epidemiological studies have demonstrated the clinical link between Hunner interstitial cystitis (HIC) and autoimmune diseases (ADs), suggesting potential shared genetic bases for their comorbidity. We aimed to investigate the shared genetic architecture and causal relationships between HIC and ADs.  Methods: We conducted a genome-wide cross-trait study with ~170000 individuals of East Asian ancestry to investigate the shared architecture between HIC and ADs. Bidirectional Mendelian randomization (MR) was used to assess potential causal relationships and a multi-trait analysis of GWAS (MTAG) was conducted to identify their associated pleiotropic loci. Fine-mapping analysis narrowed candidate gene susceptibility loci and colocalization analysis was performed to identify shared variants at specific locus. Lastly, transcriptome-wide association (TWAS) and functional analysis were utilized to explore potential shared gene-tissue associations.  Results: Through bidirectional MR analysis, we observed a positive causal effect of AIH(ORIVW=1.09, PIVW=1.00×10-3) and RA (ORIVW=1.47, PIVW<1.00×10-4) on HIC and a negative causal effect of UC on HIC (ORIVW=0.89, PIVW< 1.00×10-4). Furthermore, we unveiled a robust positive causal effect of HIC on T1D(ORConMix=1.05, PConMix=1.77×10-3). Cross-trait meta-analysis identified a total of 64 independent SNPs associated with HIC and ADs. Functional analysis revealed that the identified variants regulated gene expression in major tissues belonging to the autoimmune system.  Conclusions: Our findings might offer insights into the shared underlying etiology of HIC and ADs.  Keywords: Cross-trait analysis; Genetic epidemiology; Mendelian randomization; Hunner-type interstitial cystitis; Autoimmune disorder |
|  | **INTRODUCTION** |  |  |  |
| 2 | **Background** | Explain the scientific background and rationale for the reported study. What is the exposure? Is a potential causal relationship between exposure and outcome plausible? Justify why MR is a helpful method to address the study question |  | **Manuscript**  The pathogenic mechanisms underlying HIC remain unclear, although previous studies have suggested that it involves complex interactions between multiple mechanisms, including neural, endocrine, and immune factors1,3. Immunoglobulin and complement deposition, aggregation of restricted light-chain plasma cells, and upregulation of pro-inflammatory genes/molecules involved in innate and adaptive immune responses have been detected in the bladder tissues of HIC patients4–8…… |
| 3 | **Objectives** | State specific objectives clearly, including pre-specified causal hypotheses (if any). State that MR is a method that, under specific assumptions, intends to estimate causal effects |  | **Manuscript**  In this study, we conducted a large-scale genome-wide cross-trait association study with ~170000 individuals of East Asian ancestry to investigate the shared architecture between HIC and Ads…… |
|  | **METHODS** |  |  |  |
| 4 | **Study design and data sources** | Present key elements of the study design early in the article. Consider including a table listing sources of data for all phases of the study. For each data source contributing to the analysis, describe the following: |  | **Manuscript**  The workflow of our analysis was shown in Fig 1. In brief, there were three main parts in our study: causal inference analysis, cross-trait meta-analysis and post-GWAS analysis between HIC and the 19 autoimmune disorders…… |
|  | a) | Setting: Describe the study design and the underlying population, if possible. Describe the setting, locations, and relevant dates, including periods of recruitment, exposure, follow-up, and data collection, when available. |  | **Manuscript**  The GWAS summary statistic of HIC includes a total of 153 cases and 46,087 controls13. The 153 cases were recruited at Tokyo University Hospital in Japan between 2018 and 2020. DNA samples of controls were obtained from the Biobank Japan Project (BBJ). The autoimmune diseases included……  **supplementary table** |
|  | b) | Participants: Give the eligibility criteria, and the sources and methods of selection of participants. Report the sample size, and whether any power or sample size calculations were carried out prior to the main analysis |  | **Manuscript**  The GWAS summary statistic of HIC includes a total of 153 cases and 46,087 controls13. The 153 cases were recruited at Tokyo University Hospital in Japan between 2018 and 2020. DNA samples of controls were obtained from the Biobank Japan Project (BBJ). The autoimmune diseases included AD(Ncase/Ncontrol=2472/142192, AIH(Ncase/Ncontrol=85/166529), AR(Ncase/Ncontrol=7897/153666), AS(Ncase/Ncontrol=13015/162933), CD(Ncase/Ncontrol=247/161777), GD(Ncase/Ncontrol=2809/172656), HT(Ncase/Ncontrol=537/172656), HY(Ncase/Ncontrol=1114/172656), HYPE(Ncase/Ncontrol=994/172656), MG(Ncase/Ncontrol=81/178630), PO(Ncase/Ncontrol=18593/153666), RA(Ncase/Ncontrol=5348/173268), SA(Ncase/Ncontrol=220/177667), SLE(Ncase/Ncontrol=317/175937), SS(Ncase/Ncontrol=303/175599), T1D(Ncase/Ncontrol=1219/132032), UC(Ncase/Ncontrol=314/178375), UV(Ncase/Ncontrol=125/174600)……  **Supplementary table1**  **Supplementary note** |
|  | c) | Describe measurement, quality control and selection of genetic variants |  | **Manuscript**  To identify independent genetic instruments, we utilized the PLINK clumping function with the following parameters: clump-p1=5e-8, clump-p2=0.01, clump_kb=500Kb, and clump_r2=0.2. This allowed us to determine the top loci that were independent of each other. To ensure statistical power due to the limited number of instrumental variables (n<10), we employed more lenient criteria for instrument variable selection: clump-p1=5e-6, clump-p2=0.01, clump_kb=500Kb, and clump_r2=0.2. Furthermore, we applied Steiger filtering to the instrumental variables and excluded instruments with F-statistics<10. |
|  | d) | For each exposure, outcome, and other relevant variables, describe methods of assessment and diagnostic criteria for diseases |  | **Manuscript**  All control subjects did not have a history of immune-related diseases. Diagnosis of HIC was made by two urologists with expertise in managing IC/BPS, who are both board members of the East Asian IC/BPS Clinical Guidelines Committee (Y.A. and Y.H.), based on the East Asian clinical guidelines and the International Society for the Study of IC/BPS (ESSIC) criteria.1,23 All patients with HIC favorably responded to electrocautery of Hunner lesions andmanifested the histological characteristics consistent with HIC in bladder pathology. For more detailed patient information, please refer to the original article[1].  **Supplementary note** |
|  | e) | Provide details of ethics committee approval and participant informed consent, if relevant |  | **Manuscript**  Furthermore, the authors of the original GWAS obtained all necessary ethical approvals for their research  **Supplementary note** |
| 5 | **Assumptions** | Explicitly state the three core IV assumptions for the main analysis (relevance, independence and exclusion restriction) as well assumptions for any additional or sensitivity analysis |  | **Manuscript**  We also performed several sensitivity analyses to assess the robustness of our results to potential violations of several MR assumptions. a) Heterogeneity was estimated by the Cochran Q test of IVW and MR-Egger; b) The horizontal pleiotropy was estimated using MR-Egger’s intercept; c) The influential outlier IVs due to pleiotropy was identified using MR-PRESSO’s outlier test. |
| 6 | **Statistical methods: main analysis** | Describe statistical methods and statistics used |  |  |
|  | a) | Describe how quantitative variables were handled in the analyses (i.e., scale, units, model) |  | **Supplementary table 1** |
|  | b) | Describe how genetic variants were handled in the analyses and, if applicable, how their weights were selected |  | **Manuscript**  the GWAS summary statistics of were obtained from NBDC Human Database, with the Dataset ID hum0197.v3.gwas.v114. We used ANNOVAR15 to annotate variants of GWAS summary statistics based on ‘hg19 avsnp150’. |
|  | c) | Describe the MR estimator (e.g. two-stage least squares, Wald ratio) and related statistics. Detail the included covariates and, in case of two-sample MR, whether the same covariate set was used for adjustment in the two samples |  | **Manuscript**  We utilized several MR methods to examine the causal relationships between each ADs and HIC. Our primary MR analysis was the contamination mixture (ConMix) approach 16, which explicitly modeled multiple potential causal estimates and inferred multiple causal mechanisms associated with the same risk factor that affects the outcome to different degrees. Additionally, we also applied several sensitivity analyses to validate our results. The MR-PRESSO17 was employed to remove outliers and ensure efficient use of valid IVs. MR-Egger regression 18 provided estimates after the correction of pleiotropy. The weighted-median (WM) estimator approach, as a median of the weighted estimates, provides a consistent effect even if half of the IVs are pleiotropic19. The median-based method(MBE) proceeds by constructing a kernel-weighted density of the variant-specific estimates, and taking the maximum point of this density as the point estimate. A confidence interval is obtained by bootstrapping20. Finally, we employed the inverse-variance weighted (IVW) method21, which is a robust approach. |
|  | d) | Explain how missing data were addressed |  | **Manuscript**  After removing the outlier instrumental variables (IVs) identified by MR-PRESSO, we conducted the MR analysis again. |
|  | e) | If applicable, indicate how multiple testing was addressed |  | **Manuscript**  We corrected multiple testing for MR P-values by the Bonferroni method, and a P-value of 0.00263 (0.05/19) was considered as the significant level. |
| 7 | **Assessment of assumptions** | Describe any methods or prior knowledge used to assess the assumptions or justify their validity |  | **Manuscript**  Furthermore, IC may be a systemic disease and is often comorbid with various autoimmune diseases (ADs). Numerous studies have demonstrated an increased prevalence of multiple ADs in IC patients, including rheumatoid arthritis (RA), systemic lupus erythematosus (SLE), Sjögren's syndrome (SS), inflammatory bowel disease, and autoimmune thyroid diseases9–12  Furthermore, we applied Steiger filtering to the instrumental variables and excluded instruments with F-statistics<10. |
| 8 | **Sensitivity analyses and additional analyses** | Describe any sensitivity analyses or additional analyses performed (e.g. comparison of effect estimates from different approaches, independent replication, bias analytic techniques, validation of instruments, simulations) |  | **Manuscript**  Additionally, we also applied several sensitivity analyses to validate our results. The MR-PRESSO17 was employed to remove outliers and ensure efficient use of valid IVs. MR-Egger regression 18 provided estimates after the correction of pleiotropy. The weighted-median (WM) estimator approach, as a median of the weighted estimates, provides a consistent effect even if half of the IVs are pleiotropic19. The median-based method(MBE) proceeds by constructing a kernel-weighted density of the variant-specific estimates, and taking the maximum point of this density as the point estimate. A confidence interval is obtained by bootstrapping20. Finally, we employed the inverse-variance weighted (IVW) method  The same approach was taken for the reverse MR which was used to eliminate spurious results due to reverse causation.  We extracted summary statistics for variants within 500 kb(±250kb) of the index SNP at each of the shared loci between HIC and ADs and performed colocalization analysis between HIC and each ADs trait using R ‘coloc’ package25 |
| 9 | **Software and pre-registration** |  |  |  |
|  | a) | Name statistical software and package(s), including version and settings used |  | **Manuscript**  The same approach was taken for the reverse MR which was used to eliminate spurious results due to reverse causation. Generally, all the analyses were conducted using R software 4.2.0. The MR-PRESSO method was performed using the “MRPRESSO” package. The IVW, MR–Egger, WM, ConMix and MBE methods were performed using the “MendelianRandomization” package. The forest plot of single SNP, funnel plot and scatter plot were performed using the “TwoSampleMR” package.  We then implemented a cross-trait meta-analysis of GWAS summary data using Multi-Trait Analysis of GWAS (MTAG)27, a method for joint analysis of summary statistics from GWASs of different traits, to identify pleiotropic loci with strong signals associated with ADs and HIC.  **Supplementary note** |
|  | b) | State whether the study protocol and details were pre-registered (as well as when and where) |  | **Supplementary note and Fig1** |
|  | **RESULTS** |  |  |  |
| 10 | **Descriptive data** |  |  |  |
|  | a) | Report the numbers of individuals at each stage of included studies and reasons for exclusion. Consider use of a flow diagram |  | **Fig1-2** |
|  | b) | Report summary statistics for phenotypic exposure(s), outcome(s), and other relevant variables (e.g. means, SDs, proportions) |  | **Supplementary table1** |
|  | c) | If the data sources include meta-analyses of previous studies, provide the assessments of heterogeneity across these studies |  | This study does not include this item. |
|  | d) | For two-sample MR:  i.  Provide justification of the similarity of the genetic variant-exposure associations between the exposure and outcome samples  ii.  Provide information on the number of individuals who overlap between the exposure and outcome studies |  | **Manuscript**  Furthermore, IC may be a systemic disease and is often comorbid with various autoimmune diseases (ADs). Numerous studies have demonstrated an increased prevalence of multiple ADs in IC patients, including rheumatoid arthritis (RA), systemic lupus erythematosus (SLE), Sjögren's syndrome (SS), inflammatory bowel disease, and autoimmune thyroid diseases9–12  **Supplementary table1** |
| 11 | **Main results** |  |  |  |
|  | a) | Report the associations between genetic variant and exposure, and between genetic variant and outcome, preferably on an interpretable scale |  | **Manuscript**  Based on forward MR analysis, we found that ADs might have a potential causal effect on HIC. Specifically, genetically predicted AIH (ORIVW=1.09, PIVW=1.00×10-3) and RA (ORIVW=1.47, PIVW<1.00×10-4) exhibited significant positive causal effect on HIC. This result was further validated in sensitivity analyses using other MR methods. The forward MR analysis using the ConMix approach revealed potential positive causal effects of SA (ORConMix=1.16, PConMix=1.63×10-2) and T1D (ORConMix=1.37, PConMix=9.22×10-3) on HIC despite not meeting the threshold of FDR correction. However, the reverse MR analysis revealed significant positive causal effects of HIC on T1D (ORConMix=1.05, PConMix=1.77×10-3), suggesting that HIC is likely a risk factor of T1D. |
|  | b) | Report MR estimates of the relationship between exposure and outcome, and the measures of uncertainty from the MR analysis, on an interpretable scale, such as odds ratio or relative risk per SD difference |  | **Manuscript**  Genetically predicted UC (ORIVW=0.89, PIVW< 1.00×10-4) has a significant negative causal effect on the risk of HIC, which was agreed by other MR methods (PWM<1.20×10-4, PConMix=3.07×10-3) and sensitivity analysis using MR-PRESSO (ORPRESSO=0.89, PPRESSO=2.23×10-5). Additionally, a forward MR analysis using the ConMix and Egger approach revealed a potential negative causal effect of GD on HIC (ORConMix=0.81, PConMix=3.56×10-2; OREgger=0.80, PConMix=2.22×10-2). |
|  | c) | If relevant, consider translating estimates of relative risk into absolute risk for a meaningful time period |  |  |
|  | d) | Consider plots to visualize results (e.g. forest plot, scatterplot of associations between genetic variants and outcome versus between genetic variants and exposure) |  | **Fig 2**  **Supplementary Figures** |
| 12 | **Assessment of assumptions** |  |  |  |
|  | a) | Report the assessment of the validity of the assumptions |  | **Manuscript**  Reverse MR analysis revealed a positive causal effect of HIC on RA. However, after correction using the outlier test in MR-PRESSO, the aforementioned causal effects are no longer significant. Additionally, the reverse analysis revealed potential negative causal effects of HIC on HT (ORConMix=0.95, PConMix=4.98×10-2) and SLE (ORConMix=0.92, PConMix=3.58×10-2) after removing the outliers identified by the PRESSO analysis. The detailed results of the sensitivity analysis for MR can be found in Table S4 and Fig S1-116. |
|  | b) | Report any additional statistics (e.g., assessments of heterogeneity across genetic variants, such as *I^2^*, Q statistic or E-value) |  | **Supplementary table 4** |
| 13 | **Sensitivity analyses and additional analyses** |  |  |  |
|  | a) | Report any sensitivity analyses to assess the robustness of the main results to violations of the assumptions |  | **Fig2**  **Supplementary table 2-3** |
|  | b) | Report results from other sensitivity analyses or additional analyses |  | **Manuscript**  3.2 Cross-trait meta-analysis between HIC and Ads……  3.3 Fine-mapping to identify potential causal variants and colocalization analysis……  3.4 Tissue-specific enrichment analysis, pathway analysis, and eQTL mapping……  3.5 Shared genes between HIC and ADs from TWAS……  **Supplementary table 5-9** |
|  | c) | Report any assessment of direction of causal relationship (e.g., bidirectional MR) |  | **Manuscript**  Reverse MR analysis revealed a positive causal effect of HIC on RA. However, after correction using the outlier test in MR-PRESSO, the aforementioned causal effects are no longer significant. Additionally, the reverse analysis revealed potential negative causal effects of HIC on HT (ORConMix=0.95, PConMix=4.98×10-2) and SLE (ORConMix=0.92, PConMix=3.58×10-2) after removing the outliers identified by the PRESSO analysis. The detailed results of the sensitivity analysis for MR can be found in Table S4 and Fig S1-116. |
|  | d) | When relevant, report and compare with estimates from non-MR analyses |  | **Manuscript**  Additionally, we observed a significant positive causal effect of AIH and RA on HIC, which is consistent with previous epidemiological findings. A nationwide population-based study conducted in Taiwan also indicated an association between IC/BPS and the development of RA. Furthermore, our study unveiled a robust positive causal effect of HIC on T1D. In contrast, in the MR analysis, we observed a significant negative causal effect of UC on HIC, which diverges from previous epidemiological reports12. |
|  | e) | Consider additional plots to visualize results (e.g., leave-one-out analyses) |  | **Supplementay figures** |
|  | **DISCUSSION** |  |  |  |
| 14 | **Key results** | Summarize key results with reference to study objectives |  | **Manuscript**  Through bidirectional MR analysis, we investigated the causal relationships between HIC and multiple ADs. Additionally, we observed a significant positive causal effect of AIH and RA on HIC, which is consistent with previous epidemiological findings. A nationwide population-based study conducted in Taiwan also indicated an association between IC/BPS and the development of RA. Furthermore, our study unveiled a robust positive causal effect of HIC on T1D. In contrast, in the MR analysis, we observed a significant negative causal effect of UC on HIC, which diverges from previous epidemiological reports12…… |
| 15 | **Limitations** | Discuss limitations of the study, taking into account the validity of the IV assumptions, other sources of potential bias, and imprecision. Discuss both direction and magnitude of any potential bias and any efforts to address them |  | **Manuscript**  We would like to acknowledge several potential limitations in our study…… |
| 16 | **Interpretation** |  |  |  |
|  | a) | Meaning: Give a cautious overall interpretation of results in the context of their limitations and in comparison with other studies |  | **Manuscript**  Moreover, several ADs demonstrated statistical significance in the bidirectional Mendelian analysis with HIC, such as GD, SA and SLE. However, these results did not pass the multiple corrections. It is important to note that although previous epidemiological reports have highlighted the possibility of common comorbidity of Sjögren's syndrome with IC, no substantial correlation was observed in our causal inference. This discrepancy could be attributed to the relatively small sample size of GWAS studies on Sjögren's syndrome. |
|  | b) | Mechanism: Discuss underlying biological mechanisms that could drive a potential causal relationship between the investigated exposure and the outcome, and whether the gene-environment equivalence assumption is reasonable. Use causal language carefully, clarifying that IV estimates may provide causal effects only under certain assumptions |  | **Manuscript**  We also identified 64 independent loci shared between HIC and AIH, RA , and T1D at genome-wide significant level. We highlighted HLA region (several sentinel SNPs) for its significant role in between HIC and ADs. HLA region harbors more than 200 genes located close to each other on chromosome 6, one of the most extensively studies regions in human genome that contains abundant pleiotropy for many complex diseases, especially involved in the immune-related process35. The genome-wide association study has identified that three amino acid positions in human leukocyte antigen HLA-DQB1 and one amino acid position in HLA-DPB were associated with the increased risk of HIC, which revealed that genetic contributions to HIC risk that may be associated with class II MHC molecule antigen presentation. Furthermore, Tseng et al compared global gene expression profiles in bladder epithelial cells between patients with HIC and normal controls, and observed upregulations of major histocompatibility complex (MHC) class IF(HLAF) and class II (HLA-F, HLA-DQB1, HLA-DRB1, HLA-DPA1, HLA-DOA, HLA-DMA and HLA-DRA) molecules in bladder epithelial from IC and ulcerative IC area…… |
|  | c) | Clinical relevance: Discuss whether the results have clinical or public policy relevance, and to what extent they inform effect sizes of possible interventions |  | **Manuscript**  previous epidemiological reports have highlighted the possibility of common comorbidity of Sjögren's syndrome with IC, no substantial correlation was observed in our causal inference. This discrepancy could be attributed to the relatively small sample size of GWAS studies on Sjögren's syndrome….. |
| 17 | **Generalizability** | Discuss the generalizability of the study results (a) to other populations, (b) across other exposure periods/timings, and (c) across other levels of exposure |  | **Manuscript**  This study possesses several notable strengths. Firstly, most of the cross-trait studies now focus on the European population, and this is the first analysis to identify the shared genetic architecture of HIC and ADs using a large-scale observational GWAS dataset consisting exclusively of East Asian samples, after identifying ten GWAS sources (details were shown in Table S1). Furthermore, we utilized multi-omics statistical methods such as MTAG and TWAS to identify novel genes and pathways associated with both HIC and ADs. The novel genes we discovered may serve as potential drug targets for the treatment of the disease, although further validation is required. Notably, they could offer new diagnostic and therapeutic avenues for patients with HIC, particularly those with comorbid autoimmune disorders….. |
|  | **OTHER INFORMATION** |  |  |  |
| 18 | **Funding** | Describe sources of funding and the role of funders in the present study and, if applicable, sources of funding for the databases and original study or studies on which the present study is based |  | **Manuscript**  This study was funded by the National Natural Science Foundation of China (Grant No. 82270720, 32171301 and 32101206), the National Key Research and Development Program of China (Grant No. 2021YFC2009100 and 2021YFC2009102), the Natural Science Foundation of Sichuan Province (Grant No. 2022NSFSC1308), and Key Program of Science and Technology Department of Sichuan Province (Grant No. 2023YFS0102). |
| 19 | **Data and data sharing** | Provide the data used to perform all analyses or report where and how the data can be accessed, and reference these sources in the article. Provide the statistical code needed to reproduce the results in the article, or report whether the code is publicly accessible and if so, where |  | **Manuscript**  This study is a secondary analysis of existing GWAS summary data from public repositories, and international research consortia. Specific and relevant ethics approval for each of the data utilised is presented in the associated publications described in the section for GWAS summary data. No additional ethics approval is required for the conduct of the present study. |
| 20 | **Conflicts of Interest** | All authors should declare all potential conflicts of interest |  | **Manuscript**  The authors declare that the research was conducted in the absence of any commercial or financial relationships that could be construed as a potential conflict of interest. |

This checklist is copyrighted by the Equator Network under the Creative Commons Attribution 3.0 Unported (CC BY 3.0) license.

1. Skrivankova VW, Richmond RC, Woolf BAR, Yarmolinsky J, Davies NM, Swanson SA, et al. Strengthening the Reporting of Observational Studies in Epidemiology using Mendelian Randomization (STROBE-MR) Statement. JAMA. 2021;under review.

2. Skrivankova VW, Richmond RC, Woolf BAR, Davies NM, Swanson SA, VanderWeele TJ, et al. Strengthening the Reporting of Observational Studies in Epidemiology using Mendelian Randomisation (STROBE-MR): Explanation and Elaboration. BMJ. 2021;375:n2233.
